# Metabolic immune checkpoints (MICs) in cancer: from molecular mechanisms to challenges and opportunities in clinical translation

**DOI:** 10.1038/s41392-026-02903-1

**Published:** 2026-10-09

**Authors:** Jinru Yang, Xing Cai, Fajian He, Junhong Zhang, Ziqiang Zhang, Zongbi Yi, Conghua Xie, Binghe Xu

**Affiliations:** 1 Department of Radiation and Medical Oncology, Hubei Key Laboratory of Tumor Biological Behaviors, Hubei Cancer Clinical Study Center, Zhongnan Hospital of Wuhan University, Wuhan, China; 2 Department of Respiratory and Critical Care Medicine, Shanghai Pudong Hospital, Fudan University Pudong Medical Center, Pudong Hospital of Fudan University, Shanghai, China; 3 Wuhan Research Center for Infectious Diseases and Cancer, Chinese Academy of Medical Sciences, Wuhan, China; 4 Taikang Center for Life and Medical Sciences, Wuhan University, Wuhan, China; 5 Department of Medical Oncology, Beijing Key Laboratory of Innovative Drug Research and Translational Study for Breast Cancer and State Key Laboratory of Advanced Medical Materials and Devices, National Cancer Center/Cancer Hospital, Chinese Academy of Medical Sciences and Peking Union Medical College, Beijing, China

**Keywords:** Cancer metabolism, Cancer microenvironment

## Abstract

Metabolic reprogramming is a core feature of tumor cells in adapting to microenvironmental stress and acquiring malignant phenotypes. It not only supports rapid proliferation but also mediates immune escape by reshaping the immune microenvironment. In recent years, the “metabolism-immune axis”, serving as a central hub for the crosstalk between metabolic regulation and immune suppression, has provided a new perspective for understanding tumor immune evasion. This review systematically discusses how metabolic pathways, including glucose, lipid, and amino acid metabolism, along with mitochondrial function and other related processes, shape the immunosuppressive tumor microenvironment by regulating immune cell function, immune checkpoint expression, and immune signaling networks, thereby promoting tumor immune escape. The concept of “metabolic immune checkpoints (MICs)” is innovatively proposed and classified into three categories based on their mechanisms of action: (1) MIC-I: metabolic modification-type immune checkpoints, exemplified by posttranslational modifications of immune checkpoint molecules such as PD-L1 lactylation, acetylation and glycosylation, etc.; (2) MIC-II: metabolic enzyme-dependent immune regulatory nodes, represented by enzymes such as indoleamine 2,3-dioxygenase 1 and others that directly influence immune cell function; and (3) MIC-III: microbial metabolite-coupled immune regulatory targets, including receptors for microbe-derived metabolites such as short-chain fatty acid receptors. By integrating molecular interaction networks of key targets such as lactate dehydrogenase A and ATP citrate lyase, this review explores synergistic therapeutic strategies that combine small-molecule inhibitors targeting MICs with immune checkpoint blockade. This highlights how metabolic intervention can restore immune cell function and reverse exhaustion. Current key challenges in clinical translation include limitations in metabolic dynamic monitoring technologies, lack of patient stratification biomarkers and insufficient specificity in microbiome regulation. Future directions will focus on personalized treatment guided by multiomics, integrating single-cell metabolic flux analysis, deep learning-driven target prediction, and synthetic microbiome engineering, driving tumor immunotherapy into the era of precision metabolic regulation. By integrating the fundamental mechanisms of MICs with translational medical research, this review not only provides a systematic paradigm but also establishes a foundation for the development of new-generation precision combination therapies.

## Introduction

Tumor cells continuously evolve and adapt to the complex stresses of the tumor microenvironment (TME), such as hypoxia, nutrient deprivation, and immune pressure.^1,2^ Metabolic reprogramming is recognized as a core mechanism that enables tumor cells to cope with these challenges. It not only provides energy and biosynthetic precursors to support rapid proliferation but also reshapes the immune microenvironment through the regulation of metabolites intermediates and signaling networks, thereby facilitating immune evasion.^3^ Since the Warburg effect was first described, studies on tumor glucose metabolism have gradually expanded to include lipids, amino acids, and mitochondrial function from a systemic perspective.^4^ These studies have highlighted the central role of metabolic reprogramming in key tumor traits, such as enhanced proliferative capacity, increased resistance to apoptosis, and the establishment of immune tolerance.^5–7^ Metabolic gradients and spatial heterogeneity within the TME further exacerbate immune cell dysfunction, limiting the efficacy of conventional immunotherapies in certain patients and underscoring the importance of understanding tumor immune evasion through the lens of metabolic-immune crosstalk.^8–10^ Against this background, the concept of the “metabolism-immune axis” has been proposed, offering new insights into how tumors regulate immune checkpoints and immune cell function through metabolic pathways. Tumor cells suppress the activity of effector T cells, natural killer (NK) cells, and dendritic cells (DCs) by competing for nutrients, accumulating metabolic waste, and altering the physicochemical properties of the microenvironment while simultaneously inducing the polarization of immunosuppressive cells such as tumor-associated macrophages (TAMs).^11^ Glycolytic metabolites such as lactate, lipid-derived metabolites, and the depletion of specific amino acids play critical roles in these processes, not only affecting the energy supply of immune cells but also directly modulating immune signaling pathways and immune checkpoint expression, thereby forming a metabolism-driven immune evasion network.^12,13^ Recent studies have also revealed that tumors exhibit organ-specific metabolic microenvironments. For example, anabolic dominance in the liver,^14^ oxygen gradient adaptation in the lung,^15^ and the coevolution of the microbiota-host metabolic axis in the gut^16^ provide new perspectives on the role of metabolic immune checkpoints (MICs) in organ-specific tumors.

Building on these mechanistic insights, we propose the concept of MICs, which can be systematically categorized into three types. MIC-I, or metabolic modification-type immune checkpoints, regulates key molecules such as PD-L1 via posttranslational modifications, including lactylation, acetylation, and glycosylation.^17^ MIC-II comprises metabolic enzyme-dependent immune regulatory nodes, in which enzymes such as indoleamine 2,3-dioxygenase 1 (IDO1) directly modulate immune cell function through their substrates or metabolic products.^18^ MIC-III links microbial metabolites to immune regulation, exemplified by short-chain fatty acid (SCFA) receptors that integrate microbiome-derived signals with immune function.^19^ This framework distinguishes MICs from classical immune checkpoints, extending the concept from surface receptor interactions to metabolic and environmental regulation within the TME. Mapping the interplay between metabolic enzymes, metabolites, and immune checkpoint networks provides a theoretical basis for combination strategies that simultaneously target metabolic nodes and canonical checkpoint pathways.

Translating the MIC concept into clinical strategies remains challenging due to limitations in dynamic metabolic monitoring, lack of predictive biomarkers for patient stratification and insufficient specificity in microbiome modulation. Emerging approaches aim to address these gaps through single-cell and spatial multiomics analyses, real-time metabolic biosensors, and engineered microbiome interventions, which collectively enable precise modulation of metabolic gradients and immune function.^20,21^ Furthermore, metabolic interventions, including targeted inhibition of key enzymes such as lactate dehydrogenase A (LDHA) or ATP citrate lyase (ACLY), dietary modulation, and metabolically enhanced immune cell therapies, have shown potential in restoring immune competence and reversing T-cell exhaustion.^22–24^ Integrating these strategies with immunotherapies, informed by high-resolution metabolic maps and predictive computational modeling, may facilitate individualized precision interventions.

Accordingly, this review will first systematically summarize tumor metabolic reprogramming and its immunoregulatory roles, elaborate on the three-category classification of MICs, their core mechanisms, and the key networks linking metabolic reprogramming to immune evasion, explore MIC characteristics and dominant pathways within organ-specific metabolic microenvironments, and analyze clinical translation strategies. By integrating mechanistic insights with potential clinical applications, this review aims to provide a conceptual and practical framework for developing future metabolism-immune combination therapies.

## Metabolic reprogramming and immune regulation in the tumor microenvironment

### Overview of metabolic reprogramming

The development and progression of tumors depend not only on the malignant transformation of tumor cells themselves but also on the TME in which they reside. The TME is a complex ecosystem composed of tumor cells, immune cells, blood vessels, signaling molecules, and metabolic products, among others.^25,26^ Recent studies have shown that metabolic reprogramming is a core hallmark of tumors.^27^ Within the TME, this concept carries a dual meaning. On the one hand, it refers to the fundamental remodeling of core metabolic pathways in tumor cells driven by oncogenic signals. This involves altering the metabolism of nutrients such as glucose, lipids, and amino acids to adapt to harsh conditions such as hypoxia, acidosis, and nutrient scarcity while meeting the energy and material demands for rapid proliferation, invasion, metastasis, and immune evasion.^28–30^ On the other hand, it also encompasses the adaptive metabolic changes in immune cells in response to metabolic pressures within the TME, such as nutrient competition and the accumulation of metabolic waste,^31,32^ a phenomenon known as immunometabolic plasticity. These two forms of reprogramming are intricately intertwined and dynamically interact within the TME, collectively shaping an immunosuppressive ecosystem and determining the success or failure of antitumor immunity. This section will first focus on the intrinsic metabolic reprogramming of tumor cells as the initiating factor driving the imbalance in the metabolic landscape of the entire TME.

#### Historical context: from Warburg to a contemporary systemic view

The understanding of cancer metabolism began a century ago. In the 1920s, Otto Warburg observed that tumor tissues extensively take up glucose and produce lactate even in the presence of ample oxygen, a phenomenon now known as the Warburg effect or aerobic glycolysis.^33–35^ This discovery laid the foundation for the recognition of aberrant tumor metabolism, yet it was long misinterpreted as a mere byproduct of defective mitochondrial function.^36^ Over the past two decades, with the advancement of molecular biology and metabolomics technologies, it has become clear that metabolic reprogramming is far from a simple anomaly in energy production. Instead, it is a highly coordinated, adaptive program actively driven by oncogenes (e.g., MYC, RAS, PI3K/AKT) and the inactivation of tumor suppressor genes (e.g., p53).^37^ Its core objective is to support the biosynthesis of macromolecules, thereby meeting the demand for building materials required for rapid cell proliferation.

#### Core principles of metabolic reprogramming

The metabolic reprogramming of tumor cells embodies three synergistic core principles. First, there is a fundamental shift in the metabolic network from catabolism toward anabolism. Unlike quiescent cells, which primarily rely on catabolism to meet basic energy demands, tumor cells redirect a significant metabolic flux into anabolic pathways. Their metabolic network operates like a bustling assembly line, continuously producing essential building materials for cell proliferation, such as nucleic acids, proteins, and lipids. Second, there is remarkable metabolic plasticity and adaptability. Tumor cells do not adhere to a single metabolic mode but exhibit extraordinary flexibility in responding to a hostile microenvironment. Significant metabolic heterogeneity exists among different tumor subpopulations and even within the same tumor. For example, certain CD44^+^ cancer stem cell (CSC) subpopulations located in well-oxygenated regions may still predominantly rely on efficient oxidative phosphorylation to meet their energy needs.^38^ This plasticity enables tumor cells to dynamically switch metabolic pathways in response to fluctuations in key nutrient availability: when glucose is limited, they can initiate fatty acid oxidation (FAO) for energy production and membrane lipid synthesis or utilize glutamine as a carbon and nitrogen source to support biosynthesis.^39,40^ These dynamic adaptations are not isolated events but form a highly coordinated and adaptive metabolic network, allowing tumor cells to sustain proliferation, acquire invasive potential, and develop therapy resistance under pressures such as nutrient scarcity and hypoxia. Finally, there is a signaling regulatory function of metabolites. Increasing evidence shows that many classic metabolic intermediates (such as α-ketoglutarate, succinate, fumarate, and acetyl-CoA) have evolved beyond their roles as substrates to become important signaling molecules. These metabolites regulate the activity of epigenetic-modifying enzymes, including histone demethylases, DNA methyltransferases, and acetyltransferases, through mechanisms such as allosteric modulation, serving as cofactors, or directly participating in catalytic reactions. Consequently, they profoundly influence gene expression profiles and cell fate decisions, tightly coupling metabolic states with cellular functional regulation.^41^

#### Central role of metabolic reprogramming in the hallmarks of cancer

Metabolic reprogramming itself constitutes one of the core hallmarks of cancer and is widely recognized as deregulating cellular energetics.^42–44^ Serving as the metabolic foundation for tumor initiation and progression, it not only enables tumor cells to survive and proliferate in harsh microenvironments but also systematically supports and drives the acquisition of all other cancer hallmarks. Specifically, it provides essential biosynthetic precursors and energetic support for sustaining proliferative signaling and resisting cell death. It contributes to genome instability and mutation through mechanisms such as reactive oxygen species (ROS) accumulation induced by metabolic abnormalities and the actions of specific metabolic intermediates.^45^ It promotes the induction of angiogenesis by activating pathways such as hypoxia-inducible factor 1-alpha (HIF-1α).^46^ It facilitates invasion and metastasis through microenvironment acidification and the supply of metabolic precursors.^47^ Most critically, it directly contributes to immune evasion and tumor-promoting inflammation by competitively depleting key nutrients such as glucose and tryptophan and by secreting immunosuppressive metabolites such as lactate and kynurenine.^48^ Therefore, metabolic reprogramming is not merely a core metabolic strategy for tumor cell autonomy, adaptation, and survival; it is also a key regulatory mechanism through which tumor cells actively shape an immunosuppressive microenvironment and achieve immune evasion, thereby providing an indispensable metabolic basis for malignant progression. A deep understanding of these metabolic features, the cooperative networks among pathways, and their regulation of immune function will provide crucial theoretical foundations and potential therapeutic targets for developing integrated treatment strategies that combine tumor metabolism and immune checkpoint modulation.

### Characteristics and mechanisms of tumor cell metabolic reprogramming

Tumor cell metabolic reprogramming represents a fundamental transformation of malignant properties. This reprogramming is not merely an increase in energy production but rather a highly coordinated, multidimensional adaptive program designed to support sustained proliferation, survival, and dissemination. Its features can be summarized into three core principles: first, anabolism predominance, whereby metabolic flux is redirected from catabolism (energy production) toward anabolism (the biosynthesis of macromolecules); second, metabolic plasticity, allowing flexible switching among different metabolic pathways in response to changes in the microenvironment; and third, metabolites as signaling molecules, in which many metabolic intermediates transcend their classical metabolic roles and directly participate in the regulation of gene expression and cell fate. These characteristics are reflected throughout the remodeling of all major metabolic pathways.

#### Reprogramming of glucose metabolism

Reprogramming of glucose metabolism is the most well-known hallmark of tumor metabolism, with its core feature being the Warburg effect, that is, the preferential use of lactate fermentation even under oxygen-sufficient conditions.^33–35^ This seemingly inefficient metabolic choice confers profound functional advantages. It enables the rapid generation of ATP; although the ATP yield per molecule of glucose is relatively low, the extremely high metabolic flux is sufficient to meet the basal energy demands of the cell. More importantly, highly active glycolysis provides essential precursor molecules for anabolic processes. Multiple key enzymes within the glycolytic pathway undergo specific regulation at both the expression and functional levels. Hexokinase 2 (HK2), located on the cytoplasmic side of the mitochondrial outer membrane, rapidly phosphorylates glucose entering the cell to glucose-6-phosphate (G6P). This reaction not only initiates glycolysis but also effectively traps glucose within the cell, preventing its efflux.^49,50^ This high-affinity phosphorylation represents the first critical step in maintaining the high glycolytic flux characteristic of tumor cells.

The M2 isoform of pyruvate kinase (PKM2) functions as a metabolic valve. Unlike the constitutively active PKM1 isoform, PKM2 activity is finely regulated by a variety of allosteric effectors.^51,52^ In tumor cells, PKM2 is often maintained in a low-activity state, leading to the accumulation of upstream metabolic intermediates, such as phosphoenolpyruvate (PEP), which are then diverted into biosynthetic side pathways.^53^ This represents a sophisticated design of metabolic reprogramming: by slowing flux at the terminal step of glycolysis, carbon skeletons are redirected toward biosynthetic processes where they are most needed. These branch pathways include the pentose phosphate pathway (PPP) and the hexosamine biosynthetic pathway (HBP). The PPP is another pathway aberrantly activated in tumor cells. Through two major functional outputs, it generates critical metabolites: first, ribose-5-phosphate (R5P), which is indispensable for nucleotide synthesis; and second, large amounts of reduced nicotinamide adenine dinucleotide phosphate (NADPH), which is essential for fatty acid synthesis and for maintaining intracellular redox homeostasis, thereby protecting cells against oxidative stress.^54^ The hexosamine biosynthetic pathway, by contrast, channels a portion of fructose-6-phosphate (F6P) into the production of UDP-N-acetylglucosamine.^55^ This metabolite serves as the donor substrate for protein O-GlcNAcylation. This dynamic form of glycosylation broadly influences the activity of transcription factors, signaling proteins, and chromatin-regulating factors, thereby constituting a critical bridge linking cellular metabolism to epigenetic regulation.

The terminal product of glycolysis is lactate,^56^ whose formation is catalyzed by LDHA. High expression of LDHA ensures the rapid conversion of pyruvate to lactate while simultaneously regenerating NAD^+^,^57^ which is required to sustain glycolytic flux. This robust lactate production results in its substantial accumulation and secretion into the extracellular space, which represents a major source of TME acidification. This acidic environment itself acts as a protumorigenic and immunosuppressive factor.^58^ In addition, certain glycolytic intermediates, such as PEP, can be converted into oxaloacetate through anaplerotic reactions. This replenishes tricarboxylic acid (TCA) cycle intermediates that are extensively withdrawn for the synthesis of amino acids and other biomolecules,^59,60^ thereby maintaining the operation of this central metabolic cycle.

#### Reprogramming of lipid metabolism

Tumor lipid metabolic reprogramming is characterized by a coordinated rewiring of lipid synthesis, uptake, storage, oxidation, and signaling pathways, enabling tumor cells to adapt to fluctuating nutrient availability and microenvironmental stress. Reprogramming of lipid metabolism is essential for supporting the rapid proliferation of tumor cells, as lipids are not only the principal components of all cellular membranes^61^ but also serve as important signaling molecules and forms of energy storage.^62^ Tumor cells exhibit a markedly increased demand for lipids and fulfill this requirement through multiple, tightly regulated metabolic strategies. De novo fatty acid synthesis (FAS) represents a core strategy for lipid supply in tumor cells.^63^ This process is initiated by ACLY, which transports citrate generated by the TCA cycle into the cytosol and cleaves it into acetyl-CoA and oxaloacetate. Cytosolic acetyl-CoA is subsequently carboxylated by acetyl-CoA carboxylase (ACC) to form malonyl-CoA. Fatty acid synthase (FASN), a large multifunctional enzyme complex, then catalyzes successive rounds of condensation, reduction, dehydration, and reduction reactions, using acetyl-CoA as the primer and malonyl-CoA as the two-carbon donor, ultimately producing saturated fatty acids such as palmitate.^64^ This de novo synthetic process is highly dependent on energy and reducing power, consuming large amounts of NADPH supplied by the pentose phosphate pathway, thereby highlighting the close coordination between lipid metabolism and other metabolic pathways.

In addition to enhancing endogenous FAS, tumor cells expand lipid availability and increase metabolic flexibility through alternative mechanisms. On the one hand, tumor cells can upregulate scavenger receptors such as CD36 to efficiently uptake circulating lipoproteins or fatty acids released from adjacent adipocytes.^65^ On the other hand, under unfavorable microenvironmental conditions such as severe hypoxia or glucose deprivation, tumor cells may activate FAO to sustain energy production. This process is initiated by carnitine palmitoyltransferase 1 (CPT1), which transports activated long-chain fatty acids into the mitochondrial matrix for β-oxidation, generating acetyl-CoA, NADH, and FADH_2_. These metabolites subsequently enter the TCA cycle and oxidative phosphorylation to produce ATP, thereby enhancing tumor cell survival under metabolic stress.^66^

A fraction of fatty acids synthesized or acquired by tumor cells is stored as triglycerides (TG) within lipid droplets, dynamic organelles that function beyond inert energy reservoirs. Lipid droplets act as active metabolic hubs involved in membrane lipid supply, signal transduction, and cellular stress responses.^67^ Moreover, tumor cells exploit lipid metabolic pathways to generate a variety of bioactive lipid mediators. Among them, prostaglandin E2 (PGE2) is one of the most important immunomodulatory lipids.^68^ Elevated expression of cyclooxygenase-2 (COX-2) in tumor cells promotes the conversion of arachidonic acid to PGE2. Through autocrine and paracrine signaling, PGE2 stimulates tumor cell proliferation and angiogenesis while suppressing DC maturation, T-cell activation, and NK cell function, thereby serving as a key mediator in the establishment of an immunosuppressive tumor microenvironment.^69^ In addition, other lipid signaling molecules, such as lysophosphatidic acid (LPA) and sphingosine-1-phosphate (S1P), are widely involved in the regulation of tumor growth and metastasis.^70,71^ LPA activates the PI3K/AKT pathway via its G-protein coupled receptors (LPAR1-6),^72^ promoting tumor cell survival, proliferation, and migration. AKT, in turn, feedback regulates LPA signaling by modulating receptor activity and LPA synthesis mediated by enzymes such as autotaxin (ATX).^73^ Moreover, LPA triggers the NF-κB pathway through calcium ion flux and PKC activation, leading to the release of NF-κB from IκB.^74,75^ Activated NF-κB enhances the transcription of genes involved in tumor growth, invasion, and metastasis while also upregulating ATX expression, thereby establishing a positive feedback loop that accelerates tumor progression.

#### Amino acid metabolic reprogramming

Amino acid metabolic reprogramming in cancer is a multifaceted adaptation that encompasses dysregulated uptake, enhanced biosynthesis, and rewired catabolism of both essential and non-essential amino acids.^76^ This coordinated rewiring sustains anabolic growth, maintains redox homeostasis, and actively shapes an immunosuppressive TME. This reprogramming is initiated at the plasma membrane through the overexpression of specific amino acid transporters. Notably, the glutamine transporter SLC1A5 (ASCT2), the broad neutral amino acid transporter SLC7A5 (LAT1) (often in complex with SLC3A2) for essential amino acids, and the cystine/glutamate antiporter SLC7A11 (xCT) are frequently upregulated in tumors.^77^ These alterations ensure a robust supply of nitrogen, carbon, sulfur, and essential building blocks even under nutrient stress. Concurrently, tumor cells enhance the synthesis of non-essential amino acids (e.g., serine, glycine, proline) and exhibit profound reprogramming of key catabolic pathways, as detailed below.

Glutamine, the most abundant free amino acid in the circulation, is taken up by tumor cells at exceptionally high rates,^78^ a process often driven by the marked upregulation of glutamine transporters such as ASCT2 (SLC1A5) to ensure sufficient acquisition in the nutrient-scarce TME. Under the catalysis of glutaminase, glutamine is deamidated to generate glutamate. Glutamate can then be converted into α-ketoglutarate through transamination reactions or via glutamate dehydrogenase, thereby replenishing the TCA cycle. This anaplerotic input is essential for maintaining TCA cycle flux, as its intermediates are continuously withdrawn for the biosynthesis of aspartate, citrate, and other anabolic precursors.^79^ Importantly, glutamine also serves as the primary nitrogen source for tumor cells: its amide nitrogen is widely utilized in the synthesis of purines, pyrimidines, and multiple non-essential amino acids, such as proline and arginine, thereby supporting robust nucleic acid and protein biosynthesis.^80^

Serine and glycine metabolism is tightly coupled through the one-carbon metabolic network.^81^ Serine hydroxymethyltransferase catalyzes the conversion of serine and tetrahydrofolate into glycine and 5,10-methylene-tetrahydrofolate, the latter functioning as a carrier of activated one-carbon units. One-carbon metabolism provides essential formyl and methylene groups for de novo purine synthesis and thymidylate production, representing a key rate-limiting process in nucleotide biosynthesis.^82^ In addition, one-carbon units feed into the S-adenosylmethionine cycle, supporting methylation reactions of DNA, RNA, and histones. Through these mechanisms, amino acid metabolism directly links the cellular metabolic state to epigenetic regulation of gene expression and transcriptional silencing.^83^

Tryptophan metabolism plays a central role in tumor immune evasion. IDO1, which is highly expressed in tumor cells or tumor-associated stromal cells, is the rate-limiting enzyme for tryptophan degradation along the kynurenine pathway.^84^ Activation of IDO1 leads to local depletion of tryptophan within the tumor microenvironment, thereby activating the stress-sensing kinase general control nonderepressible 2 (GCN2) in T cells, suppressing protein translation, and inducing T-cell dysfunction or apoptosis.^85^ Meanwhile, kynurenine, the major metabolic product of this pathway, functions as a potent immunomodulatory molecule. By activating the aryl hydrocarbon receptor (AhR), kynurenine (Kyn) directly inhibits the proliferation and effector functions of cytotoxic T cells and NK cells while promoting the differentiation of regulatory T (Treg) cells, collectively establishing immune tolerance at multiple levels.^86^

Arginine metabolism likewise exhibits pronounced bidirectional regulatory properties. Arginine can be hydrolyzed by arginase to generate ornithine, which serves as the initiating substrate for polyamine synthesis. Polyamines are essential for supporting rapid tumor cell proliferation.^87^ Conversely, arginine can also be metabolized by inducible nitric oxide synthase (iNOS) to produce nitric oxide (NO). Low concentrations of NO may promote vasodilation and angiogenesis, whereas high levels exert cytotoxic effects.^88^ These context-dependent outcomes underscore the complex and dynamic role of arginine metabolism in tumor biology.

#### Reprogramming of mitochondrial function

Although the Warburg effect is a prominent feature, mitochondria are not discarded in tumor cells. Instead, they undergo functional reprogramming, evolving from energy factories into hubs that integrate metabolic regulation and signaling.^89^ Accumulating evidence indicates that tumors actively exploit this reprogramming, dynamically switching between glycolysis and oxidative phosphorylation (OXPHOS) in response to survival states and therapeutic pressures, thereby acquiring superior metabolic plasticity.

To support this flexibility, mitochondrial OXPHOS remains active in many tumor types. In specific tumor cell subpopulations, such as therapy-resistant cells and CD44^+^ CSCs,^38^ OXPHOS constitutes a major energy source for survival and maintenance of stemness.^90,91^ However, the electron transport chain (ETC) is frequently dysfunctional in tumors, which can lead to excessive production of ROS.^92^ Notably, ROS play a dual role: moderately elevated levels function as signaling molecules to promote tumor cell proliferation and survival, whereas excessive ROS induce oxidative damage and cell death.^93^ Therefore, tumor cells finely regulate mitochondrial ROS production to harness their pro-proliferative signaling while avoiding cytotoxic oxidative stress. Furthermore, to maintain mitochondrial network integrity under such metabolic and therapeutic stress, tumor cells tightly regulate mitochondrial dynamics through coordinated fusion and fission processes: fusion facilitates the mixing of mitochondrial contents and functional complementation, while fission enables the segregation of damaged mitochondria for removal via mitophagy.^94^ This mitochondrial quality control system is essential for maintaining cellular homeostasis.

Beyond maintaining bioenergetic and redox balance, mitochondrial reprogramming extends to direct regulation of nuclear gene expression. Tumor-associated genetic alterations, such as loss-of-function mutations in succinate dehydrogenase (SDH) or fumarate hydratase (FH), can lead to abnormal accumulation of specific TCA cycle intermediates, including succinate and fumarate.^95^ These so-called oncometabolites act as competitive inhibitors of a family of α-ketoglutarate-dependent dioxygenases, including prolyl hydroxylases and multiple histone and DNA demethylases.^96^ Inhibition of these enzymes results in aberrant stabilization of HIF-1α even under normoxic conditions and induces a global hypermethylated epigenetic state, thereby driving sustained activation of tumor-associated transcriptional programs.^97^ Thus, mitochondrial metabolic dysfunction can be converted into stable epigenetic and transcriptional reprogramming, directly linking mitochondrial alterations to tumor initiation and progression.

Finally, mitochondria also serve as pivotal communicators with the TME. Under certain pathological or stress conditions, such as collapse of the mitochondrial membrane potential, opening of the mitochondrial permeability transition pore, or exposure to specific anticancer therapies, mitochondrial DNA (mtDNA) may be released into the cytosol. As a damage-associated molecular pattern (DAMP), mtDNA can be recognized by the cytosolic DNA sensor cyclic GMP-AMP synthase (cGAS), leading to activation of the downstream stimulator of interferon genes (STING) signaling pathway and induction of type I interferons (IFN-I) and other inflammatory mediators.^89,98^ In principle, this pathway can trigger antitumor innate immune responses; however, within the TME, its immune-stimulatory effects are often attenuated or subverted by tumor cells through downregulation of STING expression or other adaptive mechanisms. This illustrates the profound extent of mitochondrial reprogramming, enabling tumor cells to co-opt even stress-induced signaling for survival advantage.

#### Other metabolic reprogramming pathways

To sustain continuous and rapid proliferation, nucleotide metabolism is broadly upregulated in tumor cells to support DNA replication, RNA transcription, and related biosynthetic processes.^99^ This upregulation reflects a coordinated reprogramming of nucleotide biosynthetic pathways that enables tumor cells to meet the heightened demands of uncontrolled growth. Nucleotide supply is mainly derived from two pathways: de novo synthesis and salvage synthesis, which exist in a dynamic balance depending on metabolic conditions and tumor type.^100^ De novo nucleotide synthesis relies heavily on the coordinated activity of multiple metabolic pathways. The PPP provides R5P as the structural backbone for nucleotide assembly,^54^ while one-carbon metabolism supplies essential carbon units required for purine ring formation and methylation reactions during thymidylate synthesis.^101^ In addition, de novo synthesis requires substantial ATP input and depends on amino acids such as glutamine as nitrogen donors, making this pathway particularly active under conditions of sufficient energy and nutrient availability.^102^ Importantly, tumor cells dynamically regulate de novo nucleotide synthesis in response to proliferative cues and metabolic status, highlighting its central role in anabolic growth. Concurrently, tumor cells extensively utilize the nucleotide salvage pathway, which recycles bases and nucleosides generated from nucleic acid degradation to synthesize nucleotides at a lower energetic cost.^103^ This pathway becomes especially important under nutrient-limited or energy-stressed conditions, contributing to improved metabolic efficiency and maintenance of nucleic acid homeostasis. Under certain pathological states or metabolic stresses, such as prolonged nutrient deprivation or in specific tumor types, tumor cells may also exhibit the capacity to utilize ketone bodies, including β-hydroxybutyrate and acetoacetate, as alternative energy sources or signaling molecules. Ketone bodies can be converted into acetyl-CoA to fuel energy metabolism and may also act as signaling metabolites that influence gene expression and cell fate decisions.^104,105^ Utilization of these noncanonical energy substrates further underscores the remarkable adaptability and plasticity of tumor metabolic networks within complex microenvironments.

#### Metabolic gradients and spatial heterogeneity in the TME

The TME exhibits pronounced spatial heterogeneity, and metabolic gradients represent one of the key driving forces underlying this heterogeneity.^106^ Owing to the disorganized, leaky, and functionally inefficient tumor vasculature, the delivery of oxygen and nutrients is severely compromised, resulting in a limited effective diffusion distance within tumor tissues.^107^ Meanwhile, the persistently high metabolic activity of tumor cells generates large amounts of metabolic waste products.^108^ Together, these factors give rise to stable metabolic gradients radiating from blood vessels into the tumor core: oxygen and glucose concentrations progressively decline, whereas lactate and CO_2_ accumulate, leading to a transition from a relatively neutral pH in perivascular regions to an acidic environment in areas distal to blood vessels. These overlapping physicochemical gradients actively shape tumor biology by creating spatially distinct microecological niches and imposing differential selective pressures on tumor cells, thereby driving substantial intratumoral heterogeneity. Tumor cells located near blood vessels experience relatively sufficient oxygen and nutrient availability and tend to rely more on mitochondrial oxidative phosphorylation, exhibiting higher proliferative activity. In contrast, cells located farther from the vasculature and exposed to hypoxic and acidic conditions predominantly depend on glycolysis. Their transcriptional programs are frequently remodeled by pathways such as HIF-1α, conferring enhanced invasiveness, stem-like properties, or a quiescent phenotype. These cell populations are often intrinsically resistant to oxygen-dependent radiotherapy and certain chemotherapeutic agents, making them a major source of treatment failure and tumor relapse.^109,110^ Metabolic gradient-driven intratumoral heterogeneity markedly increases the complexity of clinical treatment, as a single therapeutic strategy is unlikely to eliminate all tumor cell subpopulations simultaneously. Consequently, resolving the spatial organization and cellular composition of metabolic heterogeneity is critical for the development of more effective therapeutic approaches. In recent years, advances in single-cell metabolomics and spatial metabolomics have provided powerful tools to systematically dissect this complexity.^111–113^

Single-cell metabolomics enables the characterization of intracellular metabolite profiles at single-cell resolution, thereby revealing metabolic subpopulations that are obscured in conventional bulk analyses.^114,115^ Using techniques such as mass cytometry (CyTOF), time-of-flight secondary ion mass spectrometry (TOF-SIMS), and single-cell nuclear magnetic resonance, researchers can simultaneously quantify the relative abundance of multiple key metabolites within individual cells.^116^ These studies demonstrate that even within spatially proximate tumor regions, substantial metabolic heterogeneity exists among tumor cells as well as between tumor cells and immune or stromal cells.^117,118^ For example, within the same tumor, some cancer cells exhibit a strong dependence on glycolysis, whereas others preferentially rely on fatty acid oxidation.^119^ Similarly, among infiltrating CD8^+^ T cells, only a subset retains effective metabolic activity, while the majority experience metabolic suppression due to nutrient competition.^120^ Such cell-level metabolic differences directly influence cellular behavior and therapeutic responsiveness. Building upon this, spatial metabolomics integrates metabolic information with tissue architecture, further revealing how metabolic heterogeneity is organized at the tissue scale.^121,122^ Techniques such as matrix-assisted laser desorption/ionization mass spectrometry imaging (MALDI-MSI) and desorption electrospray ionization mass spectrometry imaging (DESI-MSI) enable the visualization of metabolite distributions in tumor sections while preserving tissue integrity.^123^ These metabolic maps allow direct observation of lactate enrichment in necrotic or hypoxic regions, rapid depletion of glutamine in perivascular zones, and selective accumulation of specific lipid species at invasive fronts. Integration with spatial transcriptomics further enables the correlation of metabolic states with gene expression programs, facilitating spatially resolved analysis of metabolic-phenotypic colocalization. For instance, regions with elevated HIF-1α expression can be examined in relation to local lactate accumulation, or specific metabolic tumor cell subpopulations can be analyzed in the context of their proximity to immune cell clusters.

The complexity revealed by these approaches provides a conceptual framework for the development of precision combination therapies that target metabolic heterogeneity. Based on single-cell and spatial metabolic maps, key metabolic subpopulations responsible for therapeutic resistance can be identified and selectively targeted. For example, glycolytic inhibitors may be used to target cells in hypoxic tumor cores, while inhibitors of FAO or glutamine metabolism may be applied to other metabolically distinct subpopulations. In addition, short-term combination with antiangiogenic therapies to transiently normalize tumor vasculature may improve perfusion and oxygenation, thereby enhancing drug delivery, sensitizing hypoxic cells to radiotherapy, and potentially improving immune cell infiltration. Furthermore, direct modulation of the TME, such as regulating extracellular pH through carbonic anhydrase inhibitors or adjusting redox balance, may disrupt the survival advantages of specific metabolic niches, rendering them more susceptible to elimination by other therapeutic modalities. Ultimately, such multitarget, spatiotemporally informed combination strategies hold promise for overcoming treatment resistance driven by metabolic heterogeneity.

### Tumor metabolic reprogramming and its regulation of immune checkpoints and immune cell functions

#### Metabolic regulation of immune checkpoint molecules by tumor cells

Tumor cells actively reprogram their metabolic networks not only to sustain proliferation but also to fine-tune the expression, stability, and signaling activity of immune checkpoint molecules. Through coordinated regulation at the levels of metabolic enzymes, signaling pathways, and metabolite accumulation, tumor cells establish a metabolically driven immune checkpoint network that underpins immune evasion within the TME.

Metabolic reprogramming can directly influence the transcription and translation of immune checkpoint molecules. For instance, activation of ACLY-mediated FAO induces mtDNA damage and release, activates the cGAS-STING signaling pathway, and upregulates PD-L1 expression in tumor cells, ultimately impairing T-cell effector function.^124^ Additionally, cholesterol metabolism has been shown to modulate immune checkpoint expression. Targeting cholesterol-25-hydroxylase (Ch25h) effectively alleviates immunosuppression within the TME, enhances T-cell infiltration and activation, and synergizes with anti-PD-1 therapy to significantly improve antitumor immune responses.^125^ Metabolic alterations also regulate immune checkpoint proteins through posttranslational modifications that affect their stability and function. In glucose metabolism, lactate accumulation mediated by LDHA stabilizes the PD-L1 protein, thereby promoting immune evasion by tumor cells. Notably, baicalin has been shown to target the IGF2BP3/LDHA axis, reduce PD-L1 stability, and reverse lactate-driven immunosuppressive effects.^126^ Similarly, claudin-9 (CLDN9) activates the PI3K/AKT/HIF-1α signaling pathway, upregulates the expression of LDHA and monocarboxylate transporter 1 (MCT1), and enhances glycolytic flux.^127^ This metabolic reprogramming not only facilitates PD-L1 lactylation and protein stabilization but also markedly suppresses the antitumor function of CD8^+^ T cells. Certain metabolites function directly as metabolic immune checkpoints, suppressing antitumor immunity through receptor-mediated signaling or by modulating immune cell function. For example, kynurenine, generated via IDO1-mediated tryptophan catabolism, induces T-cell exhaustion and synergizes with classical checkpoint pathways.^128^ Lactate, a major byproduct of tumor glycolysis and a key oncometabolite, not only stabilizes PD-L1 but also promotes M2 macrophage polarization via G protein-coupled receptor 81 (GPR81),^129^ further reinforcing an immunosuppressive microenvironment.

Collectively, tumor cells reshape immune checkpoint networks through multilayered metabolic regulation involving transcriptional control, protein modification, and metabolite signaling.^130^ This metabolism-driven immunomodulation represents a major challenge to current immunotherapies while also offering promising targets for combined metabolic-immune intervention strategies.

#### Metabolic reprogramming of immune cells and functional suppression in the TME

Beyond directly modulating immune checkpoint molecules, tumor cell metabolism actively shapes the TME into a nutrient-deprived, hypoxic, and metabolite-rich compartment. This metabolic landscape exerts profound and selective pressure on infiltrating immune cells. The activation, differentiation, and effector functions of immune cells are intrinsically dependent on finely tuned metabolic programs. However, the metabolically hostile TME systematically disrupts these programs, ultimately leading to widespread immune dysfunction and suppression. While immune cells possess metabolic plasticity to adapt their fuel preferences to functional states, this adaptability is often hijacked within the TME. The result is a paradoxical outcome: effector functions are impaired in antitumor immune cells (e.g., cytotoxic CD8^+^ T cells, M1-like macrophages, and conventional dendritic cells [cDCs]), while immunosuppressive cell populations (e.g., Tregs, myeloid-derived suppressor cells [MDSCs], TAMs, and exhausted T cells [Tex]) gain a survival advantage. Importantly, recent single-cell and spatial multiomics studies^131,132^ have demonstrated that broad immune cell classifications are insufficient to capture TME heterogeneity. Each major immune lineage encompasses diverse subsets with distinct metabolic states, functional programs, spatial localization, and therapeutic relevance. This section therefore examines how tumor metabolic reprogramming shapes immune cell function, highlighting major populations, including T cells, B cells, NK cells, and myeloid cells, as well as specific subsets, such as naïve T cells (Tn), effector T cells (Teff), memory T cells (Tmem), exhausted T cells (Tex), regulatory T cells (Tregs), tissue-resident memory T cells (Trm), follicular helper T cells (Tfh), unconventional γδ T cells, mucosal-associated invariant T (MAIT) cells, naïve B cells (Bn), germinal center B cells (Bgc), plasma cells (PCs), memory B cells (Bmem), regulatory B cells (Bregs), resting NK cells, activated NK cells, CD56^bright^ NK cells, CD56^dim^ NK cells, TAMs with M1-like and M2-like phenotypes, immature DCs, conventional DC subsets (cDC1, cDC2), plasmacytoid DCs (pDCs), inflammatory DCs (inf-DCs), MDSCs, and tumor-associated neutrophils (TANs), emphasizing their functional diversity and metabolic dependencies. A systematic summary of the functional characteristics, metabolic phenotypes, and TME-induced effects for each of these subsets is provided in Table 1.

**Table 1:** Immune cell metabolic programs and TME-induced reprogramming

Cell Type	Subset	Functional Characteristics	Metabolic Phenotype	Effect of TME on Subset
T-cell	Tn	Maintain quiescence, long-term survival, ready for activation	OXPHOS; low glycolysis; low FAO	Nutrient deprivation may impair activation capacity upon antigen encounter
	Teff	Clonal expansion; produce IFN-γ, GZMB	High aerobic glycolysis; high glutaminolysis	Hypoxia stabilizes HIF-1α (promotes glycolysis but may drive dysfunction); chronic activation leads to metabolic exhaustion
	Tmem	Long-term survival; self-renewal; rapid recall response	FAO; enhanced mitochondrial biogenesis	TME metabolic stress (low glucose, high lactate) may impair memory differentiation and survival
	Tex	Impaired cytotoxicity; high PD-1/TIM-3	Mitochondrial dysfunction; diminished glycolysis; elevated oxidative stress	Low glucose, high lactate, tryptophan depletion, and kynurenine drive exhaustion; metabolic stress reinforces PD-1^high^ TIM-3^high^ phenotype
	Treg	Immunosuppression; survive metabolic stress	OXPHOS; FAO; lactate utilization (LDHB → TCA)	Low glucose, high lactate, high kynurenine preserve or enhance Treg survival and function, exacerbating immune suppression
	Trm	Tissue residence; localized cytotoxicity	Enhanced FAO; mevalonate-cholesterol synthesis; mitochondrial adaptability	TGF-β helps maintain Trm state; Trm cells promote TIL accumulation and enhance immunotherapy efficacy
	Tfh	Help B cells in germinal center responses	Enhanced glycolysis; enhanced lipid metabolism	Glucose deprivation may impair Tfh function and subsequent humoral immunity
	γδ T			
	IFN-γ^+^ γδ T-cell	Antitumor effector	Glycolysis	High glucose enhances IFN-γ^+^ γδ T-cell proliferation and antitumor activity
	IL-17^+^ γδ T-cell	Pro-tumor (promotes tumor growth)	Mitochondrial oxidative metabolism	High-lipid environment selectively expands IL-17^+^ γδ T cells, promoting tumor growth
	MAIT	Rapid cytokine production (IFN-γ, IL-17)	Enhanced glycolysis	Tumor-derived tryptophan metabolites → AhR promote glycolysis; forms pro-tumor feedback loop (enhances tumor lipid storage/proliferation)
B-cell	Bn	Maintain homeostasis, metabolically quiescent	Relatively low glycolysis and OXPHOS	TME metabolic stress may disrupt activation potential
	Bgc	Clonal expansion, immunoglobulin class switching, somatic hypermutation	High-flux state dominated by aerobic glycolysis and glutaminolysis; driven by BCR and CD40 signaling	Glucose limitation directly suppresses glycolytic flux, inhibiting proliferation and antibody synthesis
	PC	Large-scale immunoglobulin synthesis and secretion	Active glycolysis; heavy reliance on ER and mitochondrial biosynthetic capacity; OXPHOS supports disulfide bond formation	Glucose deprivation inhibits antibody production; lactate accumulation and acidosis disrupt B-cell signaling and survival
	Bmem	Long-term survival, rapid recall response	Enhanced FAO and OXPHOS; supported by AMPK signaling	TME metabolic stress may impair memory differentiation and survival
	Breg	Immunosuppression (e.g., IL-10 production)	Preference for OXPHOS (potential greater adaptability under metabolic stress)	Metabolically adaptable Breg subsets may expand or gain functional dominance, indirectly reinforcing immunosuppression
NK cell	Resting NK	Maintain basal homeostasis and survival	Primarily OXPHOS; glycolytic and anabolic pathways are quiescent	Nutrient deprivation and hypoxia impair basal metabolic fitness
	CD56^bright^ NK	Mainly produce cytokines (IFN-γ, TNF-α)	Lower metabolic activity	TME metabolic stress may skew subset balance and impair cytokine production
	CD56^dim^ NK	Highly cytotoxic	Higher mitochondrial mass; relies on both glycolysis and OXPHOS	Particularly vulnerable to glucose deprivation and lactate accumulation; chronic activation leads to metabolic exhaustion (mitochondrial dysfunction, ROS accumulation, PD-1 upregulation)
	Activated NK	Rapid proliferation, produce IFN-γ, perforin, granzymes	High aerobic glycolysis and PPP; enhanced mitochondrial metabolism; NAD^+^ salvage pathway essential for OXPHOS	Glucose deprivation constrains glycolytic flux → insufficient ATP and impaired biosynthesis; lactate suppresses mTOR signaling and induces mitochondrial dysfunction
Myeloid cell	TAM			
	M1-TAM	Pro-inflammatory, antimicrobial, antitumor; produces ROS, RNS, TNF-α, IL-1β, IL-12	Rapid shift toward glycolysis and PPP	TME metabolic stress may impair M1 polarization and function
	M2-TAM	Tissue repair, angiogenesis, immune suppression; sustained energy metabolism	OXPHOS and FAO	Lactate promotes M2 differentiation ; PGE2 promotes M2 gene expression (ARG1, VEGF) via EP2/EP4 → cAMP-PKA
	DC			
	Immature DC	Maintain homeostasis, antigen uptake	Primarily relies on FAO	TME may prevent maturation, keeping DCs in an immature/tolerogenic state
	Conventional DC			
	cDC1	Cross-priming CD8^+^ T cells, antitumor immunity	Relies on OPA1 to maintain polarized mitochondria and intact ETC; sustains/enhances mitochondrial respiration (not glycolysis)	ETC impairment disrupts metabolic homeostasis → inhibits α-KG-dependent demethylases → DNA hypermethylation at PU.1/AP-1 sites → reduced immunogenicity
	cDC2	Moderate immune responses, tissue homeostasis	Lower reliance on mitochondrial respiration; maintains balance between glycolysis and OXPHOS without strong metabolic reprogramming	ETC perturbation has minimal impact on function; more resilient than cDC1
	Plasmacytoid DC	Maintain immune tolerance, regulate antiviral responses	Highly plastic; can be modulated via OXPHOS, FAO, and glycolysis; tolerogenic pDCs enhance OXPHOS/FAO	Lactate, Wnt5a-β-catenin-PPAR-γ, and microRNA-124 drive metabolic reprogramming, influencing immune polarization capacity
	Inflammatory DC	Immune activation	Early monocytes rely on glycolysis; differentiation/activation coordinately enhances mitochondrial metabolism, glycolysis, fatty acid, and glutamine pathways; CD1c^high^ favors glycolysis, CD86^high^ favors OXPHOS	TME may alter differentiation and metabolic programming of inf-DCs
	MDSC	Potent immunosuppression (suppress T-cell and NK cell function)	FAO; upregulation of glycolysis; downregulation of OXPHOS; lipid accumulation; heightened arginine and tryptophan depletion pathways	TME supports MDSC metabolic program; arginase-1 (depletes arginine) and iNOS (generates NO) suppress effector immune cells
	TAN	N2 subtype: Pro-tumorigenic: promotes angiogenesis, ECM remodeling, immune suppression	Similar to M2 macrophages (reliance on OXPHOS and FAO)	Differentiation and function influenced by tumor-derived lactate and PGE2

*TME* Tumor microenvironment, *Tn* Naïve T cell, *Teff* Effector T cell, *Tmem* Memory T cell, *Tex* Exhausted T cell, *Treg* Regulatory T cell, *Trm* Tissue-resident memory T cell, *Tfh* Follicular helper T cell, *γδ T* Gamma delta T cell, *MAIT* Mucosal-associated invariant T cell, *Bn* Naïve B cell, *Bgc* Germinal center B cell, *PC* Plasma cell, *Bmem* Memory B cell, *Breg* Regulatory B cell, *NK* Natural killer cell, *TAM* Tumor-associated macrophage, DC Dendritic cell, cDC1 Conventional type 1 dendritic cell, cDC2 Conventional type 2 dendritic cell, MDSC Myeloid-derived suppressor cell, *TAN* Tumor-associated neutrophil, *OXPHOS* Oxidative phosphorylation, *FAO* Fatty acid oxidation, *IFN-γ* Interferon-gamma, GZMB Granzyme B, HIF-1α Hypoxia-inducible factor 1-alpha, PD-1 Programmed cell death protein 1, TIM-3 T-cell immunoglobulin and mucin-domain containing-3, *LDHB* Lactate dehydrogenase B, *TCA* Tricarboxylic acid cycle, *TGF-β* Transforming growth factor-beta, *TIL* Tumor-infiltrating lymphocyte, *IL-17* Interleukin-17, *AhR* Aryl hydrocarbon receptor, *BCR* B cell receptor, *CD40* Cluster of differentiation 40, *AMPK* AMP-activated protein kinase, *TNF-α* Tumor necrosis factor-alpha, *ROS* Reactive oxygen species, *PPP* Pentose phosphate pathway, *NAD*^*+*^ Nicotinamide adenine dinucleotide, *mTOR* Mechanistic target of rapamycin, *RNS* Reactive nitrogen species, *PGE2* Prostaglandin E2, *ARG1* Arginase 1, *VEGF* Vascular endothelial growth factor, *EP2* E-prostanoid receptor 2, *EP4* E-prostanoid receptor 4, *cAMP* Cyclic adenosine monophosphate, *PKA* Protein kinase A, *OPA1* Optic atrophy 1, *ETC* Electron transport chain, *α-KG* Alpha-ketoglutarate, *PU.1* Purine-rich box 1, *AP-1* Activator protein 1, *PPAR-γ* Peroxisome proliferator-activated receptor gamma, *iNOS* Inducible nitric oxide synthase, *NO* Nitric oxide, *ECM* Extracellular matrix

##### Metabolic programs and functional suppression of T cells

T-cell function is highly dependent on finely coordinated metabolic programs,^133^ a feature that renders T cells particularly vulnerable to suppression under tumor-associated metabolic stress.^134,135^ The metabolic state of T cells is tightly coupled to their developmental and functional stages, and the regulation of specific metabolic pathways and transcription factors is central to determining their functional fate.^136^ Key T-cell subsets include CD8^+^ T cells, Tex cells, Tregs, Trm cells, Tfh cells, γδ T cells, and MAIT cells. Below, we summarize their metabolic programs and functional consequences.

**Naïve T cells (Tn)**: Under physiological conditions, naïve T cells remain metabolically quiescent, relying predominantly on OXPHOS with relatively low glycolysis and FAO to maintain homeostasis.^137^

**Effector T cells (Teff)**: Upon antigen stimulation, T-cell receptor (TCR) engagement together with CD28 costimulation rapidly activates the PI3K-AKT-mTOR axis and the transcription factor MYC. This drives a shift toward aerobic glycolysis and glutaminolysis to support clonal expansion and the production of effector molecules such as interferon-γ (IFN-γ) and granzyme B (GZMB).^138^ During this activation and effector phase, interleukin-2 (IL-2) signaling further reinforces mechanistic target of rapamycin complex 1 (mTORC1) activity through the JAK-STAT5 pathway, thereby sustaining high glycolytic flux.^139^ Meanwhile, hypoxic conditions within the TME stabilize HIF-1α, which can promote glycolysis; however, its persistent overexpression may also predispose T cells to a dysfunctional state.^97^

**Memory T cells (Tmem)**: As the immune response progresses, a subset of effector T cells differentiates into memory T cells. This transition depends on AMP-activated protein kinase (AMPK) activation, enhanced mitochondrial biogenesis, and metabolic reversion toward FAO, endowing memory T cells with long-term survival and self-renewal capacity.^140^

**Exhausted T cells (Tex)**: Within the TME, however, T cells are frequently driven into an exhausted or dysfunctional state. Metabolically, exhausted T cells exhibit mitochondrial impairment, reduced glycolytic capacity, and increased oxidative stress, accompanied by sustained high expression of inhibitory receptors such as PD-1.^141^ PD-1 signaling further suppresses the PI3K-AKT-mTOR pathway, creating a vicious cycle of metabolic insufficiency and functional decline.^142^ The TME disrupts CD8^+^ T-cell function through multiple metabolic mechanisms, thereby shaping an immunosuppressive landscape. Elevated lactate, a hallmark of highly glycolytic tumors, acidifies the extracellular milieu, directly inhibits mTORC1 activity,^143,144^ interferes with cytokine secretion, and disrupts intracellular pH homeostasis, collectively impairing CD8^+^ T-cell proliferation and cytotoxicity. Glucose deprivation arises from intense nutrient competition with tumor cells. Severe glucose scarcity suppresses the activity of key glycolytic enzymes such as hexokinase, precipitating an energetic crisis that not only constrains effector function but also activates energy-sensing kinases such as AMPK.^145^ This metabolic stress may further drive T cells toward a PD-1^high^ T-cell immunoglobulin and mucin-domain containing-3 (TIM-3)^high^ exhausted phenotype, potentially through epigenetic reprogramming.^146^ Tryptophan depletion, mediated by high expression of IDO1 or tryptophan 2,3-dioxygenase 2 (TDO2) in tumor or stromal cells, activates the stress-sensing kinase GCN2 in T cells, globally inhibiting protein translation.^147^ Concurrently, the tryptophan catabolite kynurenine acts as an endogenous ligand for AhR, directly suppressing CD8^+^ T-cell proliferation, promoting apoptosis, and inhibiting the production of effector cytokines such as IFN-γ.^148^

**Regulatory T cells (Tregs)**: In stark contrast, Treg cells display remarkable adaptability to the tumor metabolic microenvironment, allowing them to survive and maintain suppressive function. The intrinsic metabolic program of Tregs favors OXPHOS and FAO, rendering them less dependent on glucose and more tolerant of glucose-deprived conditions.^149^ Studies have shown that Tregs can efficiently uptake and utilize lactate from the microenvironment, converting it to pyruvate via lactate dehydrogenase B (LDHB) and fueling the TCA cycle as an alternative energy source.^150^ Moreover, Tregs respond differently to AhR signaling compared with effector T cells; certain AhR ligands, potentially including tumor-derived metabolites, can promote Treg differentiation and function.^151,152^ Consequently, under the same metabolic pressures that suppress CD8^+^ T cells, such as low glucose, high lactate, and elevated kynurenine, Treg survival and immunosuppressive activity are preserved or even enhanced, further exacerbating local immune suppression within tumors.

**Tissue-resident memory T cells (Trm)**: Tissue-resident memory CD8^+^ T cells achieve long-term survival and functional maintenance through metabolic reprogramming within specific tissue environments. Trm cells exhibit metabolic features distinct from those of circulating Tmem cells, including enhanced FAO, active mevalonate-cholesterol synthesis, and mitochondrial respiratory adaptability. The key transcription factor sterol regulatory element-binding protein 2 (SREBP2) regulates mevalonate-cholesterol metabolism and coenzyme Q (CoQ) production, supporting mitochondrial function and energy homeostasis. Tissue-specific signals such as transforming growth factor-beta (TGF-β) help maintain the Trm metabolic state and residency. In tumors, Trm cells use similar metabolic programs to promote tumor-infiltrating lymphocyte (TIL) accumulation and antitumor immunity, enhancing immune control and the efficacy of immunotherapy.^153^

**Follicular helper T cells (Tfh)**: Tfh cells are key regulators of humoral immunity, residing in germinal centers (GCs) to assist B cells in somatic hypermutation and class-switch recombination, generating high-affinity, long-lived antibodies. Tfh differentiation and effector function rely on extensive metabolic reprogramming, characterized by enhanced glycolysis and lipid metabolism. mTORC1 and mTORC2 signaling drive these anabolic pathways, while glucose transporter 1 (GLUT1)-mediated glucose uptake provides energy and biosynthetic precursors, supporting GC responses and effective humoral immunity.^154^

**Unconventional T-cell subsets**: γδ T cells and MAIT cells are unconventional T-cell subsets whose effector functions are tightly regulated by intrinsic metabolic programs and the TME. γδ T cells exhibit subset-specific metabolic features: IFN-γ-producing γδ T cells primarily rely on glycolysis, whereas IL-17-producing γδ T cells depend on mitochondrial oxidative metabolism. These metabolic programs are established during thymic development under TCR and transcription factor regulation and persist in peripheral tissues and the TME. High glucose enhances IFN-γ^+^ γδ T-cell proliferation and antitumor activity, whereas high-lipid environments selectively expand IL-17^+^ γδ T cells, promoting tumor growth. Metabolic interventions can selectively modulate γδ T-cell subset functions, representing a “thymic metabolic programming-TME adaptation-immune function regulation” mechanism.^155^ MAIT cells, enriched in the liver and mucosal tissues, can recognize microbial metabolites and rapidly produce cytokines such as IFN-γ and IL-17. In hepatocellular carcinoma (HCC), CD4^+^ MAIT cells polarize toward a Th17-like phenotype and are the primary source of IL-17A. Their metabolism is characterized by enhanced glycolysis, which is further promoted by tumor-derived tryptophan metabolites via the AhR pathway. Glycolysis supports IL-17A production through posttranscriptional regulation, while this metabolic crosstalk enhances tumor lipid storage and proliferation, forming a protumor feedback loop.^156^

##### B-cell metabolism and functional potential

Humoral immunity mediated by B cells is likewise highly dependent on metabolic reprogramming and is therefore vulnerable to dysfunction within the tumor metabolic microenvironment.^157^ B-cell activation, differentiation, and effector functions rely on distinct metabolic programs to meet stage-specific bioenergetic and biosynthetic demands. Key B-cell subsets include Bn cells, Bgc cells, PCs, Bmem cells, and Bregs. Below, we summarize their metabolic programs and functional consequences.

**Naïve B cells (Bn)**: Under steady-state conditions, naïve B cells maintain homeostasis with relatively low glycolysis and OXPHOS, remaining metabolically quiescent.

**Germinal center B cells (Bgc)**: During GC reactions in secondary lymphoid organs, B cells undergo clonal expansion, immunoglobulin class switching, and somatic hypermutation, processes that require exceptionally high biosynthetic activity.^158^ These events are jointly driven by B-cell receptor (BCR) and CD40 signaling, thereby switching cellular metabolism toward a high-flux state dominated by aerobic glycolysis and glutaminolysis.^159^ This anabolic configuration supplies abundant precursors for nucleotide and lipid synthesis while fueling DNA repair mechanisms required for somatic hypermutation.

**Plasma cells (PCs)**: Following affinity-based selection, B cells differentiate into plasma cells or memory B cells, accompanied by divergent metabolic programs. Antibody production is primarily executed by short-lived and long-lived plasma cells, whose metabolic programs are centered on supporting large-scale immunoglobulin synthesis and secretion. These cells maintain active glycolysis and rely heavily on the biosynthetic capacity of the endoplasmic reticulum and mitochondria.^160^ Mitochondrial OXPHOS not only provides energy but also contributes to proper disulfide bond formation in antibodies.^161^

**Memory B cells (Bmem)**: In contrast, memory B cells revert to a more energy-efficient and durable metabolic state characterized by enhanced fatty acid oxidation and OXPHOS, supported by AMPK signaling to ensure long-term survival and rapid recall responses.

**Regulatory B cells (Bregs)**: A subset of B cells differentiates into Bregs with immunosuppressive functions. Emerging evidence suggests that their suppressive activity, such as IL-10 production, may depend on specific metabolic programs, including a preference for oxidative phosphorylation, potentially conferring greater adaptability under metabolic stress; however, the precise regulatory mechanisms remain incompletely understood.^162,163^ Within the TME, metabolic stress profoundly interferes with productive B-cell differentiation and function.^164^ Glucose limitation directly suppresses glycolytic flux in germinal center B cells and plasmablasts, thereby inhibiting proliferation and antibody synthesis.^165^ Lactate accumulation and the resulting acidic milieu may further disrupt B-cell signaling and survival.^166^ Furthermore, effector B cells in the TME activate glycolysis through BCR, Toll-like receptor (TLR) signaling, and B-cell activating factor (BAFF), which drives antibody production.^167,168^ The Fc portion of the antibodies then binds to FcγRs on tumor-associated macrophages, mediating antibody-dependent cellular phagocytosis (ADCP) and exerting cytotoxic effects.^169^ Together, these factors impair the generation and affinity maturation of tumor-specific antibodies while potentially favoring the expansion or functional dominance of metabolically adaptable regulatory B-cell subsets, indirectly reinforcing immunosuppression. Thus, tumor metabolic reprogramming not only suppresses cellular immunity but also systemically impairs the humoral immune axis.

##### Metabolic vulnerability of natural killer cells

NK cells, as central cytotoxic effectors of the innate immune system, are highly dependent on dynamic metabolic programs for activation, proliferation, and effector function.^170,171^ Within the TME, NK cells are particularly metabolically vulnerable due to nutrient deprivation, hypoxia, and the accumulation of suppressive metabolites. Key NK cell metabolic pathways and checkpoints tightly regulate their cytotoxic potential and survival.

**Resting NK cells**: In a resting state, NK cells primarily rely on OXPHOS to maintain basal homeostasis and survival.^172^ Glycolytic and anabolic pathways are relatively quiescent, consistent with low energy demands. Human NK cells in peripheral blood are primarily divided into CD56^bright^ and CD56^dim^ subsets. CD56^dim^ NK cells are highly cytotoxic and rely on both glycolysis and OXPHOS with higher mitochondrial mass, whereas CD56^bright^ NK cells mainly produce cytokines such as IFN-γ and TNF-α with lower metabolic activity.^173^

**Activated NK cells**: Upon activation by cytokines such as IL-2, IL-12, or IL-15, NK cells rapidly initiate metabolic reprogramming, markedly upregulating aerobic glycolysis and the PPP. This transition provides the biosynthetic precursors and immediate energy required for rapid proliferation and the production of effector molecules, including IFN-γ, perforin, and granzymes.^174^ Concurrently, mitochondrial metabolism is enhanced to support sustained effector function. It is worth noting that the NAD^+^ salvage pathway is essential for maintaining mitochondrial homeostasis and OXPHOS activity, which in turn supports critical NK cell effector functions.^175,176^ In addition, transcription factors such as MYC and HIF-1α play key roles in this metabolic reprogramming, among which HIF-1α is particularly stabilized in hypoxic microenvironments and can further enhance glycolysis.^97^ NK cell activity is tightly regulated by intrinsic metabolic checkpoints. Activation-induced mTORC1 activity is essential for NK cell functional maturation, and its inhibition results in severe defects in IFN-γ production and cytotoxicity.^177^ AMPK functions as a key energy and stress sensor to maintain metabolic balance,^178,179^ while deacetylases such as sirtuin 1 (SIRT1) modulate mitochondrial function and metabolism, thereby influencing NK cell survival and sustained effector potential, which are critical for long-term antitumor immunity.^180^ Together, these metabolic checkpoints form an intrinsic network that regulates the magnitude and duration of NK cell responses. Similar to CD8^+^ T cells, NK cells face substantial metabolic vulnerability within the TME. Glucose deprivation directly constrains glycolytic flux, leading to insufficient ATP generation and impaired biosynthesis, rapidly compromising cytotoxic activity and proliferative capacity. Moreover, excessive lactate produced by tumor glycolysis acidifies the microenvironment, suppresses mTOR signaling, interferes with cytokine production and secretion, and may induce mitochondrial dysfunction, collectively inhibiting NK cell effector functions. Persistent activation signals, such as high expression of stress ligands (e.g., CD48, MHC class I polypeptide-related sequence A/B [MICA/B]) on tumor cells,^181^ can further drive NK cells into a state of chronic activation, ultimately leading to metabolic exhaustion characterized by mitochondrial dysfunction, reduced glycolytic capacity, ROS accumulation, and upregulation of inhibitory receptors such as PD-1. These converging metabolic challenges, including nutrient competition, accumulation of suppressive metabolites, and chronic activation, render NK cells, such as CD8^+^ T cells, unable to sustain effective antitumor immunity, thereby weakening innate immune surveillance and compromising antitumor function.

##### Metabolic remodeling and phenotypic polarization of myeloid cells

Due to their pronounced metabolic and functional plasticity, myeloid cells are particularly susceptible to reprogramming into immunosuppressive phenotypes within the tumor metabolic microenvironment. As one of the most abundant and versatile immune populations in tumors, the metabolic state of myeloid cells acts as a decisive regulatory node that governs their functional polarization toward either proinflammatory or immunosuppressive phenotypes.^182^

**Tumor-associated macrophages (TAMs)**: Macrophages represent a canonical example of the tight coupling between metabolism and function, a connection that is particularly evident in the distinct metabolic programs underlying M1/M2 polarization.

***M1-TAMs***: Under classical activation signals such as IFN-γ and lipopolysaccharide (LPS), macrophages differentiate into the M1 phenotype, characterized by a rapid metabolic shift toward glycolysis and the pentose phosphate pathway.^183^ This glycolytic burst provides rapid energy, generates antimicrobial ROS and reactive nitrogen species (RNS), and supports the synthesis of proinflammatory cytokines such as TNF-α, IL-1β, and IL-12.^184^

***M2-TAMs***: Alternative activation by IL-4 or IL-13 induces M2 macrophage polarization, which relies on oxidative phosphorylation and fatty acid oxidation to meet the sustained energy demands of tissue repair, angiogenesis, and immune regulation.^185,186^ Tumor-derived metabolites actively shape this polarization process: lactate promotes M2 differentiation by stabilizing HIF-1α, directly inhibiting histone deacetylases (HDACs), and activating G protein-coupled receptors such as GPR81.^187^ PGE2, through prostaglandin E2 receptor-2 (EP2) and EP4 receptor signaling and activation of the cAMP-PKA pathway, further promotes the expression of M2-associated genes such as arginase 1 (ARG1) and vascular endothelial growth factor (VEGF).^188^ Consequently, tumor glycolytic and lipid metabolic products directly contribute to the construction of an immunosuppressive TME.

**Dendritic cells (DCs)**: DC metabolism is closely linked to their maturation status and functional capacity.^189^ Immature DCs primarily rely on FAO to maintain homeostasis and antigen uptake.^190^ Upon receiving maturation signals such as Toll-like receptor agonists, DCs undergo metabolic reprogramming toward glycolysis,^191^ which provides the energy and biosynthetic precursors required for migration to lymph nodes, upregulation of costimulatory molecules (CD80/CD86), and production of IL-12.^31,192,193^ However, within the TME, excessive lipids, particularly oxidized lipids released or generated by tumor cells, are avidly taken up by DCs.^194^ Intracellular lipid accumulation induces severe endoplasmic reticulum (ER) stress and mitochondrial dysfunction.^195^ ER stress suppresses DC maturation and proinflammatory cytokine production through unfolded protein response pathways, while mitochondrial damage compromises bioenergetic capacity. As a result, DCs acquire a tolerogenic phenotype with impaired antigen presentation, failing to effectively prime naïve T cells and potentially inducing T-cell dysfunction.

***Conventional type 1 DCs (cDC1)***: cDC1s are critical for antitumor immunity through cross-priming CD8^+^ T cells. cDC1s rely on OPA1 (optic atrophy 1 protein, a mitochondrial dynamin-like GTPase) to maintain polarized mitochondria and intact ETC, supporting nuclear respiratory factor 1 (NRF1) activity, NAD^+^/NADH balance, and antigen presentation.^196^ Upon pattern recognition receptor (PRR) stimulation, cDC1s sustain or enhance mitochondrial respiration without switching predominantly to glycolysis or lactate fermentation, promoting costimulatory molecule expression, migration, and effective CD4^+^ and CD8^+^ T-cell activation. ETC impairment disrupts metabolic homeostasis, inhibits α-ketoglutarate (α-KG)-dependent demethylases, and causes DNA hypermethylation at purine-rich box 1 (PU.1) and activator protein 1 (AP-1) binding sites, reducing immediate early gene expression and immunogenicity.^197^ This mechanism highlights how mitochondrial metabolism enables cDC1s to maintain a primed transcriptional state for rapid, robust immune responses and antitumor activity.

***Conventional type 2 DCs (cDC2)***: cDC2s exhibit a lower reliance on mitochondrial respiration and do not significantly enhance oxidative phosphorylation upon activation. After PRR stimulation, cDC2 cells primarily depend on basal metabolism for energy, maintaining a balance between glycolysis and mitochondrial respiration without undergoing strong metabolic reprogramming. ETC perturbation has minimal impact on costimulatory molecule expression and T-cell activation, reflecting weak dependence on the electron transport chain. cDC2 cells show limited changes in transcriptional regulation and DNA methylation, preserving a stable functional state. Compared with cDC1s, cDC2s have lower metabolic plasticity but more resilient functionality, making them suitable for maintaining moderate immune responses and tissue homeostasis.^197^

***Plasmacytoid DCs (pDCs)***: pDCs play a key role in maintaining immune tolerance and regulating antiviral responses. Their metabolic state is highly plastic and can be modulated via OXPHOS, FAO, and glycolysis. Tolerogenic pDCs tend to enhance OXPHOS and FAO to maintain ATP levels and redox balance while suppressing proinflammatory cytokine production. Environmental signals such as lactate, Wnt5a-β-catenin-PPAR-γ, and microRNA-124 can drive metabolic reprogramming, thereby influencing the immune polarization capacity of pDCs.^198^

***Inflammatory DCs (inf-DCs)***: Inf-DCs undergo metabolic reprogramming during differentiation and maturation. Early monocytes primarily rely on glycolysis, while mitochondrial metabolism, glycolysis, fatty acid, and glutamine pathways are coordinately enhanced during differentiation and activation, providing energy for immune activation. Different DC subsets display metabolic preferences: CD1c^high^ favors glycolysis, whereas CD86^high^ favors mitochondrial respiration (OXPHOS).^199^ These metabolic features are closely associated with their immune phenotypes.

**Myeloid-derived suppressor cells (MDSCs)**: MDSCs constitute a heterogeneous population with potent immunosuppressive capacity.^200^ The maintenance of their function is highly dependent on FAO, upregulation of glycolysis, and downregulation of OXPHOS. It also exhibits lipid accumulation and heightened activity of pathways that deplete crucial extracellular nutrients, such as arginine and tryptophan.^201^ Specifically, these metabolic shifts support the production of key immunosuppressive mediators: ARG1, which depletes arginine, and iNOS, which generates nitric oxide, collectively suppressing T-cell and NK cell function.^202^

**Tumor-associated neutrophils (TANs)**: TANs also exhibit functional heterogeneity.^203^ The protumorigenic N2 subtype may display metabolic features similar to those of M2 macrophages, such as reliance on fatty acid oxidation, and its differentiation and function are likely influenced by tumor-derived metabolites, including lactate and PGE2.^204,205^ Through these mechanisms, N2 neutrophils contribute to angiogenesis, extracellular matrix remodeling, and immune suppression. Collectively, tumors systematically shape a metabolic environment that directs myeloid cells toward immunosuppressive and protumor phenotypes, representing a critical mechanism of immune evasion.

Collectively, tumor metabolic reprogramming creates a nutrient-deprived microenvironment enriched in suppressive metabolites, exerting systemic inhibitory effects on antitumor immune responses. On the one hand, tumor cells outcompete immune cells for key metabolic substrates such as glucose, amino acids, and lipids, directly impairing the metabolic capacity and cytotoxic function of effector T cells and NK cells. On the other hand, the accumulation of metabolites such as lactate, kynurenine, and prostaglandins provides favorable conditions for the survival and functional maintenance of immunosuppressive populations, including regulatory T cells and MDSCs. The metabolic vulnerability of effector immune cells stands in stark contrast to the high adaptability of immunosuppressive cells to the tumor metabolic environment, together forming a critical metabolic barrier to effective antitumor immunity. Importantly, tumor metabolic reprogramming does not exclusively promote immune suppression. Under certain conditions, metabolic stress may induce immunogenic cell death, release danger-associated molecular patterns such as ATP, or force tumor cells to upregulate immunotherapeutic targets such as PD-L1, thereby exposing exploitable metabolic vulnerabilities. Therefore, tumor metabolic reprogramming represents both a fundamental basis of immune evasion and a promising therapeutic entry point. Systematic dissection of the metabolic dependencies and adaptive mechanisms of distinct immune cell subsets using single-cell multiomics will provide a theoretical foundation for rational combinations of metabolic interventions and immunotherapies, potentially overcoming the current limitations in cancer immunotherapy efficacy.

## Crosstalk between metabolism and immunity in the TME

The TME is a dynamic and evolving selective ecosystem. Its core characteristic lies in the intense competition between tumor cells and infiltrating immune cells for limited metabolic resources, as well as their bidirectional dialog mediated by metabolic signaling,^206^ which forms the foundation of the regulatory interplay between metabolic products and the immune microenvironment.^207,208^ This interaction not only determines the ultimate fate of the immune response but also drives tumor immunoediting and evolution. It is not a simple mutual inhibition but a multilayered, bidirectional, and networked regulatory system that spans the entire process from nutrient competition to signal transduction. This interplay constitutes the very theoretical framework upon which the concept of “metabolic immune checkpoints (MICs)” is built.

### Nutrient competition: the systemic deprivation of immune cell function

Tumor cells, driven by their uncontrolled proliferative nature, act as dominant metabolic competitors within the TME, fundamentally undermining the functional basis of immune cells by consuming essential nutrient substrates. This competition is active and selective. Glucose deprivation is one of the most prominent mechanisms. The high glycolytic flux of tumor cells (the Warburg effect) makes them a black hole for glucose, leading to a significant reduction in extracellular glucose concentration.^33–35^ This represents a fatal blow for effector T cells and NK cells, which are highly dependent on aerobic glycolysis to support their activation, proliferation, and effector functions, including IFN-γ and perforin production.^174^ Glucose scarcity directly leads to a decrease in intracellular glycolytic intermediates, inhibits mTORC1 signaling pathway activity,^144^ and consequently weakens protein synthesis and cellular metabolic reprogramming capacity. More profoundly, long-term glucose restriction can drive T cells toward an epigenetically locked, functionally exhausted state by altering histone modifications and chromatin accessibility.^209^ This allows the tumor not only to suppress the immediate function of immune cells but also to program their long-term non-responsive fate.

Glutamine deprivation targets another critical metabolic node. Glutamine is not only a substrate for protein synthesis but also an essential carbon and nitrogen source for TCA cycle anaplerosis and de novo nucleotide synthesis,^210^ which are vital for T-cell and macrophage activation. Tumor cells engage in direct competition with immune cells by highly expressing glutamine transporters (e.g., ASCT2) and the key catabolic enzyme glutaminase (GLS), thereby significantly enhancing their uptake and utilization efficiency of glutamine. Glutamine scarcity limits the biosynthetic capacity of activated T cells, inhibits their clonal expansion, and impairs the proinflammatory (M1-type) polarization of macrophages.^211^ Deprivation of essential amino acids is more precise and carries signaling significance, primarily manifested in the metabolism of tryptophan and arginine. The high expression of IDO1 and ARG1 by tumor cells or tumor-associated MDSCs is central to this process.^212^ IDO1 catalyzes the breakdown of tryptophan along the kynurenine pathway, causing local tryptophan depletion. Low tryptophan levels activate the GCN2 kinase within T cells, triggering the integrated stress response and globally inhibiting protein translation, thereby arresting cell cycle progression.^213,214^ Simultaneously, ARG1 hydrolyzes arginine, not only depriving T cells of the substrate needed for proliferation but also directly causing downregulation of the T-cell receptor CD3ζ chain, impairing the integrity of TCR signal transduction and rendering T cells anergic even in the presence of antigen.^215^ This dual deprivation strategy dismantles the T-cell response potential at both the biosynthetic capacity and signal reception levels.

### Metabolic waste products as immunomodulatory signals: from toxicity to active regulation

Tumor metabolism not only consumes resources but also converts its copious waste products into potent immunomodulatory signaling molecules at specific concentrations.^216^ This represents another sophisticated strategy for immune evasion through metabolic reprogramming and exemplifies how metabolic products serve as active immunomodulators regulating immune cell metabolism within the TME.^217^

Lactate, as the end-product of glycolysis, plays a role far beyond that of a metabolic waste product. Its impact on immune function exhibits clear concentration-, cell type-, and microenvironment-dependent characteristics. High concentrations of lactate are a primary cause of TME acidosis (decreased pH). The acidic extracellular environment can directly interfere with T-cell receptor signal transduction^218^ and vesicular transport required for cytokine secretion.^219^ More importantly, lactate can enter immune cells via monocarboxylate transporters (e.g., MCT1), directly inhibiting the cytotoxic activity of cytotoxic T lymphocytes and NK cells and reducing IFN-γ production.^220^ For example, in non-small cell lung cancer (NSCLC), lactate can serve as a carbon source for the TCA cycle, thereby reshaping immune cell energy metabolism.^221^ Furthermore, lactate acts as a signaling molecule that, through its receptor GPR81, directly induces the polarization of tumor-associated macrophages toward a proangiogenic, tissue-repairing, and immunosuppressive M2 phenotype,^129^ thereby reshaping the entire immune microenvironment. The effects of lactate on T-cell functions are microenvironment dependent: its immune-suppressive effect is more pronounced under hypoxic conditions in the TME, whereas in normoxic conditions with relatively intact T-cell function, lactate can be metabolized through the TCA cycle. Notably, moderate concentrations of lactate (1.5-3 mM) can enhance the stem-like characteristics and memory formation of CD8^+^ T cells,^222^ while higher concentrations (10-30 mM) directly inhibit their effector functions.^223^ Simultaneously, lactate accumulation promotes preferential uptake by Tregs, which utilize it through mitochondrial oxidative metabolism pathways, thereby functionally suppressing antitumor immune responses.

Kynurenine, a key intermediate in IDO1-mediated tryptophan metabolism, facilitates the transition from nutrient deprivation to direct immunosuppressive signaling.^213^ As an endogenous ligand for AhR, kynurenine enters the T-cell nucleus and binds to AhR, forming a transcriptional complex. This complex not only directly inhibits effector T-cell proliferation and promotes their apoptosis but also positively regulates transcription factors such as Foxp3, thereby actively inducing regulatory T-cell differentiation.^224^ Thus, the IDO1-Kyn-AhR axis establishes a self-reinforcing immunosuppressive loop: depleting tryptophan suppresses effector T-cell function while generating kynurenine to further inhibit effector T cells and promote immunosuppressive Tregs.

Adenosine production exemplifies the tumor’s ability to hijack and transform extracellular danger signals and serves as an important nucleotide metabolite regulating immune cell metabolism within the TME.^217^ In the hypoxic or inflammatory TME, extracellular ATP levels rise (often from dying or stressed cells).^225^ The ectoenzymes CD39 (hydrolyzing ATP/ADP to AMP) and CD73 (hydrolyzing AMP to adenosine), which are highly expressed by tumor cells and certain stromal cells, convert this danger signal indicative of tissue damage and immune activation (ATP) into a potent immunosuppressive signal—adenosine.^226^ Adenosine binds to the A2A receptor on immune cells, activating the intracellular cAMP-PKA signaling pathway and strongly inhibiting the activation, proliferation, cytotoxicity, and production of key cytokines (e.g., IFN-γ, TNF-α) in T cells and NK cells.^227^ The CD39/CD73-adenosine axis thus becomes a critical immune brake node within the TME.

### Harsh physicochemical environment: terminal stress on immune responses

The combined effects of nutrient competition and metabolic waste accumulation ultimately shape the harsh physicochemical properties of the TME, collectively suppressing and distorting immune cell function. Acidosis (low pH environment) results from the combined effects of lactate and CO_2_ produced by respiration.^129^ Beyond the specific signals mediated by lactate, the overall low pH environment itself can impair the normal physiological functions of most immune cells, including migration, phagocytosis, and signal transduction.^228^ Notably, this acidic environment may conversely enhance the suppressive function of regulatory T cells^229^ and affect the effector functions of the antibody and complement systems,^230^ thereby exerting suppression at both the innate and adaptive immunity levels.

Hypoxia is an inevitable consequence of the disorganized structure and dysfunctional nature of the tumor vasculature, often exhibiting regional oxygen partial pressure < 10 mmHg.^231^ Hypoxia reprograms cellular metabolism by stabilizing the transcription factor HIF-1α.^232^ In tumor cells, HIF-1α drives the expression of glycolysis and angiogenesis-related genes.^233^ In immune cells, its role is more complex and context dependent: in MDSCs and certain macrophage subsets, HIF-1α can promote their immunosuppressive functions; in cytotoxic T cells, chronic hypoxia may lead to their dysfunction.^234^ Although HIF-1α might transiently enhance the killing capacity of NK cells under certain conditions,^235^ within the long-term, chronic hypoxic TME of solid tumors, its net effect is typically to promote immunosuppressive cell functions and impair effector cell functions. Oxidative stress arises from the abnormally active metabolic activity of tumor cells, leading to excessive production of reactive oxygen species, such as superoxide and hydrogen peroxide.^231^ Physiological levels of ROS are part of T-cell activation signaling; however, sustained, high levels of ROS in the TME cause mitochondrial dysfunction, oxidative DNA damage, and lipid peroxidation within immune cells.^236^ This oxidative stress accelerates the functional exhaustion of T cells and NK cells and may induce their apoptosis, further undermining the persistence of the antitumor immune response.

In summary, tumor metabolic reprogramming, a hallmark of cancer, not only fuels rapid proliferation^237,238^ but also establishes functional checkpoints at the metabolism-immune interface, driving immune evasion.^11^ From this complex metabolic-immune crosstalk, we distill its central regulatory nodes into the concept of “metabolic immune checkpoints (MICs).” MICs are biomolecules essential to core metabolism (e.g., nutrient uptake, catabolism, waste generation) whose activity or expression directly and specifically regulates immune cell function. Recent studies have revealed synergistic networks between metabolic enzymes (e.g., LDHA and ACLY) and immune checkpoints,^124,239^ suggesting promising combination strategies. However, key challenges persist: mechanisms linking specific metabolites to immune function remain unclear, clinical translation of metabolic-immune drugs has been inconsistent,^18^ and most studies fail to integrate the dynamic “enzyme-metabolite-immune receptor” axis. To bridge these gaps, we propose the MIC framework. By systematically mapping this regulatory network, we aim to provide a theoretical foundation for rational combination therapies and accelerate their clinical translation.

## Categorization, mechanisms, and regulatory network of “metabolic immune checkpoints”

### Proposal of the MIC concept and its differences from traditional checkpoints

With the deepening of research on tumor immunotherapy, traditional immune checkpoints (such as PD-1/PD-L1, cytotoxic T-lymphocyte-associated protein 4 [CTLA-4], T-cell immunoreceptor with Ig and ITIM domains [TIGIT], lymphocyte-activation gene 3 [LAG-3], and TIM-3) are no longer sufficient to fully explain the complex interactions between metabolism and immunity in the TME.^240^ These classical molecules mainly reflect direct communication between immune cells while overlooking the local environment and the physical, chemical, and nutritional ecosystem shaped by metabolic reprogramming. Based on this, we have innovatively proposed a classification framework for MICs, expanding the research perspective from surface checkpoint signals on cells to environmental checkpoints that regulate the entire immune microenvironment. Here, we define MICs as metabolism-derived regulatory hubs within the tumor microenvironment that actively and directly constrain antitumor immune responses. The core characteristics of MICs include (1) metabolic dependence: their functions are directly driven by metabolic enzyme activity, metabolite accumulation, or energy status; (2) immune regulatory directness: they themselves or their products can directly intervene in immune cell function, differentiation, or survival through clear molecular mechanisms; and (3) targetability: they have clear molecular structures and pharmacological intervention windows.

**Fig. 1:**
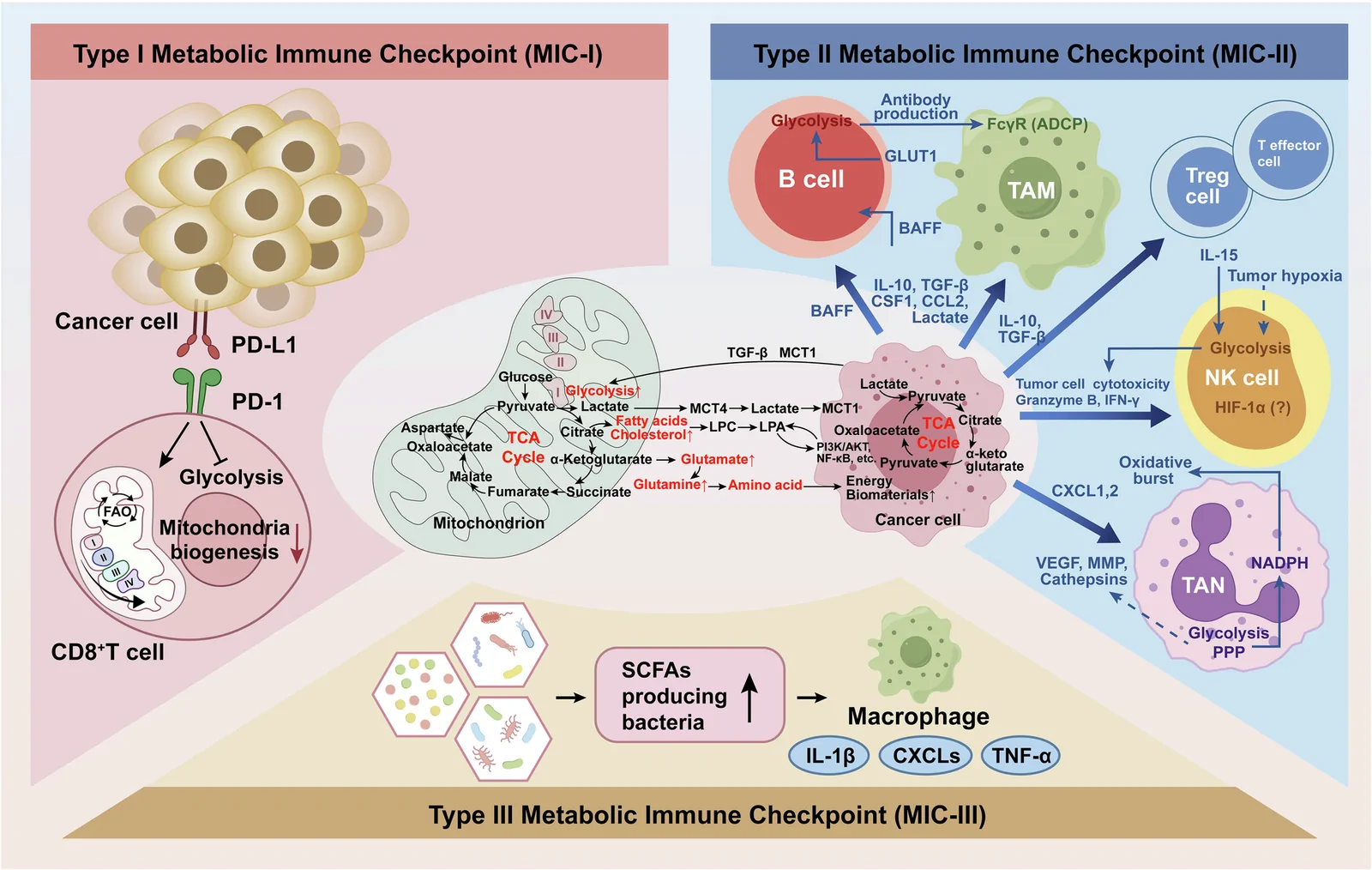
Tumor Metabolism-Immune Regulatory Network: A Three-Dimensional MIC Framework. MICs are categorized into three functional types. MIC-I involves metabolic modifications of classical immune checkpoint pathways, in which metabolites or metabolic enzymes regulate checkpoint signaling through posttranslational or functional mechanisms. MIC-II refers to metabolic enzyme-dependent immune nodes, where metabolic enzymes and their products act as regulatory hubs to directly modulate immune cell function. MIC-III describes microbiome-metabolism-immune interaction networks, in which gut microbiota-derived metabolites shape antitumor immunity. MIC: Metabolic immune checkpoint; PD-L1: Programmed death-ligand 1; PD-1: Programmed cell death protein 1; TCA cycle: Tricarboxylic acid cycle; GLUT1: Glucose transporter type 1; BAFF: B-cell activating factor; Treg: Regulatory T cell; CSF1: Colony stimulating factor 1; CCL2: C-C motif chemokine ligand 2; TGF-β: Transforming growth factor-beta; IFN-γ: Interferon-gamma; CXCLs: C-X-C motif chemokine ligands; HIF-1α: Hypoxia-inducible factor 1-alpha; NK cell: Natural killer cell; TAM: Tumor-associated macrophage; TAN: Tumor-associated neutrophil; NADPH: Nicotinamide adenine dinucleotide phosphate; PPP: Pentose phosphate pathway; VEGF: Vascular endothelial growth factor; MMP: Matrix metalloproteinase; MCT1: Monocarboxylate transporter 1; MCT4: Monocarboxylate transporter 4; LPC: Lysophosphatidylcholine; LPA: Lysophosphatidic acid; SCFAs: Short-chain fatty acids; IL-1β: Interleukin-1 beta; TNF: Tumor necrosis factor

The proposal of this concept is an important extension and complement to the classical checkpoint paradigm: from the content of the conversation to the environment of the conversation. Traditional checkpoints act as stop signals (ligand‒receptor interactions) between cells, while MICs create the overall environment required for signal transmission. For example, lactate (a key MIC) does not directly bind to the T-cell receptor, but it acidifies the microenvironment, inhibits metabolic enzyme activity, and induces PD-L1 modification,^17^ thereby creating conditions that enhance the effectiveness of stop signals such as PD-1/PD-L1. From dedicated switches to infrastructure regulation: PD-1 and others are dedicated brakes in the immune system, while MICs such as IDO1 and CD73 essentially maintain infrastructure for basic cell metabolism (e.g., amino acid metabolism, purine metabolism).^226,241^ Tumors hijack these infrastructures, not only meeting their own growth needs but also turning them into multieffect valves to suppress immune responses in a more fundamental and widespread manner. From single targets to network targets: Targeting traditional checkpoints typically blocks a single pathway, whereas MICs are embedded in interconnected metabolic networks. Targeting a single MIC (e.g., LDHA) can simultaneously improve nutrient competition, reduce inhibitory metabolite accumulation, and influence checkpoint modifications,^239,242^ thereby generating synergistic immune recovery effects (Table 2, Fig. 1).

**Table 2:** Systematic classification of tumor metabolic immune checkpoints (MICs)

“MICs” Category	Name	Description	Representative Example
Type I (MIC-I)	Metabolic modification-type immune checkpoints	Posttranslational modification or functional regulation of classical immune checkpoint pathways by metabolites or enzymes	Lactate enhances PD-L1 stability through lactylation^17^
Type II (MIC-II)	Metabolic enzyme-dependent immune nodes	Metabolic enzymes and their catalytic products act as direct regulatory hubs to influence immune cell function	LDHA inhibits T-cell function through lactate accumulation^239,242^
Type III (MIC-III)	Microbiome-metabolism-immune interaction network	Gut microbiota-derived metabolites interact with the host immune system to shape antitumor immunity	SCFA butyrate produced by gut microbiota regulates Treg differentiation, leading to tumor immune tolerance^19,305^

*MIC* Metabolic immune checkpoint, *PD-L1* Programmed death-ligand 1, *LDHA* Lactate dehydrogenase A, *SCFA* Short-chain fatty acid, *Treg* Regulatory T cell

The fundamental difference between MICs and traditional immune checkpoints is that traditional checkpoints are endogenous, evolutionarily conserved coinhibitory signaling molecules in the immune system, primarily serving to maintain self-tolerance and prevent excessive activation. In contrast, MICs are essentially metabolic enzymes, transport proteins, or metabolite receptors, whose immune regulatory functions are new properties derived from their original metabolic functions under evolutionary pressure from tumors. This fundamental distinction determines differences in targeting strategies: traditional checkpoints typically use antibodies to block protein interactions, whereas MICs require small molecule inhibitors (SMIs) to interfere with enzyme activity, inhibit transport functions, or antagonize receptor signals (Table 3). Defining metabolic interactions as checkpoints has significant theoretical and clinical value. It provides a unified framework for systematically understanding how tumors utilize seemingly disparate metabolic events, such as glucose deprivation, lactate accumulation, and tryptophan degradation, to jointly construct an immune suppression barrier. More importantly, it elevates these metabolic nodes as potential therapeutic targets, and their targetability characteristics directly suggest new directions for drug development. By using SMIs, metabolic modulators, and other interventions to target these nodes, it may be possible to reverse the immune suppression status of the TME, thereby overcoming metabolic-mediated resistance to immunotherapy and providing new ideas for converting cold tumors and designing combination therapy strategies.

**Table 3:** Comparison between MICs and classical immune checkpoints

Feature Dimension	Classical Immune Checkpoints	Metabolic Immune Checkpoints (MICs)
Nature	Endogenous signal receptors/ligands of the immune system	Metabolic enzymes, transporters, metabolites, or metabolic sensors
Core Function	Maintain self-immune tolerance and prevent overactivation	Maintain basic metabolic flux of tumor or immune cells; their immune regulatory function is a derived property
Regulatory Logic	Intercellular protein‒protein interactions	Alter local chemical environment (metabolite concentration, pH) or provide small molecule ligands
Regulation Levels	Mainly at the posttranscriptional (protein expression) and signal transduction levels	From metabolic flux, posttranslational modifications to epigenetic and transcriptional regulation across multiple levels
Inducibility	Typically induced by T-cell activation signals	Mainly induced by TME metabolic stresses (hypoxia, nutrient deprivation)
Targeting Strategy	Antibody blockade (blocking protein interactions)	Small molecule inhibitors (interfering with enzyme activity, transport, receptor signals)
Representative Molecules	PD-1/PD-L1, CTLA-4, TIGIT, LAG-3, TIM-3	IDO1, CD73, LDHA, HIF-1α, AhR
Relationship	MIC extends the checkpoint paradigm by considering metabolic activity itself as a druggable immune regulatory switch. Both often act synergistically in the TME, forming a more complex inhibitory network	MIC extends the checkpoint paradigm by considering metabolic activity itself as a druggable immune regulatory switch

*MIC* Metabolic immune checkpoint, *pH* potential of hydrogen, *TME* Tumor microenvironment, *PD-1* Programmed cell death protein 1, *PD-L1* Programmed death-ligand 1, *CTLA-4* Cytotoxic T-lymphocyte-associated protein 4, *TIGIT* T-cell immunoreceptor with Ig and ITIM domains, *LAG-3* Lymphocyte-activation gene 3, *TIM-3* T-cell immunoglobulin and mucin-domain containing-3, *IDO1* Indoleamine 2,3-dioxygenase 1, *CD73* Cluster of differentiation 73, *LDHA* Lactate dehydrogenase A, *HIF-1α* Hypoxia-inducible factor 1-alpha, *AhR* Aryl hydrocarbon receptor

### Categorization and mechanisms of “metabolic immune checkpoints”

#### Metabolic enzyme and metabolite-regulated classical immune checkpoints (MIC-I)

**Fig. 2:**
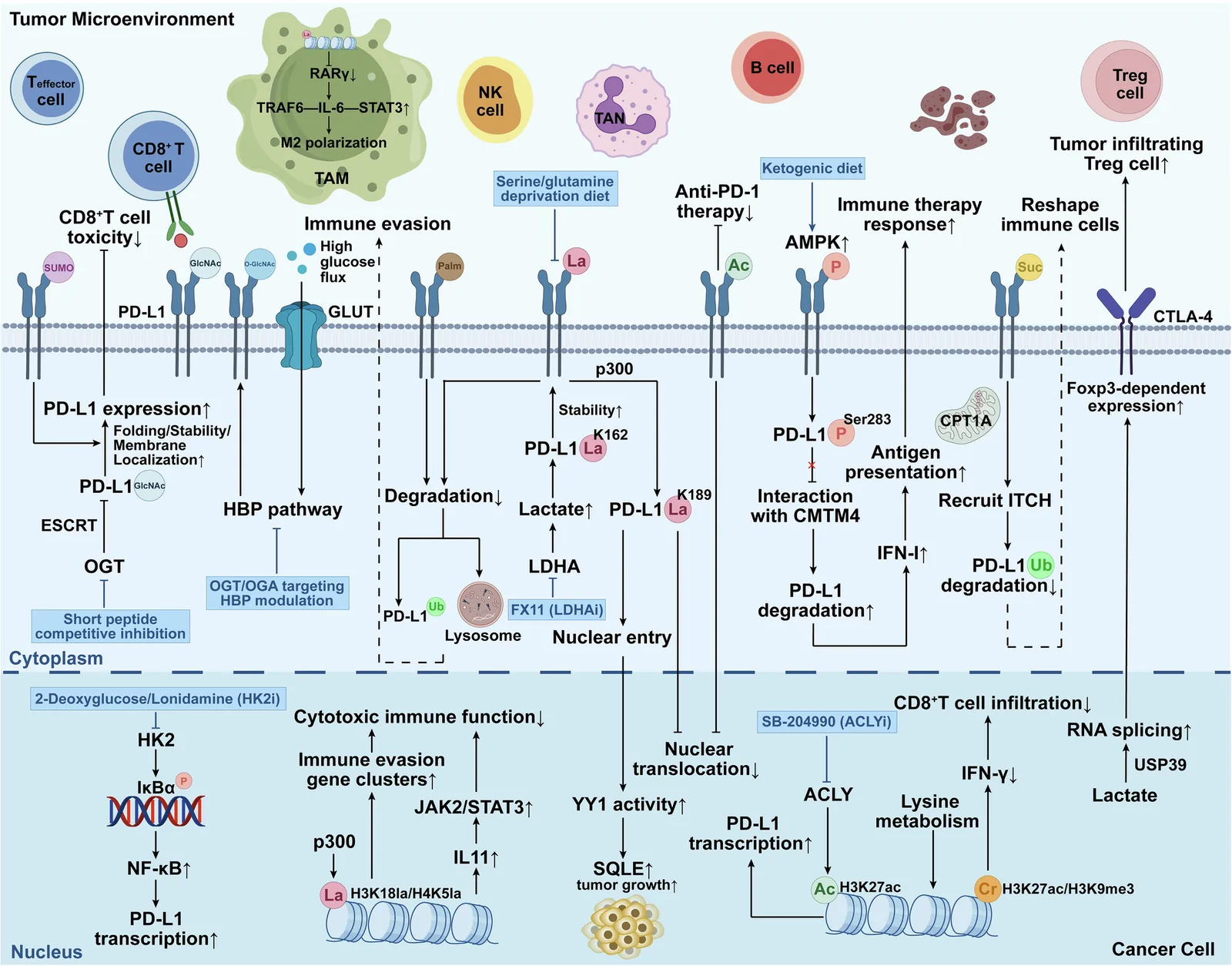
MIC-I: Metabolite-Mediated Modulation of Immune Checkpoint Pathways. MIC-I represents metabolite-mediated regulation of immune checkpoint pathways through posttranslational and epigenetic modifications. Reprogrammed glucose, lipid, and amino acid metabolism modulates the expression of classical immune checkpoints, such as PD-L1, and further regulates PD-L1 stability, subcellular localization, and nuclear translocation via glycosylation, lactylation, phosphorylation, acetylation, succinylation, and palmitoylation, thereby linking tumor metabolic states to immune evasion, immune dysfunction, and therapeutic response. RARγ Retinoic acid receptor gamma, TRAF6 TNF receptor-associated factor 6, IL-6 Interleukin-6, STAT3 Signal transducer and activator of transcription 3, NK cell Natural killer cell, TAN Tumor-associated neutrophil, Treg cell Regulatory T cell, TAM Tumor-associated macrophage, SUMO Small ubiquitin-like modifier, GlcNAc N-acetylglucosamine, O-GlcNAc O-linked N-acetylglucosamine, PD-1 Programmed cell death protein 1, PD-L1 Programmed death-ligand 1, GLUT Glucose transporter, Palm Palmitoylation, La Lactylation, Ac Acetylation, AMPK AMP-activated protein kinase, Suc Succinylation, CTLA-4 Cytotoxic T-lymphocyte-associated protein 4, ESCRT Endosomal sorting complex required for transport, OGT O-GlcNAc transferase, HK2 Hexokinase 2, IκBa Inhibitor of kappa B alpha, P Phosphorylation, NF-κB Nuclear factor kappa-light-chain-enhancer of activated B cells, HBP Hexosamine biosynthesis pathway, OGT/OGA O-GlcNAc transferase/O-GlcNAcase, Ub Ubiquitin, JAK/STAT3 Janus kinase/Signal transducer and activator of transcription 3, IL-11 Interleukin-11, LDHA Lactate dehydrogenase A, SQLE Squalene epoxidase, CMTM4 CKLF-like MARVEL transmembrane domain-containing protein 4, ACLY ATP-citrate lyase, CPT1A Carnitine palmitoyltransferase 1A, ITCH Itchy E3 ubiquitin protein ligase, IFNγ Interferon gamma, Cr Crotonylation, Foxp3 Forkhead box P3, USP39 Ubiquitin specific peptidase 39

MIC-I refers to a class of immune regulatory mechanisms in which metabolic enzymes or metabolites modulate classical immune checkpoint pathways, such as PD-1/PD-L1, CTLA-4, TIGIT, TIM-3, and LAG-3, predominantly at the posttranslational level. Distinct from transcriptional or genetic regulation, MIC-I mechanisms rely on metabolite-derived cofactors or acyl-group donors generated by metabolic reprogramming within the TME to dynamically modify immune checkpoint proteins and their associated signaling molecules. These metabolic cues regulate immune checkpoint activity mainly by altering protein stability, subcellular localization, and protein‒protein interactions through reversible posttranslational modifications (PTMs), thereby enabling rapid and context-dependent immune suppression without requiring de novo gene expression. As such, MIC-I represents a metabolically driven, nongenomic layer of immune checkpoint control that directly couples cellular metabolic states to immune evasion. For instance, lactate enhances PD-L1 protein stability through lactylation, thereby reinforcing its immunosuppressive role.^17^ Beyond this canonical example, tumor glycolytic enzymes such as LDHA promote PD-L1 lactylation at specific lysine residues (e.g., K162), directly enhancing PD-L1 stability and immune evasion in cancers such as NSCLC and colorectal cancer (CRC).^239,242,243^ Similarly, PD-L1 lactylation can delay its lysosomal degradation, a process that has been therapeutically explored through metabolic interventions such as serine/glutamine deprivation diets in colorectal cancer.^49^ More broadly, metabolic reprogramming in the TME not only reshapes energy and biosynthetic precursor supply but also generates metabolites that act as donors or cofactors for modifications such as lactylation, acetylation, glycosylation, and succinylation. These metabolites dynamically connect glucose metabolism, fatty acid oxidation, and TCA cycle fluxes to immune checkpoint regulation, thereby defining a class of metabolic-immune checkpoints (MIC-I). These dynamic molecular tags fine-tune immune checkpoint proteins, transcription factors, and signaling molecules by altering their conformation, stability, subcellular localization, or protein‒protein interactions, leading to rapid and reversible regulation of immune signaling and ultimately inhibiting T-cell activation and effector functions (Table 4, Fig. 2).

**Table 4:** MIC-I mechanisms and potential targeting strategies

MICs Category	Metabolic Pathway	Target/Axis	Regulatory Mechanism	Targeted Drugs/Strategies	Indication	Development Stage	Ref
**Metabolic Modification-Immune Checkpoints**	**Glucose Metabolism**	PD-L1 Glycosylation	OGT mediates PD-L1 glycosylation, disrupting ESCRT-mediated PD-L1 degradation, leading to increased PD-L1 expression and suppression of T-cell cytotoxicity	Short peptide competitive inhibition (HGS)	/	Preclinical	^266^
		PD-L1 Glycosylation (stability control)	Glycosylation promotes PD-L1 folding/stability/membrane localization; protects PD-L1 from degradation → sustained checkpoint function	Target N/O-glycosylation pathways; glycosylation-site targeting strategies	Multiple tumors	Preclinical	^263–265^
		HBP-driven O-GlcNAcylation	High glucose flux activates HBP → increased O-GlcNAcylation; dynamically tunes immune signaling proteins/TFs, reinforcing metabolic-immune regulatory networks	OGT/OGA targeting; HBP modulation	/	Preclinical	^267^
		PD-L1 Lactylation	PD-L1 lactylation delays lysosomal degradation of PD-L1 in tumor cells	Serine/glutamine deprivation diet	CRC	Phase I	^240^
		LDHA–PD-L1	Lactate accumulation mediated by LDHA promotes PD-L1 lactylation (K162 site), enhancing its protein stability	FX11 (LDHAi)	NSCLC, CRC	Preclinical	^239,242,243^
		PD-L1 lactylation “writers/erasers”	p300 (EP300)/AARS1/2/ACSS2/HBO1 act as lactylation writers; HDAC1-3/SIRT1-3 as delactylases; lactylation couples metabolic state to checkpoint/immune regulation	Target writers (e.g., EP300 axis); HDAC/Sirtuin modulation	/	Preclinical	^245–247^
		HK2–PD-L1	HK2 phosphorylates IκBα, promoting IκBα degradation, activating NF-κB, and upregulating PD-L1 transcription, promoting immune escape	2-Deoxyglucose (2-DG), Lonidamine (HK2i)	Glioma	Phase I	^320^
		Acetylation-dependent PD-L1 nuclear translocation	PD-L1 acetylation regulates nuclear translocation, which impacts response to anti-PD-1 therapy	Target acetylation/HDAC axis; combination with ICB	/	Preclinical	^258^
		USP39–CTLA4	Lactate promotes the RNA splicing mediated by USP39 to promote Foxp3-dependent expression of CTLA-4, maintaining the phenotype and functional state of tumor-infiltrating Treg cells	/	/	/	^449^
		Histone lactylation (immune-suppressive transcription programs)	p300, etc., catalyze histone lactylation (e.g., H3K18la/H4K5la); bromodomain/PHD proteins can “read” lactyl-marks; promotes glycolysis/angiogenesis/immune evasion gene clusters	Target lactylation writers/readers/erasers	/	Preclinical	^244–247,249,250,254^
		Histone lactylation → IL-11/JAK2/STAT3	Histone lactylation increases IL-11 expression → JAK2/STAT3 activation → suppresses cytotoxic immune functions	Target IL-11/JAK/STAT; lactylation modulation	/	Preclinical	^251^
		Histone lactylation in TAMs (RARγ/TRAF6-IL-6-STAT3)	Lactylation suppresses RARγ in macrophages → TRAF6-IL-6-STAT3 activation → pro-tumor TAM phenotype	Target STAT3/IL-6 axis; lactylation modulation	CRC	Preclinical	^252^
		Lactylation → M2 polarization	Histone lactylation drives macrophage polarization toward M2 immunosuppressive phenotype	Target lactylation or TAM reprogramming	/	Preclinical	^253^
		AMPK–PD-L1 Phosphorylation	A ketogenic diet activates AMPK, which phosphorylates PD-L1 (Ser283), disrupting its interaction with CMTM4 and promoting degradation. It also phosphorylates EZH2 to suppress PRC2, enhancing type I IFN and antigen presentation, thus boosting immune therapy response	AMPK agonist/Ketogenic diet combined with anti-CTLA-4	Solid tumors	Preclinical	^450^
	**Fatty Acid Metabolism**	CPT1A–PD-L1 Succinylation	Mitochondrial enzyme CPT1A mediates PD-L1 degradation via succinylation	Supplementation of metabolites (DES, DMK)/metabolic inhibitors	Melanoma	Preclinical	^259–261^
		PD-L1 Succinylation (stability control)	Succinate accumulation (e.g., SDH dysfunction) → PD-L1 succinylation recruits ITCH → blocks ubiquitination degradation; reshapes immune cells in TME	Target succinate/SDH axis; desuccinylase modulation	/	Preclinical	^259,261^
		PD-L1 Palmitoylation	Palmitoylation blocks PD-L1 ubiquitination and lysosome degradation → high PD-L1 surface levels → immune evasion	Palmitoylation inhibitors (DHHC targeting strategies)	/	Preclinical	^269^
		SUMOylation of immune regulators	SUMO attachment alters localization/stability/transcriptional activity of immune signaling proteins, fine-tuning immune pathways	SUMO pathway inhibitors	/	Preclinical	^268^
		ACLY–PD-L1	ACLY upregulates PD-L1 transcription via histone acetylation (H3K27ac)	SB-204990 (ACLYi)	Lung cancer	Preclinical	^124,255–257^
		Crotonylation (GBM stemness/IFN signaling)	Lysine metabolism rewiring increases crotonyl-CoA → histone crotonylation (e.g., H4cr) alters epigenetic marks (H3K27ac/H3K9me3) → IFN signaling & CD8^+^ T-cell infiltration changes → tumor progression	Target crotonyl-CoA production; epigenetic modulators	GBM	Preclinical	^260,262^
		PD-L1 K189 Lactylation/Delactylation Axis	p300 catalyzes PD-L1 K189 lactylation, inhibiting nuclear translocation, while HDAC2 mediates delactylation, promoting nuclear entry, enhancing YY1 activity, upregulating SQLE, and promoting liver cancer growth	HDAC2 inhibitors	HCC	Preclinical	^248^

*PD-L1* Programmed death-ligand 1, *OGT* O-GlcNAc transferase, *ESCRT* Endosomal sorting complexes required for transport, *HGS* Hepatocyte growth factor-regulated tyrosine, *HBP* Hexosamine biosynthesis pathway, *O-GlcNAcylation* O-linked β-N-acetylglucosaminylation, *TF* Transcription factor, *OGA* O-GlcNAcase, *CRC* Colorectal cancer, *LDHA* Lactate dehydrogenase A, *NSCLC* Non-small cell lung cancer, *AARS1/2* Alanyl-tRNA synthetase 1/2, *ACSS2* Acyl-CoA synthetase short chain family member 2, *HBO1* Histone acetyltransferase binding to ORC1, *HDAC1* Histone deacetylase 1, *SIRT1* Sirtuin 1, *HK2* Hexokinase 2, *IκBα* Nuclear factor of kappa light polypeptide gene enhancer in B cells inhibitor, alpha, *NF-κB* Nuclear factor kappa-light-chain-enhancer of activated B cells, *2-DG* 2-Deoxy-D-glucose, *PD-1* Programmed cell death protein 1, *ICB* Immune checkpoint blockade, *NAT10* N-acetyltransferase 10, *IFN* Interferon, *USP39* Ubiquitin specific peptidase 39, *CTLA4* Cytotoxic T-Lymphocyte-associated protein 4, *RNA* Ribonucleic acid, *FOXP3* Forkhead box P3, *Treg* Regulatory T cell, *H3K18la* Histone H3 lysine 18 lactylation, *H4K5la* Histone H4 lysine 5 lactylation, *PHD* Plant homeodomain, *p300* E1A binding protein p300, *IL-11* Interleukin-11, *JAK2* Janus kinase 2, *STAT3* Signal transducer and activator of transcription 3, *TAM* Tumor-associated macrophage, *RARγ* Retinoic acid receptor gamma, *TRAF6* TNF receptor associated factor 6, *AMPK* AMP-activated protein kinase, *CMTM4* CKLF-like MARVEL transmembrane domain containing 4, *EZH2* Enhancer of zeste homolog 2, *PRC2* Polycomb repressive complex 2, *CPT1A* Carnitine palmitoyltransferase 1A, *DES* Diethyl succinate, *DMK* Dimethyl α-ketoglutarate, *SDH* Succinate dehydrogenase, *ITCH* Itchy E3 ubiquitin protein ligase, *DHHC* Asp-His-His-Cys (palmitoyltransferase motif), *SUMO* Small ubiquitin-like modifier, *ACLY* ATP citrate lyase, *GBM* Glioblastoma, *YY1* Transcription factor YY1, *SQLE* Squalene epoxidase

##### Lactylation modification: from metabolic waste to epigenetic and signal regulatory factors

Among metabolic modifications, lactylation represents a paradigmatic MIC-I mechanism that directly couples glycolytic flux to immune checkpoint regulation. Lactylation is a newly identified posttranslational modification derived from lactate.^244^ This process is initiated by the conversion of intracellular lactate into lactyl-coenzyme A (lactyl-CoA) through acyl-transfer-related metabolic pathways. Lactyl-CoA subsequently serves as a lactyl-group donor, enabling lysine acetyltransferases (KATs) to transfer lactyl moieties onto lysine residues of histone and non-histone proteins. Accumulating evidence indicates that p300 (EP300), alanyl-tRNA synthetase 1/2 (AARS1/2), acetyl-CoA synthetase 2 (ACSS2) and histone acetyltransferase binding to ORC1 (HBO1) function as writers of protein lactylation, catalyzing lactylation at sites such as H3K18 and H4K5,^245,246^ thereby directly coupling cellular metabolic status to transcriptional regulation. These enzymes have also been implicated in PD-L1 lactylation, providing a direct mechanistic link between metabolic flux and immune checkpoint stabilization.^245–247^ Protein lactylation is a dynamic and reversible modification, and its removal depends on specific erasers. Class I histone deacetylases (HDAC1-3) and members of the sirtuin family (SIRT1-3) have been demonstrated to possess delactylase activity. By removing lactyl groups from histone and non-histone proteins, these enzymes regulate chromatin architecture, gene transcription, and signaling pathway activity.^247^ Notably, HDAC2-mediated delactylation of PD-L1 promotes its nuclear translocation, enhances YY1 transcriptional activity, upregulates squalene epoxidase (SQLE), and accelerates HCC progression.^248^ At present, the readers of lactylation are still being actively investigated. Emerging studies have revealed that proteins containing bromodomains or plant homeodomain (PHD) finger motifs can specifically recognize lactylated lysine residues. For example, tripartite motif containing 33 (TRIM33), brahma-related gene 1 (BRG1), and the double PHD finger protein (DPF2) bind to specific histone lactylation marks (such as H3K18la and H3K14la)^249,250^ and play critical roles in immune regulation, cell fate determination, and tumorigenesis.

Within immune regulation and the TME, lactylation exerts multifaceted biological functions. (1) Stabilization of immune checkpoint proteins Lactylation can directly modify PD-L1, inhibiting its ubiquitin-mediated degradation and thereby enhancing tumor cell-mediated suppression of T-cell activity.^243^ This process is further reinforced by glycolytic rewiring and LDHA activity, positioning LDHA-PD-L1 as a prototypical MIC-I axis.^239,242,243^ (2) Regulation of key signaling pathways and immune phenotypes histone lactylation promotes the expression of IL-11, leading to activation of the Janus kinase 2 (JAK2)/signal transducer and activator of transcription 3 (STAT3) signaling pathway and suppression of cytotoxic immune functions.^251^ This IL-11/JAK2/STAT3 axis represents a lactylation-driven immunosuppressive circuit in tumors. In colorectal cancer, histone lactylation suppresses the expression of retinoic acid receptor-γ (RARγ) in macrophages, thereby enhancing the TNF receptor-associated factor 6 (TRAF6)-IL-6-STAT3 signaling axis and conferring protumorigenic properties on these cells.^252^ Accumulating evidence indicates that histone lactylation facilitates macrophage polarization toward an immunosuppressive M2 phenotype.^253^ (3) Remodeling of the epigenetic landscape: histone lactylation, exemplified by H3K18la, activates the transcription of gene clusters involved in glycolysis, angiogenesis, and immune evasion, directly linking a high-lactate metabolic environment to protumorigenic transcriptional programs.^254^ Such immune-suppressive transcriptional programs are reinforced by lactylation readers and chromatin remodelers, establishing lactate as a bona fide epigenetic signaling molecule in the TME.^244–247,249,250,254^

##### Acetylation, succinylation, and other modifications

Beyond lactylation, multiple acylation-based modifications, including acetylation, succinylation, crotonylation, and glycosylation, form a layered regulatory network linking intermediary metabolism to immune checkpoint stability and signaling. Acetyl-CoA serves as the central donor for acetylation,^255^ and its intracellular availability is tightly regulated by metabolic enzymes such as ACLY and ACSS2.^256^ Acetylation is catalyzed by histone acetyltransferases (HATs) and can be removed by HDACs and members of the sirtuin family.^257^ Beyond histones, immune checkpoint proteins are also subject to acetylation-dependent regulation. Notably, the acetylation-dependent nuclear translocation of PD-L1 has been shown to critically determine the therapeutic efficacy of anti-PD-1 immunotherapy.^258^ In parallel, ACLY-driven histone acetylation (H3K27ac) upregulates PD-L1 transcription, defining an ACLY-PD-L1 metabolic-epigenetic axis in lung cancer.^124,255–257^

The TCA cycle intermediate succinate and the fatty acid β-oxidation intermediate crotonyl-CoA drive protein succinylation^259^ and crotonylation,^260^ respectively. Mutations in SDH lead to abnormal accumulation of succinate, resulting in widespread protein succinylation. Notably, succinylated PD-L1 has been shown to recruit the Itchy E3 ubiquitin ligase (ITCH), thereby inhibiting its own ubiquitination-mediated degradation. Moreover, PD-L1 succinylation modulates the composition and function of immune cells within the TME, creating an immunosuppressive milieu that favors tumor growth.^261^ Carnitine palmitoyltransferase 1 A (CPT1A)-mediated succinylation further promotes PD-L1 degradation, revealing a context-dependent role of succinylation in immune regulation, particularly in melanoma.^259–261^ In addition, glioblastoma stem cells undergo lysine metabolism reprogramming, which enhances the production of crotonyl-CoA and markedly increases global protein crotonylation, particularly histone H4 crotonylation. Histone crotonylation influences epigenetic marks such as H3K27ac and H3K9me3, thereby regulating interferon signaling and CD8^+^ T-cell infiltration, ultimately promoting tumor progression.^262^ Glycosylation, particularly N-linked and O-linked glycosylation, plays a critical role in the proper folding, stability, membrane localization, and ligand binding of immune checkpoint proteins.^263,264^ Glycosylation of PD-L1 has been shown to protect it from degradation mediated by glycoprotein glycosylation site-binding protein 1, thereby markedly enhancing PD-L1 stability.^265^ O-GlcNAc transferase (OGT)-mediated PD-L1 glycosylation disrupts endosomal sorting complexes required for transport (ESCRT)-mediated degradation and reinforces immune suppression, and competitive peptide inhibitors targeting this process have shown preclinical efficacy.^263–266^ In the TME, high glucose flux increases protein O-GlcNAcylation through activation of the HBP.^267^ This reversible and highly dynamic modification regulates the activity of numerous signaling proteins and transcription factors, forming an additional layer of metabolic-immune regulatory networks.

Beyond glycosylation, SUMOylation and palmitoylation also play essential roles in the regulation of immune checkpoint molecules. SUMOylation, which involves the covalent attachment of small ubiquitin-like modifier (SUMO) proteins to lysine residues, modulates protein subcellular localization, stability, and transcriptional activity,^268^ thereby fine-tuning immune-related signaling pathways. Palmitoylation is a reversible lipid posttranslational modification in which palmitic acid is covalently attached to cysteine residues of target proteins, enhancing their affinity for the plasma membrane. This modification regulates protein membrane localization, signal transduction efficiency, and protein‒protein interactions and plays a critical role in the spatial organization and functional regulation of immune receptors and immune checkpoint molecules. Emerging evidence indicates that tumor cells can promote palmitoylation of PD-L1, thereby blocking its ubiquitination and inhibiting lysosome-mediated degradation. This mechanism sustains high levels of PD-L1 on the cell surface and ultimately facilitates tumor immune evasion.^269^

Collectively, the posttranslational modifications discussed above, including lactylation, acetylation, and others, extensively regulate key immune checkpoints (e.g., PD-1/PD-L1, CTLA-4) as well as costimulatory molecules and cytokine receptors. These modifications collectively constitute metabolic modification-driven immune checkpoints (MIC-Is), an integrated regulatory layer that directly links tumor metabolic states to immune escape and offers actionable targets for combination therapies. However, it should be noted that current research has predominantly focused on the PD-1/PD-L1 axis, while the metabolic regulation of other critical checkpoints, such as TIGIT and LAG-3, remains less explored and warrants further investigation. The detailed mechanisms and functional consequences of these modifications are summarized in Table 4.

#### Metabolic factors as direct immune regulatory hubs (MIC-II)

**Fig. 3:**
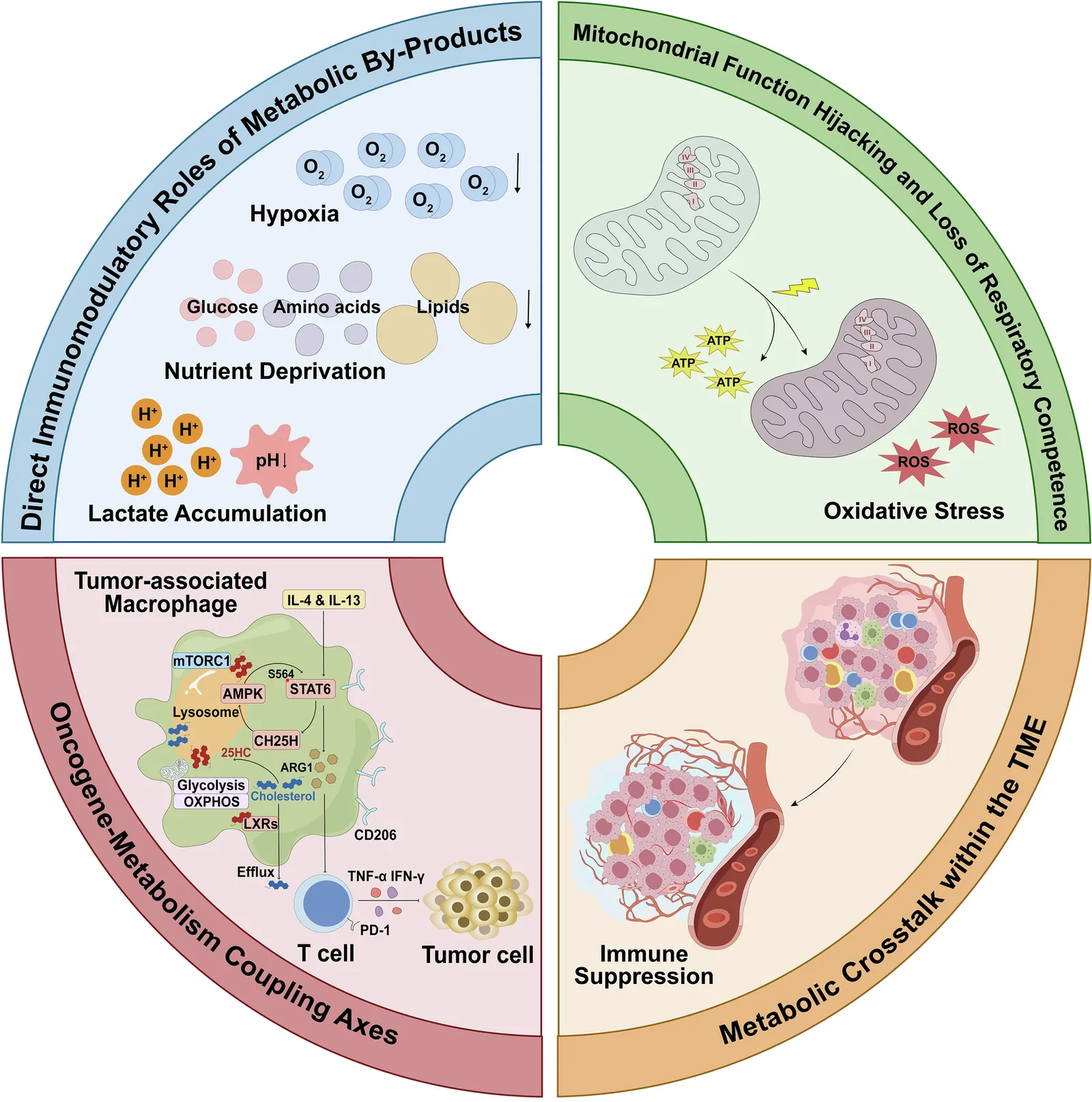
MIC-II: Metabolic Dependency and Nutrient Competition–Driven Immune Suppression. MIC-II encompasses immune suppression driven by metabolic dependency and nutrient competition within the TME. Key features include the direct immunomodulatory effects of metabolic byproducts, hijacking of mitochondrial function and loss of respiratory competence in immune cells, metabolic crosstalk among tumor, stromal, and immune compartments, and oncogene-metabolism coupling axes that reinforce nutrient sequestration and immunosuppressive signaling. Together, these processes induce metabolic stress, immune cell dysfunction, and exhaustion, thereby facilitating immune evasion and tumor progression. ATP Adenosine triphosphate; ROS Reactive oxygen species; mTORC1 Mechanistic target of rapamycin complex 1. IL-4 Interleukin-4, IL-13 Interleukin-13, AMPK AMP-activated protein kinase, STAT6 Signal transducer and activator of transcription 6, CH25H Cholesterol 25-hydroxylase, 25HC 25-Hydroxycholesterol, ARG1 Arginase 1, OXPHOS Oxidative phosphorylation, LXR Liver X receptor, CD206 Cluster of differentiation 206, TNF-α Tumor necrosis factor alpha, IFNγ Interferon gamma, PD-1 Programmed cell death protein 1, TME Tumor microenvironment

MIC-II encompasses metabolic enzymes, pathways, and their direct products that function as autonomous immune regulatory hubs, independent of classical immune checkpoint proteins. These factors constitute a class of nonclassical immune checkpoints, exerting profound immunomodulatory effects by reshaping the biochemical and energetic landscape of the TME. Unlike canonical immune checkpoints that primarily rely on ligand‒receptor interactions at the immunological synapse, MIC-II elements directly regulate immune cell fate and function through metabolic stress, signal metabolite sensing, epigenetic modulation, and organelle dysfunction. In this context, metabolic enzymes are no longer passive components of bioenergetics but acquire regulatory authority over immune surveillance and tumor immune escape. The specific mechanisms and therapeutic implications of MIC-II are explored in detail below (Table 5, Fig. 3).

**Table 5:** MIC-II mechanisms and potential targeting strategies

MICs Category	Metabolic Pathway	Target/Axis	Regulatory Mechanism	Targeted Drugs/Strategies	Indication	Development Stage	Ref.
**Metabolite-Immune Signal Axis**	**Glucose Metabolism**	Succinic Acid–SUCNR1	Succinic acid activates the PI3K/HIF-1α pathway via SUCNR1, enhancing macrophage migration and mediating TAM polarization, promoting tumor metastasis	NF-56-EJ40 (SUCNR1 antagonist)	CRC	Preclinical	^275^
		AMPK–mTOR	Tumor cells activate AMPK to competitively consume glucose from the microenvironment, inhibiting T-cell mTOR signaling	Metformin	Breast cancer, ESCC	Phase II	^289^
		OXCT1–ARG1–M2 TAMs	OXCT1 promotes transcription of ARG1 in tumor-associated TAMs at the epigenetic level	OXCT1 inhibitor PZ	Liver cancer	Preclinical	^298^
		GLUT1/2/4–CD8^+^ T	Tumor cells overtake glucose → TME glucose depletion → T-cell function suppression	BAY-876 (GLUT1i)	Pancancer	Preclinical	^288^
		MCT1/MCT4–NK	Lactate export → microenvironment acidification → NK cell cytotoxicity decrease	AZD3965 (MCT1/4i)	Lymphoma	Phase II	^273^
		ALDOB–Treg	Downregulation of glycolytic enzyme ALDOB leads to upregulation of TGF-β, increasing Tregs and impairing CD8^+^ T-cell function in the TME	TGF-β inhibitors	Liver cancer	Preclinical	^299^
		PDH–NF-κB	Decreased levels of cytoplasmic PDH complex E1α (PDHE1α) restore NF-κB signaling, promoting tumor immune escape	/	Lung cancer	/	^300^
		IDH/D-2HG–CD8^+^ T	IDH R132H mutation in tumor cells secretes D-2HG, inhibiting CD8^+^ T-cell activation and subsequent proliferation and immune killing	/	/	/	^278^
		LDHB–G6PD–NADPH–CD8^+^ T	LDHB inhibits G6PD→decreased NADPH synthesis→cystine transported by SLC7A11 cannot be reduced to cysteine→oxidative cysteine accumulation→cytoskeleton disruption→CD8^+^ T-cell exhaustion and death	Enhance G6PD activity or regulate SLC7A11 transport	Pancancer	Preclinical	^279^
	**Fatty Acid Metabolism**	CPT1A–T-cell exhaustion	Inhibition of CPT1A activity or expression makes cancer cells more susceptible to cytotoxic T lymphocyte destruction	Etomoxir (CPT1Ai)	Breast cancer, leukemia, glioma	Preclinical	^451^
		CD36–TAMs	TAMs take up long-chain fatty acids through CD36, form lipid droplets (dependent on DGAT1), and inhibit FAO	Anti-CD36 antibody	TNBC, ovarian cancer	Preclinical	^293^
		LXR/SREBP2–T cells	Oxysterols mediate the interaction of the LXR and SREBP2 pathways, causing T-cell cholesterol deficiency, followed by abnormal metabolism and signaling pathways, leading to T-cell exhaustion	CAR-T-cell LXRb knockout	Solid tumors	/	^294^
		Riplet–FASN–STAT3–CD8^+^ T	Riplet silencing→FASN deubiquitination enhances FAS→Palmitic acid activates STAT3 palmitoylation in CD8^+^ T cells→terminal exhaustion ↑ →anti-PD-1 resistance	FASN inhibitors	HCC	Preclinical	^296^
		CH25H–25HC–GPR155–AMPKα–STAT6	IL-4/IL-13→activate STAT6→upregulate CH25H → AMPKα phosphorylates STAT6 (S564)→enhance ARG1 expression→macrophage polarization to immune suppressive phenotype, inhibiting CD8^+^ T-cell function	Target CH25H/block 25HC synthesis/combined ICB therapy	Solid tumors	Preclinical	^11^
		PLA2G4A–lipid droplet accumulation–T-cell senescence	Tumor cells and Tregs upregulate PLA2G4A expression in responsive T cells→induce lipid metabolic imbalance and lipid droplet accumulation→induce T-cell senescence→inhibit antitumor function	PLA2G4A inhibitor	Melanoma, breast cancer	Preclinical	^295^
	**Amino Acid Metabolism**	GLS1–M2 TAM polarization	Tumor cells regulate GLS1 vesicle secretion through CDC42-IQGAP1, increasing glutamine metabolism to promote macrophage M2 polarization and angiogenesis	GLS1 inhibitor	HER2-positive gastric cancer	/	^291^
		IDO1/TDO–Kyn–AhR	Tryptophan→kynurenine→AhR activation→Treg differentiation↑	Epacadostat (IDO1i)	Melanoma	Phase III (Failure)	^241^
		ASCT2–TAM	Blocking glutamine transport inhibits TAM glutamine uptake, preventing its conversion to M2-like macrophages	V-9302 (ASCT2i)	Lung cancer, glioblastoma	Preclinical	^292^
		ARG1–T cells	Arginase 1-mediated arginine depletion→T-cell function suppression	INCB001158 (ARG1 inhibitor)	Solid tumors	Phase I/II	^290^
		KEAP1/NRF2	KEAP1 mutation→NRF2 activation→enhanced glutathione synthesis, inhibiting neutrophil NET formation	CB-839 (Telaglenastat) +PD-1	NSCLC	Phase II	^301^
		Spermidine–CD8^+^ T-cell	Tumor-derived SPD reduces CD8^+^ T-cell quantity and cytotoxicity, inducing immune suppression and promoting GBM progression	ODC inhibitors (e.g., DFMO) or SPD removal strategies	GBM	Preclinical	^276^
		Ammonia–ASL/FOXP3–SMS–Spermine–PPARγ–Tregs	Ammonia kills effector T cells; Tregs adapt via ASL and FOXP3–SMS–spermine–PPARγ	Target ammonia production; block SMS/spermine–PPARγ	HCC	Preclinical	^277^
		NAT8L–NAA–PCAF–lamin A	Tumor cells upregulate NAT8L, synthesizing metabolite NAA→promotes PCAF-mediated Lamin A-K542 acetylation→blocking integration with SUN2, disrupting immunological synapse formation→inhibiting CD8^+^ T/NK cell cytotoxicity, promoting immune escape	Target NAT8L	HER2^+^ breast cancer (anti-PD-L1 treatment resistance)	Preclinical	^302^
	**Mitochondrial Metabolism**	PKM2/HIF-1α–CD8^+^ T	PKM2 reprograms mitochondrial metabolism, enhancing CD8^+^ T-cell and CAR-T-cell effector functions, boosting antitumor activity	TEPP-46 (PKM2 activator)	Melanoma	Preclinical	^286^
		Mitochondrial complex I–ROS	Inhibition of the ETC → ROS accumulation→tumor cell apoptosis/immunogenic death↑	IACS-010759	AML, solid tumors	Phase I	^281^
		Mitochondrial complex I–CD4^+^ T	Inhibition of mitochondrial complex I promotes DC maturation and enhances costimulation of CD4^+^ T cells, promoting antitumor immunity	Metformin	Solid tumors	Clinical application	^191^
		DHODH–Treg	Inhibition of mitochondrial pyrimidine synthesis→blocking tumor proliferation and Treg differentiation	Brequinar (DHODHi)	Leukemia, melanoma	Phase I	^284^
		Mitochondrial TCA cycle–CD8^+^ T	CD8^+^ T-cell migration depends on ATP and ROS generated by glucose and glutamine-driven TCA cycle; enhancing mitochondrial function boosts T-cell migration and tumor infiltration	Mitochondrial metabolism enhancers/TCA cycle activation strategies	Solid tumors (CAR-T therapy)	Preclinical	^285^

*SUCNR1* Succinate receptor 1, *PI3K* Phosphoinositide 3-kinase, *HIF-1α* Hypoxia-inducible factor 1-alpha, *CRC* Colorectal cancer, *AMPK* AMP-activated protein kinase, *mTOR* Mechanistic target of rapamycin, *ESCC* Esophageal squamous cell carcinoma, *OXCT1* 3-oxoacid CoA-transferase 1, *ARG1* Arginase 1, *PZ* Pimozide, *GLUT1/2/4* Glucose transporter type 1/2/4, *MCT1/MCT4* Monocarboxylate transporter 1/4, *NK* Natural killer cell, *ALDOB* Aldolase, fructose-bisphosphate B, *TGF-β* Transforming growth factor beta, *Treg* Regulatory T cell, PDH Pyruvate dehydrogenase, NF-κB Nuclear factor kappa-light-chain-enhancer of activated B cells, IDH Isocitrate dehydrogenase, *D-2HG* D-2-hydroxyglutarate, *LDHB* Lactate dehydrogenase B, *G6PD* Glucose-6-phosphate dehydrogenase, *NADPH* Nicotinamide adenine dinucleotide phosphate, *SLC7A11* Solute carrier family 7 member 11, *CPT1A* Carnitine palmitoyltransferase 1A, *CD36* Cluster of differentiation 36, *DGAT1* Diacylglycerol O-acyltransferase 1, *FAO* Fatty acid oxidation, *TNBC* Triple-negative breast cancer, *LXR* Liver X receptor, *SREBP2* Sterol regulatory element-binding protein 2, *CAR-T* Chimeric antigen receptor T cell, *Riplet* RING finger protein 135 (RNF135), FASN Fatty acid synthase, STAT3 Signal transducer and activator of transcription 3, HCC Hepatocellular carcinoma, *PD-1* Programmed cell death protein 1, *CH25H* Cholesterol 25-hydroxylase, *25HC* 25-hydroxycholesterol, *GPR155* G protein-coupled receptor 155, *IL-4/IL-13* Interleukin-4/interleukin-13, *ARG1* Arginase 1, *ICB* Immune checkpoint blockade, *PLA2G4A* Phospholipase A2 group IVA, *GLS1* Glutaminase 1, *CDC42* Cell division cycle 42, *IQGAP1* IQ motif containing GTPase activating protein 1, *HER2* Human epidermal growth factor receptor 2, *IDO1* Indoleamine 2,3-dioxygenase 1, *TDO* Tryptophan 2,3-dioxygenase, *Kyn* Kynurenine, *AhR* Aryl hydrocarbon receptor, *ASCT2* Alanine-serine-cysteine transporter 2, *KEAP1* Kelch-like ECH-associated protein 1, *NRF2* Nuclear factor erythroid 2–related factor 2, *CB-839* Telaglenastat, *NSCLC* Non-small cell lung cancer, *SPD* Spermidine, *GBM* Glioblastoma, *ASL* Argininosuccinate lyase, *FOXP3* Forkhead box P3, *SMS* Spermine synthase, *PPARγ* peroxisome proliferator-activated receptor gamma, *Tregs* Regulatory T cells, *HCC* Hepatocellular carcinoma, *ODC* Ornithine decarboxylase, *DFMO* Difluoromethylornithine, *NAT8L* N-acetyltransferase 8-like, *NAA* N-acetylaspartate, *PCAF* P300/CBP-associated factor, *SUN2* Sad1 and UNC84 domain containing 2, *PD-L1* Programmed death-ligand 1, *PKM2* Pyruvate kinase M2, *CAR-T* Chimeric antigen receptor T cell, *ETC* Electron transport chain, *ROS* Reactive oxygen species, *AML* Acute myeloid leukemia, *DC* Dendritic cell, *DHODH* Dihydroorotate dehydrogenase, *TCA* Tricarboxylic acid cycle

##### Direct immunomodulatory roles of metabolic byproducts

Metabolic byproducts that accumulate within the TME have emerged as some of the most potent metabolic immune checkpoints of the MIC-II category. Rather than representing inert waste products, these metabolites function as bioactive signaling entities capable of directly reprogramming immune cell metabolism, differentiation trajectories, and effector functions. By altering extracellular pH, intracellular metabolic flux, receptor-mediated signaling, and epigenetic regulation, metabolic byproducts impose a pervasive immunosuppressive pressure that operates independently of classical immune checkpoint pathways.

LDHA represents a prototypical MIC-II enzyme. By catalyzing pyruvate-to-lactate conversion, LDHA drives lactate accumulation and extracellular acidification within the TME.^239,242^ This acidic milieu directly suppresses TCR signaling,^270^ cytokine secretion, and cytotoxic activity, thereby limiting antitumor immunity.^271^ Beyond metabolic flux control, LDHA-driven lactate acts as a metabolic-immune coupling factor, reinforcing tumor immune evasion through both cell-intrinsic and microenvironmental mechanisms. This principle extends to other key immunosuppressive metabolites. Lactate, beyond its role in acidification, is actively imported into immune cells via MCTs, disrupting intracellular NAD^+^/NADH balance and limiting metabolic plasticity.^272^ Lactate also engages the G protein-coupled receptor GPR81 to drive macrophage polarization toward an M2-like immunosuppressive phenotype and more broadly acts as a metabolic signal across multiple immune cell types, thereby cooperatively constructing an immunosuppressive network.^129^ The druggability of lactate trafficking is reflected by efforts to pharmacologically block its transport, exemplified by AZD3965 (MCT1/4 inhibitor) in phase II lymphoma trials,^273^ which conceptually aims to relieve lactate-driven metabolic immune suppression.

Analogously, tryptophan catabolism generates kynurenine through IDO1/TDO2, and kynurenine functions as an endogenous ligand for AhR to suppress CD8^+^ T-cell proliferation, promote apoptosis, and enforce exhaustion-associated transcriptional states while promoting Treg differentiation and tolerogenic DC programs.^148^ Importantly, this axis has been intensely explored in the clinic; however, the complexity of metabolite checkpoint targeting is illustrated by epacadostat (IDO1 inhibitor^241^), which advanced to phase III melanoma but failed to improve outcomes, underscoring that effective MIC-II blockade may require biomarker-guided stratification or rational combinations. In parallel, extracellular nucleotide metabolism generates adenosine through the CD39/CD73 cascade, and adenosine engages A2A/A2B receptors to elevate cAMP and activate PKA signaling, potently suppressing T/NK activation, proliferation, granule release, and cytokine production while stabilizing Tregs and reinforcing MDSC suppression,^226,227^ features that make the adenosine pathway a canonical metabolite checkpoint that can be intercepted at either the enzymatic generation or receptor sensing level.

Beyond these classical metabolite checkpoints, emerging MIC-II examples indicate that metabolite-receptor signaling and redox stress can also function as direct immune barriers. Under hypoxia or mitochondrial stress, succinate accumulates intracellularly to stabilize HIF-1α via inhibition of α-ketoglutarate-dependent dioxygenases and extracellular signals through succinate receptor 1 (SUCNR1 [GPR91]) to reshape macrophage and DC behavior^274^; preclinical intervention of succinate sensing has been exemplified by NF-56-EJ40 (SUCNR1 antagonist) in CRC preclinical models.^275^ Likewise, polyamine pathways can impose immunosuppression, as tumor-derived spermidine has been linked to reduced CD8^+^ T-cell abundance and cytotoxicity in glioblastoma models, motivating strategies such as difluoromethylornithine (DFMO; ornithine decarboxylase [ODC] inhibitor) or spermidine removal approaches at the preclinical stage.^276^ In HCC, ammonia-rich metabolic niches generated by high glutaminolysis selectively eliminate CD8^+^/CD4^+^ effector T cells while promoting Treg adaptation via urea-cycle detoxification and forkhead box p3 (FOXP3)-spermine synthase (SMS)-spermine signaling, reinforcing immunosuppression and contributing to anti-PD-1 resistance.^277^ Finally, a growing set of tumor-intrinsic metabolic nodes can directly translate metabolic wiring into immune dysfunction, including mutant isocitrate dehydrogenase (IDH)-mediated secretion of D-2-hydroxyglutarate (D-2HG), which suppresses CD8^+^ T-cell activation,^278^ and a lactate-redox-cytoskeleton coupling axis in which LDHB restrains glucose-6-phosphate dehydrogenase (G6PD)-dependent NADPH production, thereby limiting SLC7A11-dependent cystine reduction,^279^ driving oxidative cystine accumulation and cytoskeletal disruption to promote T-cell exhaustion and death, conceptually suggesting immune-restorative directions such as enhancing G6PD activity or modulating SLC7A11 transport in preclinical settings. Together, these examples emphasize that MIC-II metabolite checkpoints span waste-product toxicity, receptor‒ligand signaling, and intracellular redox-metabolic constraints, forming an integrated suppressive layer over antitumor immunity.

##### Mitochondrial function hijacking and loss of respiratory competence

Mitochondrial integrity and respiratory competence represent a metabolic hard requirement for sustained antitumor immunity, particularly for T-cell persistence, migration, and memory formation^280^; therefore, tumor-driven disruption of immune mitochondrial fitness functions as a powerful MIC-II layer that suppresses immunity at the level of bioenergetics, redox control, and survival. Therapeutically, this creates a nuanced landscape in which mitochondrial manipulation can either promote tumor cell death or recondition immune function depending on context. For instance, inhibition of mitochondrial complex I has been explored to induce tumor apoptosis or immunogenic death, with IACS-010759 (mitochondrial complex I inhibitor) entering phase I studies in acute myeloid leukemia (AML) and solid tumors.^281^ Conversely, mitochondrial complex I-linked metabolic modulation has also been associated with immune potentiation; metformin, a widely used clinical drug, has been reported to promote DC maturation and enhance costimulation of CD4^+^ T cells,^191^ illustrating how mitochondrial-targeting pharmacology can reshape antigen presentation and T-cell priming.

Beyond ETC targeting, mitochondrial metabolism intersects with lymphocyte lineage decisions and can be leveraged to selectively restrain suppressive immune subsets. Mitochondrial pyrimidine synthesis via dihydroorotate dehydrogenase (DHODH) has been implicated in supporting tumor proliferation and Treg differentiation,^282,283^ motivating DHODH blockade as an immunometabolic intervention; Brequinar (DHODH inhibitor) has advanced to phase I testing in leukemia and melanoma.^284^ At the level of effector trafficking, glucose- and glutamine-driven TCA cycle activity supplies ATP and ROS that support CD8^+^ T-cell migration and tumor infiltration, implying that strategies aimed at enhancing mitochondrial function or activating TCA cycle flux may strengthen adoptive cell therapy performance in preclinical solid tumor settings.^285^ Finally, mitochondrial metabolism is not only a vulnerability but also a programmable lever for T-cell function: activation of PKM2 has been linked to improved mitochondrial metabolism and enhanced effector functions in CD8^+^ T cells and chimeric antigen receptor T (CAR-T) cells, supported by TEPP-46 (PKM2 activator) in preclinical (animal) melanoma models.^286^ Together, these examples reinforce that mitochondrial MIC-II mechanisms are therapeutically actionable but require context-aware designs to avoid collateral suppression of immune bioenergetics.

##### Metabolic crosstalk within the TME

Metabolic crosstalk in the TME forms a cooperative ecosystem in which tumor cells, stromal cells, and myeloid populations exchange metabolites and collectively construct nutrient deprivation traps that suppress effector immunity. By continuously depleting key metabolic substrates for immune cells, this crosstalk directly restricts their functional potential, thereby operating as a distributed, non-receptor-dependent MIC-II mechanism. Within this architecture, competition for glucose is particularly central. Tumors can enforce T-cell metabolic paralysis by outcompeting immune cells for glucose,^287^ which has motivated strategies targeting tumor glucose uptake (e.g., the GLUT1 inhibitor BAY‑876 in preclinical studies). In parallel, broader metabolic rebalancing approaches aimed at restoring immune‑accessible nutrients and re‑enabling T-cell anabolic signaling^288^ have been explored clinically, such as metformin in phase II trials for breast cancer and esophageal squamous cell carcinoma (ESCC).^289^ Nutrient depletion is further amplified by myeloid and stromal programs: TAM-associated arginine consumption suppresses T-cell proliferation and function, creating a strong rationale for arginine-restoring interventions such as INCB001158 (ARG1 inhibitor) in phase I/II trials across solid tumors.^290^ Crosstalk is not limited to glucose and arginine. Glutamine metabolism and transport are frequently rewired to support suppressive myeloid states, exemplified by tumor-regulated vesicular secretion programs that increase macrophage glutamine metabolism through GLS1 and promote M2 polarization and angiogenesis, thereby motivating GLS1 inhibitor strategies^291^; in parallel, blocking glutamine import can directly restrain suppressive macrophage polarization, as illustrated by V-9302 (ASCT2 inhibitor) in preclinical lung cancer and glioblastoma models.^292^ Lipid crosstalk similarly reinforces immune escape: TAM uptake of long-chain fatty acids via CD36 promotes lipid droplet programs and dysfunctional fatty acid oxidation, supporting the development of anti-CD36 antibodies in preclinical triple-negative breast cancer (TNBC) and ovarian cancer.^293^ In T cells, cholesterol homeostasis can be disrupted by oxysterol-driven liver X receptor (LXR)-sterol regulatory element-binding protein 2 (SREBP2) crosstalk, causing cholesterol deficiency and exhaustion-like dysfunction, motivating engineered approaches such as LXRβ knockout CAR-T cells in solid tumor contexts.^294^

Notably, metabolic crosstalk also includes tumor- and Treg-driven lipid remodeling that forces effector T cells into dysfunctional states. For example, tumor cells and Tregs can upregulate phospholipase A2 Group IVA (PLA2G4A) in responsive T cells, inducing lipid droplet accumulation and driving T-cell senescence, consistent with interest in PLA2G4A inhibitors in preclinical melanoma and breast cancer.^295^ Tumor lipid synthesis programs can also impose exhaustion programs by signaling lipid species: Riplet silencing can enhance FASN activity and palmitate availability, enabling STAT3 palmitoylation in CD8^+^ T cells and promoting terminal exhaustion and anti-PD-1 resistance, thereby supporting the exploration of FASN inhibitors in preclinical HCC.^296^ Moreover, Th2 cytokine-driven IL-4/IL-13-STAT6 signaling can upregulate CH25H and increase 25-hydroxycholesterol (25HC) production, which engages GPR155 and triggers AMPKα-dependent STAT6 phosphorylation to enhance ARG1 expression and polarize macrophages toward suppressive phenotypes, motivating strategies to target CH25H, block 25HC synthesis, or combine with immune checkpoint blockades (ICBs) in preclinical solid tumor models.^11^ Collectively, these examples show that metabolic crosstalk is a multinutrient, multicellular system in which transporters, lipid handling, and myeloid polarization programs cooperate to produce durable immune exclusion.

##### Oncogene-metabolism coupling axes

Oncogenic signaling pathways act as upstream architects of metabolic reprogramming and indirectly impose immune suppression by dictating how tumors acquire nutrients, allocate carbon, and generate immunomodulatory metabolites. The PI3K-AKT-mTOR axis is a central integrator of growth signals and metabolism, and its immunological footprint is increasingly leveraged via metabolic repurposing^297^; for example, tumor cell AMPK activation can support competitive glucose consumption that suppresses T-cell mTOR signaling, providing rationale for metformin in phase II studies (e.g., breast cancer and ESCC).^289^ In parallel, direct targeting of nutrient transport can intercept oncogene-supported nutrient monopolization, consistent with interest in BAY-876 (GLUT1 inhibitor) at the preclinical stage.^288^ Beyond nutrient uptake, oncogene-metabolism coupling can program immunosuppressive myeloid states through epigenetic and transcriptional control. In liver cancer models, ketone-body oxidation machinery has been implicated in shaping TAM suppression: 3-oxoacid CoA-transferase 1 (OXCT1) can epigenetically promote ARG1 transcription in TAMs, motivating the OXCT1 inhibitor PZ in preclinical studies as a strategy that targets myeloid polarization rather than tumor cell proliferation alone.^298^ Tumor-intrinsic glycolytic wiring can also indirectly elevate immunosuppressive cytokines and Treg recruitment; for example, downregulation of fructose-bisphosphate aldolase B (ALDOB) has been associated with upregulation of TGF-β, increasing Tregs and impairing CD8^+^ T-cell function, thereby supporting interest in TGF-β inhibitors in preclinical liver cancer contexts.^299^ Additional tumor-intrinsic metabolic rewiring nodes may connect metabolism to inflammatory transcription: reduced cytoplasmic PDH complex E1α (PDHE1α) has been suggested to restore nuclear factor kappa-light-chain-enhancer of activated B cells (NF-κB) signaling and promote immune escape in lung cancer,^300^ highlighting PDH-NF-κB as a mechanistic MIC-II coupling point even where direct pharmacologic strategies remain immature.

Amino acid and redox programs controlled by oncogene-associated rewiring further shape immune landscapes. Kelch-like ECH-associated protein 1 (KEAP1) mutation-driven NRF2 activation enhances glutathione synthesis and can suppress neutrophil extracellular trap (NET) formation, providing a rationale for combining glutaminase blockade with immunotherapy, exemplified by CB-839 (telaglenastat) plus PD-1 blockade in phase II NSCLC studies.^301^ In parallel, arginine and glutamine constraints can be therapeutically relieved or rebalanced through INCB001158 (ARG1 inhibitor) in phase I/II trials^290^ and V-9302 (ASCT2 inhibitor) in preclinical settings,^292^ respectively. Finally, emerging metabolite-protein modification axes illustrate how oncogenic metabolic outputs can disable immune synapse formation: tumor upregulation of N-acetyltransferase 8-like (NAT8L) increases N-acetylaspartate (NAA) production, which promotes P300/CBP-associated factor (PCAF)-mediated lamin A-K542 acetylation, disrupts lamin A-Sad1 and UNC84 domain containing 2 (SUN2) coupling, and impairs immunological synapse stability, motivating NAT8L targeting strategies in preclinical HER2^+^ breast cancer models with anti-PD-L1 resistance.^302^ Together, these integrated examples indicate that oncogene–metabolism coupling provides a mechanistic bridge from genetic lesions to metabolite checkpoints, myeloid suppression and immune synapse dysfunction, thereby offering multiple intervention points for combination immunotherapy.

Collectively, the mechanisms described above define MIC-II as a distinct class of nonclassical immune checkpoints. Unlike canonical checkpoints, MIC-II operates through autonomous metabolic programs mediated by bioactive metabolites, mitochondrial dysfunction, nutrient competition, and oncogene-driven rewiring, which impose fundamental constraints on immune cell bioenergetics, redox state, and epigenetic regulation. This form of immunosuppression is diffuse, context dependent, and embedded within the broader metabolic architecture of the TME, contributing not only to immune dysfunction but also to primary and acquired resistance to ICB therapies. Therapeutically, targeting MIC-II represents a paradigm shift: rather than directly blocking immune inhibitory receptors, intervention aims to restore metabolic permissiveness in the TME, thereby re‑enabling endogenous antitumor immunity. Rational combination strategies that integrate metabolic modulators with immunotherapies thus hold considerable promise for overcoming these pervasive metabolic barriers and achieving durable clinical responses.

#### Microbiome-metabolism-immune axis-mediated checkpoints (MIC-III)

**Fig. 4:**
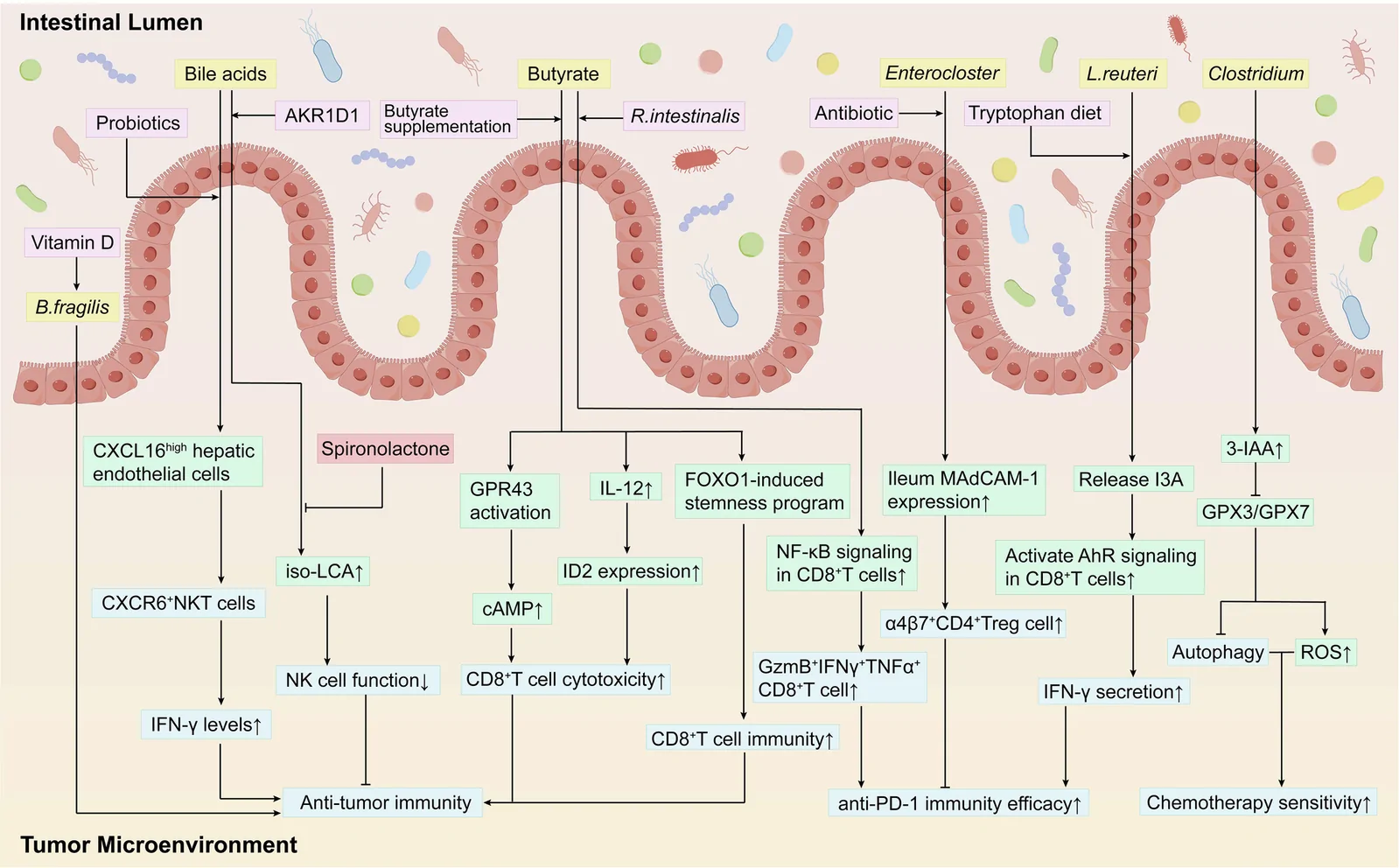
MIC-III: Microbiota-Metabolite-Immune Interaction Networks. MIC-III depicts microbiota-metabolite-immune interaction networks in which gut microbiota-derived metabolites act as systemic immunomodulators shaping antitumor immunity. Microbial metabolites, including bile acids, short-chain fatty acids, tryptophan derivatives, polyamines, and other bioactive compounds, regulate immune cell differentiation, trafficking, metabolic fitness, and effector function through host metabolic and immune signaling pathways. These interactions influence immune checkpoint blockade efficacy, chemotherapy sensitivity, and tumor immune tolerance across multiple cancer types. *B. fragilis* *Bacteroides fragilis*, CXCL16 C-X-C motif chemokine ligand 16, CXCR6 C-X-C motif chemokine receptor 6, IFNγ Interferon gamma, iso-LCA Isolithocholic acid, NK Natural killer, GPR43 G protein-coupled receptor 43, cAMP Cyclic adenosine monophosphate, IL-12 Interleukin-12, ID2 Inhibitor of DNA binding 2, FOXO1 Forkhead box O1, NF-κB Nuclear factor kappa B, GZMB Granzyme B, TNF-α Tumor necrosis factor alpha, MAdCAM-1 Mucosal addressin cell adhesion molecule-1, α4β7 Alpha-4 beta-7 integrin, Treg Regulatory T cell, PD-1 Programmed cell death protein 1, AhR Aryl hydrocarbon receptor, I3A Indole-3-aldehyde, 3IAA Indole-3-acetic acid, GPX3 Glutathione peroxidase 3, GPX7 Glutathione peroxidase 7, ROS Reactive oxygen species, AKR1D1 Aldo-keto reductase family 1 member D1, *R. intestinalis Roseburia intestinalis, L. reuteri Limosilactobacillus reuteri*

MIC‑III represents microbial‑derived metabolites that function as systemic regulators of antitumor immunity. Produced by commensal communities in the gut, tumors, and mucosal barriers, these compounds act as distal immunomodulatory signals, operating independently of classical immune checkpoint pathways. Through ecological and systemic metabolic crosstalk, MIC‑III components shape immune cell differentiation and functional polarization, linking microbial ecology directly to host immunometabolic states. This category illustrates how tumor‑immune dynamics are modulated by a cross‑tissue, organism‑wide network rooted in microbiome–host interactions (Table 6, Fig. 4).

**Table 6:** MIC-III mechanisms and potential targeting strategies

MICs Category	Target/Axis	Regulatory Mechanism	Targeted Drugs/Strategies	Indication	Development Stage	Ref
**Microbiome-Metabolism-Immune Axis**	AKR1D1–bile acids–NK	AKR1D1 deficiency promotes dysbiosis and accumulation of iso-LCA, impairing NK cell cytotoxic function	Spironolactone (targeting iso-LCA)	Liver cancer	Preclinical	^310^
	Bile acids–CXCL16–CXCR6^+^ NKT	Gut microbiota regulates primary to secondary bile acid conversion→increased CXCL16 expression in hepatic endothelial cells→accumulation of CXCR6^+^ NKT cells in the liver→elevated IFN-γ levels→mediates liver-specific antitumor immunity	Regulate gut microbiota (e.g., probiotics, antibiotics), bile acid metabolism intervention	Primary liver cancer, liver metastasis	Preclinical (Animal)	^311^
	Butyrate–GPR43	Gut microbiota produces butyrate→GPR43 activation→cAMP ↑ →CD8^+^ T-cell cytotoxicity ↑ →antitumor immunity↑	Butyrate supplementation	CRC	Preclinical	^304^
	Butyrate–FOXO1–CD8^+^ T	Microbiota-derived butyrate promotes a FOXO1-induced stemness program and preserves CD8^+^ T-cell immunity against melanoma	Butyrate supplementation	Melanoma	Preclinical	^306^
	Formate–Nrf2–CD8^+^ T	Exercise induces gut microbiota remodeling→increased formate production→activates CD8^+^ T-cell Nrf2 signaling→enhances antitumor function→improves ICB efficacy	Microbiome intervention/formate supplementation/exercise intervention	Solid tumors	Preclinical (Animal)	^312^
	*Enterocloster*–MAdCAM-1–α4β7^+^CD4^+^ Treg	Antibiotic-induced *Enterocloster* colonization→downregulation of MAdCAM-1 expression in the ileum→α4β7^+^CD4^+^ Treg cell migration into the TME→inhibits anti-PD-1 immune efficacy	FMT, IL-17A neutralizing antibody, block α4β7 pathway	Solid tumors	Preclinical+Patient cohort retrospective analysis	^317^
	Vitamin D–*Bacteroides fragilis*–antitumor immunity	Vitamin D acts on intestinal epithelial cells→changes microbiota composition ↑ *B. fragilis*→enhances ICB therapy responses and anticancer immunity	Vitamin D supplementation/microbiome intervention	Solid tumors	Preclinical+Population correlation analysis	^313^
	NAM–IFN-I signaling axis–immune rebuilding	NAM supplementation activates tumor cell IFN-I signaling and immune cell function→delays M/D-induced breast cancer progression and improves ICB treatment response	Nicotinamide nutritional intervention	HR^+^HER2^-^ breast cancer	Preclinical	^314^
	Microbiome 3-IAA–myeloid peroxidase–ROS–autophagy inhibition axis	*Clostridium*, etc., produce 3-IAA→inhibits GPX3/GPX7 → ROS accumulation+autophagy inhibition→enhances PDAC chemotherapy sensitivity	3-IAA oral supplementation/tryptophan-enriched diet	PDAC	Preclinical+Population correlation analysis	^315^
	*L. reuteri*–I3A–AhR–CD8^+^IFNγ^+^ T cells–antitumor axis	Probiotic *L. reuteri* colonization in tumors→releases I3A→activates AhR signaling in CD8^+^ T cells→promotes IFN-γ secretion and enhances ICB therapy efficacy	Probiotic *L. reuteri* supplementation + tryptophan diet + ICB combination therapy	Melanoma (ICB combination)	Preclinical+Patient survival correlation analysis	^316^
	Butyrate–IL-12–ID2–CD8^+^ T	Butyrate activates the IL-12 pathway to promote ID2 expression, enhancing the cytotoxic function of CD8^+^ T cells, thereby improving the efficacy of oxaliplatin therapy	Butyrate supplementation/Dietary intervention	Oxaliplatin chemotherapy combined with CD8^+^ T-cell-related cancer treatment	Preclinical+Population association analysis	^307^
	*Roseburia intestinalis*–Butyrate–TLR5–NF-κB–CD8^+^ T	*R. intestinalis* produces butyrate, which activates the NF-κB signaling in CD8^+^ T cells through the TLR5 receptor, inducing GzmB^+^IFNγ^+^TNFα^+^ CD8^+^ T cells that inhibit tumor growth and enhance anti-PD-1 efficacy	Supplementation with R. intestinalis or Butyrate	MSI-low CRC + anti-PD-1 treatment enhancement	Preclinical+Population data	^367^
	TMAO–IFN-I–M1-like TAM–T cells	TMAO activates the immune microenvironment through the IFN-I pathway, enhancing the efficacy of ICB therapies (PD-1/TIM-3)	TMAO + ICB (anti-PD1, anti-TIM3)	PDAC, Melanoma	Preclinical+Patient microbiome prediction model	^318^

*AKR1D1* Aldo-keto reductase family 1 member D1, *NK* Natural killer cell, *LCA* Lithocholic acid, *CXCL16* C-X-C motif chemokine ligand 16, *CXCR6* C-X-C motif chemokine receptor 6, *NKT* Natural killer T cell, *IFN-γ* Interferon-gamma, *CRC* Colorectal cancer, *GPR43* G protein-coupled receptor 43, *cAMP* Cyclic adenosine monophosphate, *CD8* Cluster of differentiation 8, *FOXO1* Forkhead box O1, *Nrf2* Nuclear factor erythroid 2–related factor 2, *ICB* Immune checkpoint blockade, *MAdCAM-1* Mucosal addressin cell adhesion molecule-1, *α4β7* Integrin α4β7, Treg Regulatory T cell, *TME* Tumor microenvironment, *PD-1* Programmed cell death protein 1, *FMT* Fecal microbiota transplantation, *IL-17A* Interleukin-17A, *NAM* Nicotinamide, *IFN-I* Type I interferon, *M/D* Maintenance/Deprivation, *HR+* Hormone receptor-positive, *HER2-* Human epidermal growth factor receptor 2-negative, *3-IAA* 3-Indoleacetic acid, *GPX3/7* Glutathione peroxidase 3/7, *ROS* Reactive oxygen species, *PDAC* Pancreatic ductal adenocarcinoma, *L. reuteri* Limosilactobacillus reuteri, *I3A* Indole-3-aldehyde, *AhR* Aryl hydrocarbon receptor, *IL-12* Interleukin-12, *ID2* Inhibitor of DNA binding 2, *TLR5* Toll-like receptor 5, *NF-κB* Nuclear factor kappa-light-chain-enhancer of activated B cells, *GzmB* Granzyme B, *TNFα* Tumor necrosis factor alpha, *MSI* Microsatellite instability, *TMAO* Trimethylamine N-oxide, *TIM-3* T cell immunoglobulin and mucin-domain containing-3

SCFAs represent the most extensively characterized microbiome-derived immunoregulatory metabolites. Produced through microbial fermentation of dietary fiber, butyrate, propionate, and acetate exert pleiotropic effects on immune homeostasis.^303^ Among them, butyrate displays pronounced context-dependent activity. At physiological concentrations, butyrate functions as a potent HDAC inhibitor, promoting histone hyperacetylation and stabilizing Foxp3 expression in Tregs, thereby enforcing immune tolerance in the gut and maintaining epithelial barrier integrity.^304^ This epigenetic mechanism constitutes a direct molecular link between microbial metabolism and host immune programming. In parallel, SCFAs signal through G protein-coupled receptors, including GPR43 (FFAR2) and GPR109A (HCAR2), to modulate cytokine production in macrophages and DCs and to enhance IL-10-mediated anti-inflammatory responses.^19,305^ In cancer contexts, microbiota-derived butyrate has been shown to exert immunostimulatory effects, as exemplified by butyrate-GPR43 signaling, which increases intracellular cAMP and enhances CD8^+^ T-cell cytotoxicity in CRC models, supporting butyrate supplementation strategies in preclinical settings.^304^ Microbiota-derived butyrate promotes a forkhead box O1 (FOXO1)-induced stemness program and preserves CD8^+^ T-cell immunity against melanoma.^306^ Additional evidence indicates that butyrate can engage the IL-12-inhibitor of DNA binding 2 (ID2) axis in CD8^+^ T cells,^307^ thereby enhancing cytotoxic programs and improving responses to oxaliplatin-based chemotherapy. These seemingly paradoxical effects reflect the dual role of SCFAs, mediated through separate epigenetic and receptor signaling pathways, in regulating both immune homeostasis and effector functions, depending on tissue context, concentration gradients, and immune activation status.

Beyond SCFAs, microbiota-controlled bile acid metabolism constitutes another major MIC-III pathway. Primary bile acids synthesized by the liver are converted by intestinal microbes into secondary bile acids, including deoxycholic acid and lithocholic acid derivatives.^308^ These metabolites function as signaling ligands for nuclear receptors such as farnesoid X receptor (FXR) and membrane receptors such as GPBAR1 (TGR5), thereby linking microbial metabolism to host lipid handling and inflammatory control.^309^ Dysregulation of bile acid composition has profound immunological consequences. For instance, aldo-keto reductase family 1 member D1 (AKR1D1) deficiency-driven dysbiosis promotes the accumulation of iso-lithocholic acid (iso-LCA), which impairs NK cell cytotoxicity in liver cancer models, motivating spironolactone-based targeting of iso-LCA in preclinical studies.^310^ Conversely, microbiota-mediated bile acid conversion can also promote antitumor immunity gut microbial regulation of primary-to-secondary bile acid ratios increases CXCL16 expression in hepatic endothelial cells, leading to accumulation of CXCR6^+^ NKT cells and elevated IFN-γ production, thereby mediating liver-specific antitumor immunity in models of primary liver cancer and hepatic metastasis.^311^ Microbial metabolism further intersects with host immune regulation through diverse small-molecule mediators. Exercise-induced remodeling of the gut microbiome increases formate production, which activates Nrf2 signaling in CD8^+^ T cells and enhances antitumor function, improving ICB efficacy in preclinical solid tumor models.^312^ Similarly, dietary and microbiome-associated vitamin D signaling reshapes gut microbial composition, particularly enriching *Bacteroides fragilis*, and correlates with enhanced responses to ICB therapy across multiple tumor types.^313^ Nicotinamide (NAM), a microbiome-influenced NAD^+^ precursor, has also emerged as an immunometabolic regulator; NAM supplementation activates tumor cell IFN-I signaling and enhances immune cell function, delaying mammary tumor progression and improving ICB responsiveness in hormone receptor-positive breast cancer models.^314^ Therefore, bile acids are not uniformly immunosuppressive or immunostimulatory. Their immunological impact is determined by whether the microbiota-shaped bile acid profile favors an FXR/TGR5-mediated metabolic tolerance program or an immunostimulatory state characterized by chemokine induction and innate-like lymphocyte recruitment.

Emerging evidence further reveals that microbiome-derived metabolites can directly modulate therapy sensitivity through redox and autophagy control. In pancreatic ductal adenocarcinoma (PDAC), bacterial production of 3-indoleacetic acid (3-IAA) inhibits GPX3/GPX7 activity, leading to ROS accumulation and autophagy suppression, thereby enhancing chemotherapy sensitivity; this has prompted interest in 3-IAA supplementation or tryptophan-enriched diets.^315^ In parallel, specific probiotic species can exert localized intratumoral effects. Colonization with Lactobacillus reuteri enables intratumoral release of indole-3-aldehyde (I3A), which activates AhR signaling in CD8^+^ T cells, enhances IFN-γ secretion, and improves ICB efficacy in melanoma models.^316^ Similarly, Roseburia intestinalis-derived butyrate activates NF-κB signaling in CD8^+^ T cells via TLR5, inducing GzmB^+^IFNγ^+^TNFα^+^ cytotoxic T-cell programs and enhancing anti-PD-1 efficacy in microsatellite-stable colorectal cancer.^307^ Importantly, microbiome-immune interactions can also negatively regulate immunotherapy outcomes. Antibiotic-induced overcolonization with enterocloster suppresses mucosal addressin cell adhesion molecule-1 (MAdCAM-1) expression in the ileum, facilitates α4β7^+^CD4^+^ Treg migration into tumors, and impairs anti-PD-1 efficacy, highlighting microbiota-dependent immune trafficking checkpoints that can be modulated through fecal microbiota transplantation (FMT), IL-17A neutralization, or α4β7 pathway blockade.^317^ Finally, microbial metabolites such as trimethylamine N-oxide (TMAO) have been shown to activate IFN-I signaling, promote M1-like TAM polarization, and enhance T-cell-mediated antitumor immunity, thereby synergizing with PD-1 or TIM-3 blockade in PDAC and melanoma.^318^

Conceptually, MIC-III reveals a fundamental biological principle antitumor immunity is not governed solely by tumor-intrinsic metabolism but is continuously shaped by a systemic metabolite cloud generated by the commensal microbiota. These microbiome-derived metabolites act through receptor-mediated signaling, epigenetic reprogramming, redox modulation, and immune cell trafficking control, reconfiguring immune baselines at both the local and organismal levels. As such, therapeutic strategies targeting MIC-III, including microbiome modulation, dietary or metabolite supplementation, and host receptor intervention, represent a promising yet complex frontier for overcoming immunotherapy resistance, particularly in gastrointestinal and microbiome-associated malignancies.

From a broader mechanistic perspective, MICs operate in a manner distinct from classical immune checkpoints. Whereas conventional checkpoints largely depend on discrete cell‑surface receptor‒ligand pairs with relatively focused targets and well‑defined signaling cascades, MICs exert immunoregulatory effects through multilayer metabolic reprogramming, extensive crosstalk between metabolic and signaling pathways, and durable remodeling of immune phenotypes. These properties render MIC mechanisms more complex, dynamic, and systemic, enabling tumors to evade immune surveillance through distributed metabolic constraints while simultaneously offering new therapeutic opportunities. In immunometabolic checkpoint interventions, distinct target layers map onto distinct mechanistic entry points MIC-I, MIC-II, and MIC-III correspond to checkpoint regulation via modification of canonical signaling, direct provision of metabolite-encoded suppressive signals, and systemic microbiome-mediated immune homeostasis tuning, respectively. As summarized in Tables 4–6 and illustrated in Fig. 1, these three MIC classes regulate the TME through separable molecular logics; consequently, targeting key metabolic nodes and their immune-coupled pathways may help overcome the limitations of conventional immunotherapy and provide rational combination strategies for immune-resistant tumors, including so‑called “cold” tumors.

### Key regulatory network of MICs immunoevasion mechanisms driven by metabolic reprogramming

**Fig. 5:**
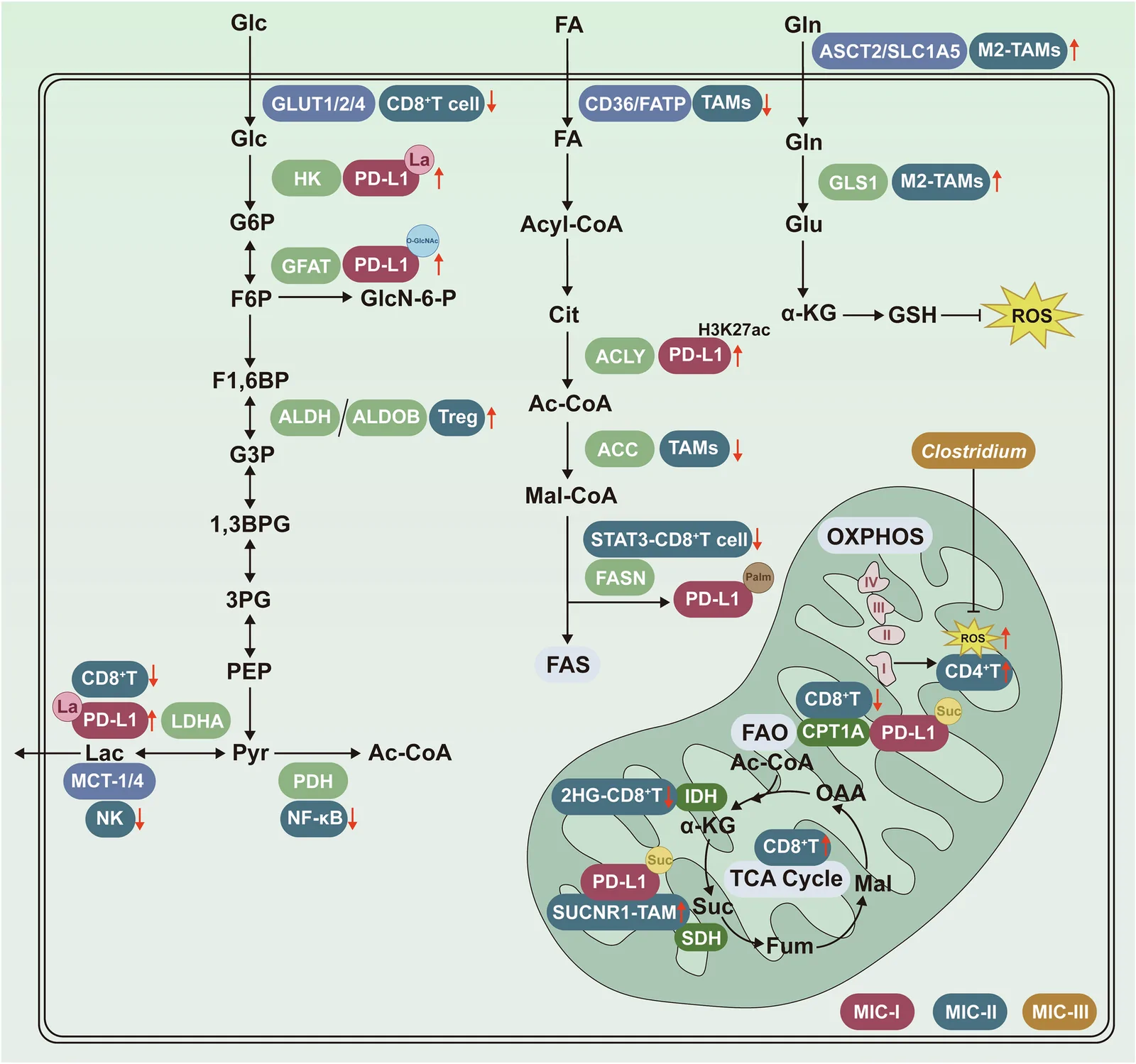
Integrated Landscape of Tumor MICs: Core Regulatory Nodes and Crosstalk. Core MICs and their crosstalk span glucose, lipid, amino acid, and mitochondrial metabolism in tumors. Key metabolic enzymes, transporters, and metabolites converge on immune checkpoint regulation, immune cell metabolic fitness, and effector function through interconnected MIC-I, MIC-II, and MIC-III mechanisms. Coordinated metabolic rewiring and interaxis communication collectively shape immune suppression, immune escape, and therapeutic responsiveness within the TME. Glc: Glucose; G6P: Glucose 6-phosphate; F6P: Fructose 6-phosphate; F1,6BP: Fructose 1,6-bisphosphate; G3P: Glyceraldehyde 3-phosphate; 1,3BPG: 1,3-Bisphosphoglycerate; 3PG: 3-Phosphoglycerate; PEP: Phosphoenolpyruvate; Pyr: Pyruvate; Lac: Lactate; Ac-CoA: Acetyl-CoA; FA: Fatty acid; Acyl-CoA: Fatty acyl coenzyme A; Cit: Citrate; Mal-CoA: Malonyl-CoA; FAS: Fatty acid synthesis; Gln: Glutamine; Glu: Glutamate; α-KG: α-Ketoglutarate; FAO: Fatty acid β-oxidation; Suc: Succinate; Fum: Fumarate; Mal: Malate; OAA: Oxaloacetate; GSH: Glutathione; GLUT1/2/4: Glucose transporter type 1/2/4; HK: Hexokinase; PD-L1: Programmed death-ligand 1; PFK: Phosphofructokinase; ALDH: Aldehyde dehydrogenase; ALDOB: Aldolase, fructose-bisphosphate B; Treg: Regulatory T cell; GAPDH: Glyceraldehyde-3-phosphate dehydrogenase; PGK-1: Phosphoglycerate kinase 1; LDH: Lactate dehydrogenase; PK: Pyruvate kinase; NK cell: Natural killer cell; MCT1/4: Monocarboxylate transporter 1/4; PDH: Pyruvate dehydrogenase; NF-κB: Nuclear factor kappa-light-chain-enhancer of activated B cells; CD36: Cluster of differentiation 36; FATP: Fatty acid transport protein; ACS: Acyl-CoA synthetase; ACLY: ATP citrate lyase; ACC: Acetyl-CoA carboxylase; FASN: Fatty acid synthase; TAM: Tumor-associated macrophage; STAT3: Signal transducer and activator of transcription 3; SLC1A5: Solute carrier family 1 member 5; GLS: Glutaminase; GDH: Glutamate dehydrogenase; ROS: Reactive oxygen species; OXCT1: 3-Oxoacid CoA-transferase 1; ARG1: Arginase 1; OXPHOS: Oxidative phosphorylation; CPT1A: Carnitine palmitoyltransferase 1A; FAO: Fatty acid oxidation; 2HG: 2-Hydroxyglutarate; IDH: Isocitrate dehydrogenase; MDH: Malate dehydrogenase; TCA cycle: Tricarboxylic acid cycle; SUCNR1: Succinate receptor 1; SDH: Succinate dehydrogenase; FH: Fumarate hydratase; MIC-I: Metabolic immune checkpoint type I; MIC-II: Metabolic immune checkpoint type II; MIC-III: Metabolic immune checkpoint type III

Tumor metabolic reprogramming not only serves as a core strategy for maintaining rapid proliferation and survival advantages but also helps tumor cells escape immune surveillance by regulating immune cell functions through the formation of MICs. Recent studies have shown that glucose, lipid, amino acid metabolism, and mitochondrial dysfunction can all regulate MICs to create an immune-suppressive microenvironment. This section systematically analyzes the critical roles of various MICs in tumor immune evasion across these major metabolic pathways (Fig. 5).

#### Glycolytic reprogramming driving immune evasion

Abnormal glycolysis is one of the hallmark features of tumor cell metabolic remodeling, particularly the Warburg effect. Even in the presence of oxygen, certain tumor cells preferentially rely on glycolysis to produce lactate. This effect not only provides energy and metabolic intermediates for rapid tumor cell proliferation but also suppresses immune responses on multiple levels, promoting immune evasion.^319^ By reshaping the metabolic landscape of the TME, enhanced glycolysis creates a competitive disadvantage for infiltrating immune cells that rely on oxidative metabolism for effector functions. For example, lactate, a key byproduct of glycolysis, significantly enhances the stability of PD-L1 through histone H3K18 lactylation,^253,320^ forming a typical MIC-I regulatory pathway. This posttranslational modification links metabolic byproducts directly to immune checkpoint reinforcement, illustrating how metabolic flux can be converted into immune inhibitory signals. Additionally, lactate accumulation mediated by LDHA suppresses the antigen-presenting function of DCs and the cytotoxic activity of CD8^+^ T cells,^239,242^ forming a potent MIC-II immune suppression axis. Meanwhile, the PKM2/HIF-1α metabolic axis has also been shown to accelerate glucose flux,^321^ leading to T-cell exhaustion^286^ and exacerbating immune tolerance. PKM2 further acts as a metabolic sensor that couples glycolytic activity to transcriptional programs controlling immune-related genes. Lactate also weakens DC activity by activating the GPR81 signaling pathway,^322^ further consolidating glycolysis’s central role in the MIC-II mechanism.

#### Lipid metabolism-mediated immune suppression

Lipid metabolism, coupled with glycolysis, also plays a key role in regulating immune evasion. Tumor cells enhance both FAS and FAO to meet their membrane synthesis and energy storage needs, and through deep intervention in the immune system, form a complex MIC network. This dual enhancement allows tumors to maintain metabolic flexibility while simultaneously shaping immune cell phenotypes within the TME. For example, FASN-mediated FAS not only promotes the palmitoylation of CD47^323^ but also enhances its expression on the cell membrane, inhibiting macrophage phagocytic activity and exhibiting a typical MIC-I mechanism. Such lipid-dependent posttranslational modification stabilizes immune checkpoint signals at the membrane level, reinforcing “don’t eat me” signaling. CD36, an important transporter for lipid uptake, is coexpressed with PD-1 to synergistically enhance Treg function,^324,325^ constructing the MIC-II immune evasion pathway. Furthermore, tumor-induced cholesterol accumulation promotes CD8^+^ T-cell dysfunction, accompanied by metabolic reprogramming of the LXR and SREBP2 signaling axes, leading to T-cell functional impairment.^294^ These alterations bias immune cells toward a dysfunctional or suppressive state rather than effective antitumor immunity. This process highlights the ability of lipid metabolism to reprogram immune cell fate and underscores the regulatory advantages of MIC-II in immune imbalance.

#### Amino acid metabolic reprogramming of the immune microenvironment

Abnormal reprogramming of amino acid metabolism, particularly the tryptophan metabolic pathway, has been repeatedly shown to play a crucial role in shaping the tumor immune microenvironment. IDO1 and TDO2 catalyze the conversion of tryptophan (Trp) into kynurenine, which activates the AhR signaling pathway, directly inducing Treg differentiation and promoting the release of immunosuppressive factors such as IL-10. This process suppresses antitumor immune responses by inhibiting IFN-β signaling^326^ and constitutes a typical MIC-II pathway driven by direct immunosuppressive signaling. In parallel, Trp depletion itself imposes a strong metabolic constraint on immune cells: under low Trp conditions, the GCN2-dependent integrated stress response is activated, leading to eIF-2-mediated translational repression, cell cycle arrest, and T-cell exhaustion while simultaneously inhibiting the mTOR signaling axis and inducing autophagy.^85,213,214^ This nutrient deprivation-driven stress response represents a “metabolic starvation” form of MIC-II, acting at a fundamental cellular physiological level rather than through classical receptor‒ligand interactions. Collectively, these mechanisms demonstrate that IDO1 functions as a central metabolic checkpoint that integrates nutrient competition, metabolite-mediated nuclear signaling, and stress-response pathways, thereby coordinating multiple layers of MIC-II regulation. Moreover, other amino acids, such as arginine and glutamine, follow similar principles: arginase- and glutaminase-mediated catabolism generates distinct MIC networks that further restrict the metabolic flexibility and effector function of immune cells, underscoring that amino acid metabolism has evolved beyond simple “nutritional support” to become a core determinant of immune suppression and microenvironmental balance.

#### Mitochondrial dysfunction and immune tolerance

Tumor cells further expand their immune evasion strategies by disrupting mitochondrial homeostasis in immune cells. As the central hub of cellular energy production and metabolic integration, mitochondria critically determine immune cell activation, differentiation, and survival. GLS1-mediated glutamine metabolism generates a large amount of ammonia (NH_3_), which accumulates in mitochondria, causing mitochondrial damage. Damaged mitochondria cannot be efficiently cleared by the autophagy pathway, leading to the death of activated effector CD8^+^ T cells and further disrupting antitumor immunity,^327^ a process that represents a typical MIC-II mechanism targeting immune cell metabolic organelles. Additionally, succinate accumulation in LPS-activated macrophages stabilizes HIF-1α expression, contributes to excessive mitochondrial ROS (mtROS) production, and activates immunity.^328^ Such “oncometabolites” act as signaling intermediates linking mitochondrial dysfunction to transcriptional and epigenetic reprogramming of immune cells. Notably, mitochondrial fragmentation, mediated by the large GTPase dynamin-related protein 1 (DRP1),^329^ alters Th17 cell effector functions.^330^ Furthermore, mtDNA released by tumor cells activates the cGAS-STING pathway in suppressive polymorphonuclear myeloid-derived suppressor cells (PMN-MDSCs), driving systemic immune suppression,^331^ a process that also falls under MIC-II but highlights the dual immunomodulatory nature of innate immune sensing pathways. Collectively, these findings indicate that mitochondria function not only as metabolic targets of immune suppression but also as platforms integrating metabolism, innate sensing, and immune tolerance.

#### Other key metabolic pathways

In addition to glucose, lipids, and amino acids, other metabolic pathways also play significant roles in tumor immune regulation. For instance, nucleotide metabolism supports the high proliferation state of tumor cells,^332^ with key enzymes such as DHODH in pyrimidine synthesis^333^ and inosine monophosphate dehydrogenase 2 (IMPDH2) in purine synthesis^334^ identified as potential tumor therapeutic targets. These enzymes indirectly function as metabolic enzyme-based MIC-II nodes by restricting nucleotide availability for rapidly proliferating immune cells. One-carbon metabolism (1CM), including the folate cycle, methionine cycle, and transsulfuration pathways, plays a critical role in energy production, nucleotide biosynthesis, DNA and RNA methylation, histone modification, and redox homeostasis regulation. By supplying methyl donors for DNA, RNA, and histone methylation, 1CM establishes a direct molecular bridge between metabolism and MIC-I-type epigenetic immune regulation, thereby influencing T-cell fate and immunotherapy responsiveness.^335^ Additionally, under low glucose conditions, tumor cells often rely on ketone metabolism for energy, where β-hydroxybutyrate, although a substitute fuel,^298^ has been found to inhibit CD8^+^ T-cell cytokine secretion and cytotoxicity.^336^ Last, microbiome-associated metabolism is increasingly recognized as a critical external regulator of tumor immunity.^337^ Gut microbiota-derived metabolites, such as SCFAs, including butyrate, can modulate immune homeostasis through G protein-coupled receptors (e.g., FFAR2/GPR43) or epigenetic mechanisms.^304^ This host-microbiota metabolic crosstalk represents a classic MIC-III axis, extending metabolic immune regulation beyond the TME to the systemic level.

In summary, major metabolic pathways, including glycolysis, lipid metabolism, amino acid metabolism, and mitochondrial function, do not operate as isolated sources of immune suppression. Instead, they serve as integrated platforms that generate, coordinate, and amplify distinct types of MICs (MIC-I, MIC-II, and MIC-III). Through extensive crosstalk within and across pathways, these MICs collectively form a multilayered and highly interconnected immunosuppressive network. For example, lactate produced by glycolysis can enhance PD-L1 stability through posttranslational modification (MIC-I), directly suppress the functions of T cells and dendritic cells (MIC-II), and may also indirectly influence the MIC-III axis by altering the metabolic state of the gut microbiota. Therefore, a deep understanding of the organizational architecture and cooperative logic of MICs at the network level is essential for achieving precise metabolic-immune interventions. Future strategies in metabolic immunotherapy should move beyond targeting single immune checkpoint molecules. Instead, they should focus on disrupting key metabolic pathways as platform-level vulnerabilities or employ multitarget combination approaches to simultaneously intervene at multiple critical nodes within the immunometabolic network. Such strategies could more systematically and durably reverse tumor-driven metabolic immune suppression, offering new theoretical frameworks and practical paths to overcome current limitations of immunotherapy, especially in the treatment of “cold” tumors.

### Organ-specific metabolic microenvironment and dominant MIC pathways

Tumors did not develop in a uniform culture dish. Distinct organ-specific physiological contexts, including perfusion patterns, oxygen gradients, basement membrane and extracellular matrix properties, stromal composition, local nutrient availability, and resident microbiota, collectively shape highly specialized metabolic niches during development and homeostasis.^338^ These organ-defined metabolic landscapes impose persistent evolutionary selection pressures on tumor cells and fundamentally determine the metabolic reprogramming strategies required for survival.^339^ In turn, such metabolic adaptations sculpt organ-tailored networks of MICs, ultimately producing organ-specific immunosuppressive phenotypes. Decoding this causal chain, from organ niche physiology to metabolic adaptation and then to immune regulation, provides a key conceptual foundation for precision metabolic immunotherapy.

#### Liver: anabolic dominance in a nutrient-rich niche

As a metabolic hub, the liver receives dual blood supply (portal vein and hepatic artery)^340^ and is chronically exposed to glucose-, amino acid-, and lipid-rich blood.^341^ This nutrient-enriched niche drives a strongly anabolic phenotype in HCC: tumor cells commonly upregulate glucose and glutamine transporters (e.g., GLUT1 and ASCT2) to maximize substrate uptake^342^; they maintain an active Warburg program even under normoxia, rapidly converting glucose into lactate and biosynthetic precursors. De novo lipogenesis is markedly activated, with increased ACLY and FASN, channeling excess acetyl-CoA into the membrane and signaling lipids.^343^ This metabolic program gives rise to an immunosuppressive network dominated by glycolysis/lipogenesis-associated MICs, in which MIC-II (metabolic-effector and nutrient-deprivation mechanisms) and MIC-I (metabolite-driven checkpoint stabilization/modification) are both prominent. Excess lactate not only acidifies the microenvironment (pH can fall below 6.5) and directly impairs T and NK cell metabolism and effector functions^344^ (MIC-II) but also amplifies suppression via multiple signaling routes: (i) uptake through monocarboxylate transporters inhibits the mTOR axis and reduces IFN-γ production in T cells^345^ (MIC-II); (ii) histone and PD-L1 lactylation enhances PD-L1 stability and inhibitory capacity^346^ (a prototypic MIC-I); and (iii) GPR81 signaling promotes M2-like polarization of TAMs^347^ (a prototypic MIC-II). Meanwhile, tumor-driven competitive consumption of glutamine further restricts the metabolic plasticity of infiltrating T cells^348^ (MIC-II: nutrient competition/metabolic constraint), deepening immunosuppression. Accordingly, metabolic immunotherapy for liver tumors should focus on breaking this anabolic dominance. Preclinical evidence suggests that combining glycolysis inhibitors (e.g., 2-deoxy-D-glucose [2-DG]),^320^ glutaminase inhibitors (e.g., CB-839),^301^ and FASN inhibitors^109^ may not only suppress tumor growth but also relieve immunosuppression and enhance responses to PD-1/PD-L1 blockade. Given the intrinsically high metabolic activity of normal hepatocytes, hepatotoxicity and therapeutic windows must be carefully monitored.

#### Lung: metabolic flexibility under oxygenation gradients

The lung is optimized for oxygen exchange, yet abnormal tumor vasculature generates pronounced oxygenation heterogeneity, from relatively well-oxygenated edges to severely hypoxic cores.^349^ Lung tumors such as NSCLC therefore evolve strong metabolic flexibility: they preferentially engage OXPHOS in normoxic regions^350^ but rapidly shift toward glycolysis under hypoxia.^33–35^ More importantly, certain cell subpopulations, such as cancer-associated fibroblasts (CAFs), generate and release lactate through MCT4,^351^ while neighboring tumor cells take up lactate via MCT1 and oxidize it to provide energy, thereby forming a metabolic symbiosis/lactate shuttle to maintain cellular survival advantages under fluctuating oxygen and nutrient conditions. In this niche, OXPHOS/lactate-utilizing MIC programs often predominate, characterized mainly by MIC-II (metabolic-state driven immune suppression) coupled with metabolism-sensing MIC nodes (e.g., HIF-1α as a central hub). Active OXPHOS is linked to stemness and therapy resistance^352^ and can support pro-survival signaling through moderate ROS outputs^353^; lactate shuttling forms a complex MIC network in which lactate serves as both a fuel for OXPHOS-dependent subsets and an immunosuppressive signal affecting immune cell function^354^ (MIC-II). Stabilization of HIF-1α represents a key regulatory hub: as a metabolism-sensing MIC, it coordinates glycolytic gene expression, angiogenic factor production, and broad immune-cell reprogramming (overall within a MIC-II suppressive framework). Given the strong metabolic heterogeneity of lung tumors, layered combination strategies are needed: mitochondrial inhibitors (e.g., metformin^191^ or IACS-010759^281^) to target OXPHOS-dependent subsets; MCT1/4 inhibitors (e.g., AZD3965^273^) to block lactate shuttling; and hypoxia-activated prodrugs (e.g., TH-302^355^) to selectively eliminate hypoxic-core cells. Such region-/subset-stratified approaches may more effectively overcome heterogeneity and reverse immunosuppression.

#### Gut: coevolution along the microbiota–host metabolic axis

Colorectal cancer develops within the body’s most complex microbial ecosystem, where the gut microbiota and their metabolites profoundly shape host metabolism and immune regulation.^356–358^ Tumor cells often evolve adaptive utilization of microbial metabolites. SCFAs, particularly butyrate, form luminal gradients^359^: tumors near the mucosa may use butyrate as a fuel,^360^ whereas deeper regions may preferentially exploit its HDAC-inhibitory activity to reprogram gene expression.^361^ Microbiota-modified bile acids, especially secondary bile acids, also serve as niche signaling molecules that influence inflammation and immune homeostasis.^362^ Accordingly, microbiota-metabolite collaborative MICs dominate in the gut and represent a canonical MIC-III (microbiota-metabolism-immunity axis), tightly coupled to MIC-II (metabolic suppressive signaling such as the Trp axis). The double-edged nature of butyrate illustrates this complexity: under physiological conditions, it supports tolerance by maintaining Treg programs via GPR43 signaling and HDAC inhibition,^363^ yet in tumors, this machinery may be hijacked to enforce immunosuppression (primarily MIC-III with downstream MIC-II-like effects). The tryptophan axis is particularly important: microbiota and tumor cells jointly drive the IDO1/TDO2-Kyn-AhR pathway,^364^ strongly suppressing effector T cells and promoting Treg differentiation^365^ (a prototypic MIC-II). Secondary bile acids further tune inflammatory setpoints via FXR and TGR5 signaling^366^ (within MIC-III metabolic signaling). Thus, reshaping microbiota composition and metabolite profiles offers a promising approach to colorectal metabolic immunotherapy. Strategies include pre/probiotics or FMT,^304,311,316,317^ small molecules targeting SCFA receptors/transporters,^304^ and IDO1/TDO2 inhibitors to block the Trp suppressive axis.^312,318^ These interventions can enhance antitumor immunity by modulating the gut microbiota and immune responses. Additionally, dietary interventions, such as high-fiber diets, can increase SCFA production, further improving therapeutic outcomes.^307,367^ Systematic evaluation of these strategies is essential to understand their potential and optimize colorectal cancer treatments, paving the way for more effective, microbiome-based therapies in clinical settings.

#### Brain: specialized metabolic adaptation in an immune-privileged compartment

The brain constitutes an immune-privileged microenvironment with unique metabolic features, including the selective permeability of the blood‒brain barrier (BBB),^368^ high energy demands primarily met by glucose and ketone bodies, active glutamate-glutamine cycling,^369^ and a lipid-rich environment with specialized myelin and sphingolipid composition. These physiological constraints not only define the metabolic preferences of resident cells but also shape the evolution and metabolic dependencies of tumors that arise within the brain.

Glioblastoma (GBM), the most common primary malignant brain tumor, exemplifies how brain tumors exploit and amplify the brain’s intrinsic metabolic landscape. GBM exhibits a marked “glutamine addiction,” driven by high expression of transporters such as ASCT2 to compete for this key substrate within the glutamate-rich milieu.^370^ Additionally, GBM co-opts the brain’s abundant lipid resources through de novo synthesis^371^ and scavenger receptor-mediated uptake to support rapid membrane biogenesis and oncogenic signaling.^293^ A distinct organ-specific adaptation is the establishment of metabolic symbiosis with astrocytes via gap junctions, allowing the direct transfer of metabolic intermediates such as lactate and glutamine to fuel tumor growth.^372^ Within this context, neuroimmune-metabolic crosstalk emerges as a critical regulatory layer. The brain’s resident immune cells, particularly microglia, possess unique metabolic programs influenced by neural activity and local metabolites. Tumor-driven perturbations, such as excessive glutamate release or altered lipid profiles, can reprogram microglia toward immunosuppressive phenotypes, thereby engaging MIC-II (metabolic enzyme/product-driven immunosuppression) mechanisms. For instance, ammonia produced as a byproduct of glutaminolysis can impair mitochondrial function in infiltrating T cells^373^ (MIC-II), while tumor-derived lipids can skew microglia/macrophage polarization toward a protumor state and disrupt cholesterol homeostasis in T cells^374,375^ (primarily MIC-II, with partial links to MIC-I through membrane protein stability/modification).

Moreover, the BBB itself functions as a structural and metabolic immune checkpoint (an organ-level MIC-II/barrier-associated MIC), severely limiting the infiltration of systemic immune cells and the delivery of most therapeutic agents.^376^ This creates a dual challenge for brain tumor therapy: overcoming the BBB and targeting central nervous system (CNS)-adapted metabolic vulnerabilities. Consequently, therapeutic strategies for GBM must address both barriers. Promising directions include developing BBB-penetrant inhibitors targeting key metabolic nodes such as glutaminase or FASN, interventions aimed at disrupting the metabolic symbiosis between tumor cells and astrocytes (e.g., via gap junction modulators), and employing transient BBB-opening technologies to enhance drug delivery. Integrating an understanding of neuroimmune-metabolic interactions will be essential for designing effective precision therapies for brain tumors.

#### Breast: stromal-epithelial metabolic crosstalk under hormonal control

Breast tumors develop within adipocyte-rich stroma and are strongly shaped by hormonal signaling, with pronounced metabolic crosstalk between tumor cells and surrounding adipocytes.^116^ Breast cancer displays subtype-specific metabolism: triple-negative/basal-like tumors tend to rely on glycolysis,^377^ whereas hormone receptor-positive/luminal tumors preferentially engage OXPHOS and FAO.^378^ Bidirectional metabolic flux is common: adipocytes supply fatty acids for tumor β-oxidation, while tumor-derived metabolites such as lactate can be utilized by adipocytes,^379^ forming local metabolic reciprocity. Consequently, an FAO/glycolysis hybrid MIC network is frequently observed, dominated mainly by MIC-II (substrate-supply coupled immune suppression) and in certain contexts accompanied by MIC-I (checkpoint homeostasis/modification). Fatty acid binding protein 4 (FABP4) is a key mediator of adipocyte-tumor metabolic coupling^380^; its inhibition can reduce fatty acid transfer and may enhance antitumor immunity (primarily targeting MIC-II substrate-supply chains). Estrogen signaling not only drives proliferation but also indirectly shapes the metabolic-immune milieu by regulating metabolic enzyme expression^381^ (within an MIC-II reprogramming framework); lactate metabolism is also tightly linked to TAM polarization^50^ (MIC-II), reinforcing local immunosuppression. Therefore, metabolic immunotherapy for breast cancer should be subtype-stratified: glycolysis inhibition may synergize with immune checkpoint blockade in triple-negative disease^382–384^ (cotargeting MIC-II + MIC-I), whereas FAO/OXPHOS targeting (e.g., CPT1-related pathways) may be more effective in hormone receptor-positive tumors^385^ (primarily MIC-II). Disrupting adipocyte-tumor interactions (e.g., FABP4 inhibition) shows preclinical promise,^386^ and the long-term metabolic remodeling induced by endocrine therapy should be incorporated into rational combination design.

#### Other organs: metabolic immune suppression shaped by extreme niches and hormone dependence

Beyond the representative organs discussed above, many other solid tumors also evolve distinct metabolic adaptation strategies within highly specialized organ-specific metabolic niches, thereby shaping immunosuppressive networks predominantly centered on MIC-II, with auxiliary contributions from MIC-I or organ-specific regulatory axes.

PDAC represents a tumor type that develops within an extremely nutrient-deprived, poorly perfused, hypoxic, and highly fibrotic microenvironment. Dense desmoplastic stroma and disrupted tumor-associated vasculature create a milieu characterized by nutrient scarcity, hypoxia, and acidosis, which not only suppresses the function of antitumor immune cells such as CD8^+^ T cells but also promotes resistance to chemotherapy.^387^ To survive in this supply-restricted niche, PDAC cells have evolved a scavenger-like metabolic strategy centered on macropinocytosis and autophagy-lysosomal pathways. Through macropinocytosis, PDAC cells internalize extracellular proteins and degrade them in lysosomes to replenish amino acids while maintaining high basal autophagy to recycle intracellular components.^388^ In parallel, metabolic crosstalk enables stromal cells, particularly pancreatic stellate cells, to supply key metabolites such as alanine, establishing a prominent stroma-driven nutrient supply axis.^389^ Collectively, this adaptive mode, characterized by sustained nutrient competition and metabolic suppression of immune cells, constitutes a typical MIC-II (nutrient deprivation/metabolic dependency driven immune suppression) framework. Moreover, enhanced autophagy and lysosomal activity may indirectly impair antigen presentation, further reinforcing immune evasion. Notably, PDAC also exhibits alternative biosynthetic dependencies shaped by the metabolic constraints of its microenvironment. Arginine is frequently depleted in the PDAC TME, but tumor growth requires high levels of polyamines. Recent studies have shown that PDAC relies on de novo synthesis of ornithine from glutamine to sustain polyamine production. This dependency is closely linked to oncogenic KRAS, which induces the expression of ornithine aminotransferase (OAT) and polyamine biosynthetic enzymes, accompanied by extensive remodeling of the transcriptome and chromatin accessibility landscape.^390^ Through this KRAS-driven metabolic rewiring, PDAC cells maintain proliferation and malignant behaviors despite persistent arginine scarcity within the TME. Prostate cancer, in contrast, illustrates a hormone-driven mode of metabolic-immune reprogramming.^391^ Under androgen receptor (AR) signaling, prostate cancer cells undergo a metabolic switch from the citrate-secreting phenotype characteristic of normal prostate epithelium to citrate oxidation, redirecting citrate into the TCA cycle to support energy production and biosynthesis.^392^ Concurrently, AR signaling markedly enhances de novo fatty acid and cholesterol synthesis to sustain membrane architecture, lipid raft formation, and oncogenic signaling.^393^ This lipid- and cholesterol-centered adaptation suppresses antitumor immunity both by altering immune cell membrane composition and signaling thresholds (MIC-II) and by modulating immune checkpoint protein localization and stability (MIC-I), together establishing a tightly coupled metabolic-endocrine-immune suppressive network.

Bone tumors represent a distinct paradigm of metabolic immune suppression shaped by a mineralized, mechanically rigid, and dynamically remodeled niche.^394^ Primary bone malignancies and bone metastases from breast and prostate cancer develop within a microenvironment characterized by high calcium and phosphate flux, hypoxia in the marrow cavity, and continuous osteoblast-osteoclast coupling,^395^ collectively imposing strong metabolic constraints that favor MIC-II dominant immune suppression. Osteoclast-driven bone resorption releases calcium, TGF-β, and matrix-derived nutrients.^396^ Elevated extracellular Ca^2+^ suppresses immune effector cell function via ionic imbalance and activates the Ca^2+^-TFEB (transcription factor EB)-LDHB axis to promote lactate oxidation and metabolic reprogramming,^397^ collectively shaping an MIC-II-dominated immunosuppressive niche under hypoxic conditions. In parallel, metabolic coupling between tumor cells, osteoclasts, and myeloid populations sustains glucose and amino acid deprivation, reinforcing immunosuppressive phenotypes such as MDSCs and tolerogenic macrophages. In addition, adenosine generated from ATP metabolism during bone matrix degradation accumulates locally^398^ and further suppresses T-cell and NK cell activity, partially overlapping with MIC-I type immune checkpoint regulatory mechanisms. In hormone-dependent bone metastases, androgen- or estrogen-driven reprogramming of lipid and cholesterol metabolism further integrates endocrine, metabolic, and immune regulatory networks,^399^ amplifying immune suppression through substrate competition and alterations in osteoblast-osteoclast balance. Collectively, bone tumors illustrate how the skeletal niche, characterized by mechanical rigidity, high mineral content, and continuous remodeling, shapes an immunosuppressive network centered on MIC-II, with superimposed bone-specific mechanisms including calcium signaling, purinergic pathways, and remodeling-associated metabolite release.

Beyond these paradigms, other organ-specific tumors also exhibit distinct metabolic immune suppression patterns. For example, renal cell carcinoma (RCC) develops within a relatively oxygenated renal environment yet displays profound metabolic reprogramming driven by the von Hippel‒Lindau tumor suppressor (VHL)-HIF axis, leading to abnormal lipid and glycogen accumulation and immune suppression mediated largely by HIF-dependent metabolic remodeling and lactate accumulation^400–403^ (MIC-II). Ovarian cancer evolves in a lipid-rich peritoneal milieu and frequently acquires a strong dependence on fatty acid uptake and oxidation,^404,405^ promoting immune cell dysfunction primarily through substrate-driven MIC-II mechanisms. Melanoma, despite its high intrinsic immunogenicity, resides in the skin, a tissue characterized by oxidative stress and metabolic fluctuations,^406^ and often establishes a suppressive landscape in which MIC-II and MIC-I coexist, involving tryptophan metabolism, enhanced oxidative phosphorylation, and immune checkpoint homeostasis. Collectively, these other organ tumors further reinforce a central principle: organ-specific metabolic niches determine accessible metabolic resources, and the modes of resource acquisition ultimately define the dominant MIC architecture. In contrast to gut tumors where microbiota participation renders MIC-III dominant, most visceral organ tumors are primarily governed by MIC-II (metabolic dependency and nutrient competition-driven immune suppression), with superimposed organ-specific mechanisms such as hormonal signaling, physical barriers, or metabolite-mediated checkpoint regulation (MIC-I). These insights underscore that metabolic immunotherapy must integrate tumor type, organ origin, and metabolic niche rather than targeting isolated molecules or pathways.

**Fig. 6:**
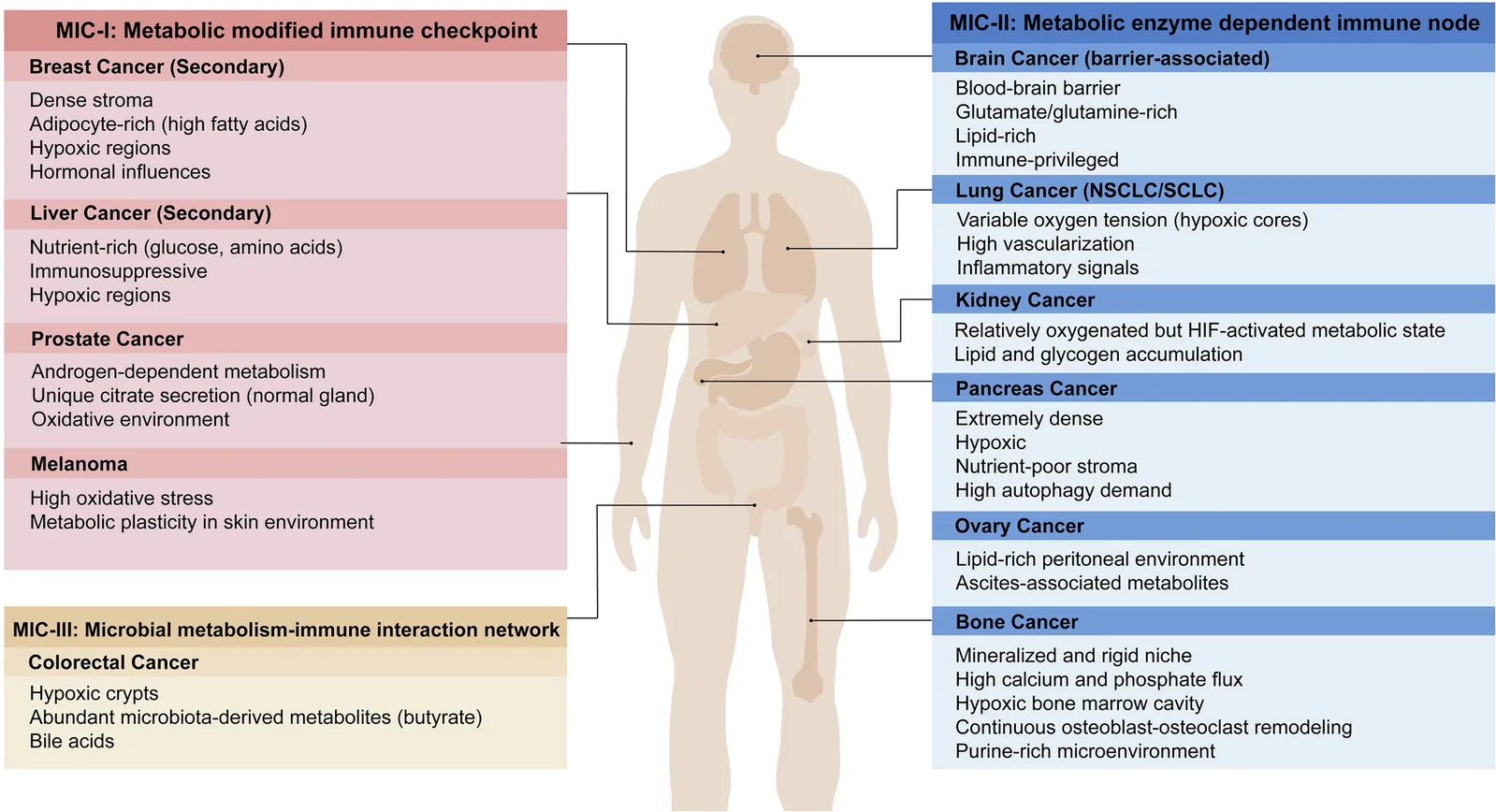
Organ-specific Architectures of MICs Across Tumor Types. Organ-specific metabolic microenvironments shape distinct MIC architectures across major tumor types. Tissue contexts, including nutrient availability, oxygen tension, stromal composition, microbiota influence, and barrier properties, determine the predominance of MIC-I, MIC-II, or MIC-III programs. These organ-adapted MIC configurations drive unique metabolic dependencies, immune suppression mechanisms, and therapeutic vulnerabilities, highlighting the need for tissue-informed immunometabolic targeting strategies

In summary, distinct physiological metabolic environments across organs impose long-term selective pressures that shape tumor-specific metabolic adaptation strategies. These adaptations, in turn, establish organ-tailored networks of MICs, determining the structure and intensity of local immune suppression (Table 7, Fig. 6). In organs characterized by relative nutrient abundance and lipid availability, such as the liver and breast, tumor immune suppression is typically codriven by MIC-II (nutrient competition and metabolic effector suppression) and MIC-I (metabolite-mediated immune checkpoint stabilization or posttranslational modification). Lung tumors prominently display metabolic and immune heterogeneity arising from the coupling of MIC-II with metabolism-sensing MIC hubs, particularly HIF-1α. In contrast, gut tumors, deeply embedded within complex microbial ecosystems, are dominated by MIC-III (microbiota-metabolite-immune axes) in close synergy with MIC-II pathways, notably the Trp-Kyn-AhR axis. Brain tumors exhibit a highly specialized suppressive landscape shaped by MIC-II combined with barrier-associated MICs, where the BBB itself constitutes a critical organ-level metabolic-immune constraint. Extending this framework, tumors arising in other organs consistently follow the core logic of organ niche, metabolic adaptation, and dominant MIC subtype. PDAC relies on autophagy- and macropinocytosis-driven scavenging metabolism to establish MIC-II-dominated immune suppression, while prostate cancer leverages androgen-driven lipid and cholesterol reprogramming to suppress immunity through MIC-II with additional MIC-I contributions. Similarly, RCC, ovarian cancer, and melanoma predominantly engage MIC-II centered mechanisms, with organ-specific overlays involving hypoxia signaling, lipid availability, or immune checkpoint homeostasis.

**Table 7:** Summary of predominant MIC subtypes, functions, and potential interventions in primary tumors across organs

Primary Tumor Organ	Metabolic Microenvironment Traits	Predominant MIC Subtype	Key Metabolic Adaptation and Biological Effects	Potential Targeting Strategies
Liver (HCC)	Nutrient-rich (glucose, amino acids), immunosuppressive, hypoxic regions	MIC-II (dominant) + MIC-I	1. Nutrient Scavenging: High expression of GLUT1 and glutamine transporters; 2. Warburg Effect: Rapid glycolysis for biomass production even under normoxia; 3. Lipid Factory: Upregulated FASN for membrane synthesis and signaling	Target Anabolism: (1) Glycolysis inhibitors (e.g., 2-DG); (2) Glutaminase inhibitors (e.g., CB-839); (3) FASN inhibitors; Combine with chemotherapy/targeted therapy
Lung (NSCLC/SCLC)	Variable oxygen tension (hypoxic cores), high vascularization, inflammatory signals	MIC-II (dominant)	1. Metabolic Flexibility: Prefers OXPHOS in normoxic regions, switches to glycolysis in hypoxia; 2. Lactate Recycling: Uses MCT1 to import lactate as a fuel source; 3. Therapy Resistance: OXPHOS contributes to stemness and chemoresistance	Target Energy Metabolism: (1) Mitochondrial inhibitors (e.g., metformin); (2) MCT1/4 inhibitors; (3) Hypoxia-activated prodrugs
Colorectal	Hypoxic crypts, abundant microbiota-derived metabolites (butyrate), bile acids	MIC-III (dominant) + MIC-II	1. Butyrate Utilization: Uses microbial SCFAs for energy or as HDAC inhibitors; 2. Bile Acid Signaling: Altered bile acid metabolism promotes proliferation; 3. Immunosuppression: Metabolites like kynurenine inhibit T-cell function	Modulate the Microenvironment: (1) Microbiome modulation (pre/probiotics); (2) SCFA receptor/transporter targeting; (3) IDO1/TDO2 inhibitors; Combine with immunotherapy
Brain (GBM)	Blood‒brain barrier, glutamate/glutamine-rich, lipid-rich, immune-privileged	MIC-II (dominant) (barrier-associated)	1. Glutamine Addiction: High ASCT2 expression to capture neural glutamine; 2. Lipid Dependency: Relies on lipids for membrane synthesis and signaling; 3. Metabolic Symbiosis: Forms gap junctions with astrocytes for metabolite exchange	Target CNS-Specific Metabolism: (1) Glutaminase inhibitors (CB-839); (2) Lipid metabolism inhibitors (e.g., FASN/CPT1 inhibitors); (3) Disrupt symbiosis (gap junction blockers); Enhanced drug delivery systems
Breast	Dense stroma, adipocyte-rich (high fatty acids), hypoxic regions, hormonal influences	MIC-II (dominant) ± MIC-I	1. Stromal Interaction: Uses adipocyte-derived fatty acids via FAO for energy in some subtypes; 2. Metabolic Subtype Variation: Basal-like: glycolytic; Luminal: more OXPHOS/FAO; 3. Hormone-Metabolism Crosstalk: Estrogen signaling influences metabolic enzyme expression	Subtype-Specific Targeting: (1) For basal-like: Glycolysis inhibitors; (2) For luminal: FAO/OXPHOS inhibitors (e.g., etomoxir); (3) Target adipocyte-tumor interaction (e.g., FABP4 inhibitors)
Pancreas (PDAC)	Extremely dense, hypoxic, nutrient-poor stroma, high autophagy demand	MIC-II (dominant)	1. Nutrient Scavenging: Utilizes macropinocytosis to ingest extracellular proteins for amino acids; 2. Autophagy Addiction: High basal autophagy to recycle intracellular components under starvation; 3. Stroma-Driven Metabolism: Stromal cells provide alanine and other metabolites	Target Nutrient Scavenging: (1) Autophagy inhibitors (e.g., hydroxychloroquine); (2) Macropinocytosis inhibitors (in development); (3) Deplete stromal-derived nutrients
Prostate	Androgen-dependent metabolism, unique citrate secretion (normal gland), oxidative environment	MIC-II + MIC-I	1. Lipogenesis Surge: Androgen receptor signaling drives FASN and ACC expression for lipid synthesis; 2. Altered Citrate Metabolism: Shifts from citrate secretion to its oxidation for energy in cancer; 3. Cholesterol Addiction: High cholesterol synthesis for membrane fluidity and signaling	Target Lipid/Cholesterol Synthesis: (1) FASN/ACC inhibitors; (2) Statins (HMG-CoA reductase inhibitors); (3) Anti-androgen therapy (indirect metabolic impact)
Bone	Mineralized and rigid niche; high calcium and phosphate flux; hypoxic bone marrow cavity; continuous osteoblast-osteoclast remodeling; purine-rich microenvironment	MIC-II (dominant) ± MIC-I	1. Ion- and nutrient-driven immune suppression via elevated Ca^2+^/phosphate; 2. Hypoxia-adapted glycolysis and glutamine dependence causing lactate accumulation; 3. Stromal metabolic coupling with osteoclasts and myeloid cells promoting MDSCs and tolerogenic macrophages; 4. Adenosine-mediated purinergic suppression; 5. Hormone-driven lipid/cholesterol rewiring in bone metastases	Target bone-specific metabolic-immune axes: (1) Osteoclast inhibition (bisphosphonates, RANKL blockade); (2) Adenosine pathway targeting (CD39/CD73-A2A axis); (3) Modulation of hypoxia and glycolysis; (4) Hormone therapy combined with immunometabolic interventions in bone metastases
Kidney (ccRCC)	Relatively oxygenated but HIF-activated metabolic state; lipid and glycogen accumulation	MIC-II	1. VHL-HIF driven metabolic reprogramming; 2. lactate accumulation; immune suppression via hypoxia signaling	(1) HIF pathway inhibitors; (2) Glycolysis/lactate-targeting strategies; Combination with immunotherapy
Ovary	Lipid-rich peritoneal environment; ascites-associated metabolites	MIC-II	1. Enhanced FA uptake and oxidation; 2. lipid-driven immune cell dysfunction	(1) FA uptake/FAO inhibitors; (2) Metabolic-immune combination therapies
Melanoma	High oxidative stress; metabolic plasticity in skin environment	MIC-II ± MIC-I	1. Enhanced OXPHOS; 2. tryptophan metabolism; 3. immune checkpoint homeostasis modulation	(1) OXPHOS inhibitors; (2) IDO1/TDO2 inhibitors; (3) Checkpoint blockade combinations

*HCC* Hepatocellular carcinoma, *MIC-I* Metabolic immune checkpoint type I, *MIC-II* Metabolic immune checkpoint type II, *MIC-III* Metabolic immune checkpoint type III, *GLUT1* Glucose transporter type 1, *FASN* Fatty acid synthase, *2-DG* 2-Deoxy-D-glucose, *CB-839* Telaglenastat, *NSCLC* Non-small cell lung cancer, *SCLC* Small cell lung cancer, *OXPHOS* Oxidative phosphorylation, *MCT1* Monocarboxylate transporter 1, *MCT4* Monocarboxylate transporter 4, *SCFAs* Short-chain fatty acids, *HDAC* Histone deacetylase, *IDO1* Indoleamine 2,3-dioxygenase 1, *TDO2* Tryptophan 2,3-dioxygenase, *ASCT2* Alanine-serine-cysteine transporter 2, *CNS* Central nervous system, *CPT1A* Carnitine palmitoyltransferase 1A, *FABP4* Fatty acid binding protein 4, *PDAC* Pancreatic ductal adenocarcinoma, *ACC* Acetyl-CoA carboxylase, *HMG-CoA* 3-Hydroxy-3-methylglutaryl-coenzyme A, *ccRCC* Clear cell renal cell carcinoma, *VHL* Von Hippel‒Lindau tumor suppressor, *HIF* Hypoxia-inducible factor, *FA* Fatty acid

Accordingly, organ specificity mandates that effective metabolic immunotherapy be both contextualized and individualized. Beyond molecular subtyping and genetic alterations, therapeutic strategies must incorporate the metabolic niche defined by the tissue of origin and its dominant MIC architecture. Future priorities include (i) constructing high-resolution, organ-specific metabolic-immune atlases to resolve MIC network hierarchies, (ii) developing metabolic modulators and delivery systems capable of crossing key physiological barriers such as the BBB, (iii) identifying predictive biomarkers reflecting organ-specific metabolic features; and (iv) designing organ-stratified clinical trials to evaluate the most relevant metabolic-immune combination strategies. Shifting metabolic immunotherapy from a one-size-fits-all paradigm toward niche-guided precision intervention holds promise for overcoming therapeutic resistance, particularly in traditionally immunologically cold tumors, and advancing cancer treatment toward an integrated genotype-metabotype-immunotype framework.

## Clinical translation: from concept to therapeutics

### MIC drug development and associated adverse effects

Despite the enormous potential of metabolic-targeting strategies in cancer immunotherapy, their clinical translation still faces numerous challenges.^29,407–409^ Many discoveries targeting MICs have demonstrated strong immunomodulatory effects in preclinical studies, yet only a few have become effective clinical therapeutics. This highlights a persistent gap between scientific concepts and real-world application. Tables 4–6 detail the mechanisms of MIC-I, MIC-II, and MIC-III, while Table 8 reorganizes these findings by metabolic pathway. It focuses on practical treatment strategies and summarizes key clinical trials targeting MICs, reflecting the varied and ongoing progress in the field. Some metabolic drugs have made positive progress in preclinical or early clinical stages. For example, metformin, a first-line drug for treating type 2 diabetes, inhibits hepatic glucose production and has shown the ability to promote DC maturation, enhance costimulation of CD4^+^ T cells, and boost antitumor immunity.^191^ It can also enhance the cytotoxic ability of γδ T cells against cancer cells.^410^ This suggests that metformin targets MIC-II mechanisms by regulating immune cell function through metabolic products to enhance antitumor immunity. However, such broadly tolerated metabolic agents remain uncommon, and most purpose-designed metabolic inhibitors encounter substantial translational barriers. For instance, the HK2 inhibitor lonidamine blocks the first step of glycolysis and reduces ATP levels in tumors by 60%, representing an MIC-II type mechanism involving enzyme-dependent metabolic immune regulation. However, systemic delivery often causes energy loss in normal tissues, especially in high-energy organs such as the brain and heart, limiting its use. This points to a core problem: pathways overactive in tumors are often still vital for normal body function. Structural optimization is urgently needed, along with the development of TME-targeted delivery systems. Notably, metabolic inhibitor toxicity differs from ICB toxicity. ICBs mainly cause immune-related adverse effects (AEs), such as myocarditis and pneumonitis, from excessive immune activation and loss of immune tolerance. In contrast, metabolic inhibitors cause toxicity by directly disrupting essential metabolic processes in normal tissues. This distinction highlights the need for a more cautious approach in the clinical development of MIC-targeted therapies.

**Table 8:** Clinical progress of therapies targeting MICs: summary of key clinical trials

MIC type	Metabolic category	Target/Pathway	Agent/Drug	Patient population	Phase/Stage	Intervention/Combination	Key outcomes (efficacy and safety)/Status	Trial ID/Ref
MIC-I	Glucose Metabolism	PD-L1 glycosylation axis (OGT)	HGS (short peptide competitor)	Not specified	Preclinical	Competitive inhibition strategy	CPP-G1 dose-dependently lowers PD-L1, spares global O-GlcNAc; boosts T-cell cytotoxicity	^266^
		PD-L1 lactylation	Serine/glutamine-deprivation diet	CRC	I	Dietary intervention	Not report	^240^
		LDHA → PD-L1 lactylation (K162)	FX11 (LDHA inhibitor)	NSCLC, CRC	Preclinical	Pharmacologic inhibition	LDHA inhibition lowers H3K18la/H3K9la, reduces GZMB/IFNγ, and weakens CD8 killing	^239,242^
		HK2 → NF-κB → PD-L1 transcription	2-DG; Lonidamine (HK2i)	Glioma	Preclinical	HK2 inhibitor + anti-PD-1	Combination reduced PD-L1, increased CD8^+^ T-cell infiltration, and inhibited tumor growth synergistically in mice	^320^
		AMPK → PD-L1 (Ser283) and EZH2 phosphorylation	AMPK agonist/ketogenic diet + anti-CTLA-4	Solid tumors	Preclinical	Metabolic activation + ICB	Ketogenic diet or AMPK agonist synergizes with anti-CTLA-4 to inhibit tumor growth and extend survival in murine models	^450^
	Fatty Acid Metabolism	CPT1A → PD-L1 succinylation/degradation	Metabolite supplementation (DES, DMK)/metabolic inhibitors	Melanoma	Preclinical	Metabolic modulation + ICB	Enhanced tumor suppression and T‑cell activation with CPT1A upregulation	^261^
		ACLY→histone acetylation (H3K27ac)→ PD-L1 transcription	SB-204990 (ACLYi)	Lung cancer	Preclinical	ACLYi + αPD-L1; Dietary PUFAs + αPD-L1	Synergistic tumor regression, enhanced CD8^+^ T-cell function, durable immunity	^124^
		PD-L1 K189 lactylation ↔ HDAC2 delactylation; YY1 → SQLE axis	HDAC2 inhibitors	HCC	Preclinical	Combination therapy with HDAC2 inhibitor and SQLE inhibitor	Synergistically suppressed tumor growth and cholesterol synthesis in preclinical HCC models	^248^
MIC-II	Glucose Metabolism	GLUT1	BAY-876	Lung cancer, pancreatic cancer	Preclinical	With ICBs	Restored CD8^+^ T-cell function and enhanced anti-PD-1 efficacy in mice, with acceptable safety	^288^
		PFKFB3	PFK-158	Solid tumors	I	Dose-escalation study	Recruiting	NCT02044861^452^
		MCT1	AZD3965	Advanced solid, hematologic malignancies	I	Dose-escalation study	Safety: Manageable on-target ocular & cardiac toxicities. Efficacy: Limited in unselected advanced cancers. RP2D = 10 mg BD	NCT01791595^453^
		MCT4	MSC-4381	Solid tumors	Preclinical	With ICBs	Enhanced T-cell infiltration/activation, delayed tumor growth, prolonged survival, no severe toxicity	^454^
		Lactate Transport	CYT-0851	B-cell malignancies, solid tumors	I/II	With or without Gemcitabine	Completed, no results posted	NCT03997968
		LDH	Galloflavin	Breast cancer (triple-negative, tamoxifen-resistant)	Preclinical	Galloflavin monotherapy	Induces apoptosis, reduces ATP/lactate, less toxic to normal lymphocytes	^455^
		Glycolysis	Chloroquine	Breast cancer	I/II	CQ alone; CQ + low-dose cyclophosphamide; CQ + IPI-549 (PI3Kγ inhibitor)	Enhances antitumor T-cell immunity, reduces immunosuppressive cells (MDSCs, Tregs) in TME. Safe profile noted in preclinical models	^456^
			2-Deoxy-D-glucose (2-DG)	Breast cancer	I/II	Monotherapy	2-DG inhibits M2 macrophage polarization, reduces tumor growth and allergic inflammation in mice. No significant cytotoxicity at low doses	^457^
	Fatty Acid Metabolism	COX-2	Celecoxib	CRC	II	With PD-1 blocker	No results posted	NCT03638297, NCT03926338, NCT05731726
		PTGER2	PF-04418948	CRC, TNBC	Preclinical	With PD-1 blocker	Prevents cDC1 dysfunction, enhances CD8^+^ T-cell infiltration/activation, controls tumor growth	^458^
		PTGER2 + PTGER4	TPST-1495	Solid tumors	Ia/Ib	With PD-1 blocker	Completed, no results posted	NCT04344795
		PTGER4	L161,982	CRC, TNBC	Preclinical	With PD-1 blocker	Prevents cDC1 dysfunction, enhances CD8^+^ T-cell infiltration/activation, controls tumor growth	^458^
			MF-766	Breast cancer, CRC	Preclinical	With PD-1 blocker	Superior antitumor efficacy in CT26/EMT6 models; modulates myeloid and lymphocyte infiltration	^459^
		CD36	CD36 blockers	Melanoma, pancreatic cancer	Preclinical	Optionally combined with ICBs	Reduced tumor growth, enhanced antitumor immunity without autoimmunity	^325,460^
		FAO (CPT1)	Etomoxir	GBM	Preclinical	With CD47 blocker and radiotherapy	Improved disease control coupled with signs of restored immunity	^461^
		FASN	Culeenin	Ovarian cancer	Preclinical		Suppressed tumor growth, enhanced antitumor immunity in mice	^462^
			Denifanstat (TVB-2640)	Astrocytoma, HER2^+^ breast cancer, NSCLC	II	With Bevacizumab	ORR 56%, PFS 4.6 months, well-tolerated with manageable AEs	NCT03032484, NCT03179904, NCT03808558;^463,464^
		THBS1	VT1021	GBM	II/III	Compared with standard-of-care chemotherapy	Recruiting	NCT03970447
		SQLE/cholesterol	Terbinafine	Breast cancer	I/II/III	Terbinafine alone	SQLE inhibition reduces cell viability, prolongs doubling time in SQLE-amplified breast cancer cells. Copy-dosage correlated sensitivity	^465^
		LXR/ABCA1	Simvastatin	NSCLC with EMT-associated drug resistance	Preclinical	Legumain-activatable liposomal codelivery system	Reversed EMT, repolarized TAM, overcame drug resistance in xenograft models with good biocompatibility	^466^
		PPARγ	GW9662	Breast cancer	Preclinical	Coincubation with CB2 antagonist (SR144528) and/or PPARγ antagonist (GW9662)	GW9662 antagonized DHEA/EPEA-mediated reduction of IL-6 in LPS-stimulated adipocytes	^467^
		HDAC	TMP195	Breast cancer	Preclinical	TMP195 alone or combined with chemotherapy/anti-PD-1	Reduced tumor burden and metastasis by promoting phagocytic, stimulatory TAMs; enhanced efficacy of chemo/immunotherapy	^468^
			Tefinostat (CHR-2845)	Breast cancer	Preclinical	Monocyte/macrophage-targeted HDAC inhibition	Monocyte-specific HDAC inhibition with reduced systemic toxicity in preclinical models	^469^
	Amino Acid Metabolism	IDO1	Epacadostat	Melanoma, treatment-naïve	III	Epacadostat + pembrolizumab vs placebo+pembrolizumab	mPFS: 4.7 vs 4.9 months (HR = 1.00, 95% CI 0.83–1.21; P = 0.52); Grade 3-4 AEs: 32% vs 18%	NCT02752074 (ECHO-301/KEYNOTE-252)^412^
				MDS	II	Monotherapy	No objective responses; stable disease in 80% of patients; manageable safety profile	NCT01822691
				Cisplatin-ineligible urothelial carcinoma (UC)	II	Combination immunotherapy	ORR 31.8% (vs 24.5% w/placebo); no significant new safety signals	NCT03361865
				Advanced solid tumors	I/II	With durvalumab	ORR 12.9% (300 mg dose); safety as expected from combo profile	NCT02318277
				NSCLC	I	With atezolizumab	Study terminated early; no results posted	NCT02298153
				Melanoma	I/II	With ipilimumab	ORR 27-35%; no added benefit over historical ipilimumab monotherapy; development halted	NCT01604889
				Advanced cancers	III	With nivolumab + Chemotherapy	Study withdrawn before patient enrollment due to portfolio reprioritization	NCT03342352
			Navoximod (GDC-0919)	Advanced solid tumors	I	Monotherapy	No objective responses; SD in 45% of patients; manageable safety profile	NCT02048709
			LY3381916	TNBC, NSCLC	I	With or without anti-PD-L1	Study terminated early due to strategic business decision; no results posted	NCT03343613
			IU1 (USP14 inhibitor)	CRC	Preclinical	Combined with a PD-1 blocker	USP14 inhibition reduces IDO1, enhances T-cell function, and improves anti-PD-1 efficacy without AhR activation	^128^
			AT-0174	Advanced solid tumors	I	/	Recruiting	ACTRN12623000956606
		IDO pathway (Trp depletion/mTOR)	Indoximod	Melanoma	I/II	With pembrolizumab/nivolumab	Study terminated by sponsor; limited efficacy data; manageable safety profile	NCT03301636
				Pediatric progressive primary malignant brain tumors	I	Temozolomide	Well tolerated, favorable response rate	^470^
				Refractory metastatic prostate cancer	II	Sipuleucel-T	Well tolerated, significant clinical improvement	NCT01560923^471^
				Relapsed brain tumors or newly diagnosed DIPG	II	Chemotherapy and radiation	Recruiting	NCT04049669
				Pediatric brain cancer	I	Ibrutinib and chemotherapy	Recruiting	NCT05106296
		ASCT2	V-9302	CRC, lung cancer, TNBC	Preclinical	Optionally with PD-1 blocker	Synergistic antitumor effect when combined with anti-PD-L1, rescues T-cell function in vivo	^472–474^
		GLS1	Telaglenstat (CB-839)	RCC	II	Cabozantinib	No difference in PFS and ORR	NCT03428217^475–477^
				Advanced or metastatic RCC	II	Everolimus	Well tolerated, Improved PFS	NCT03163667^475^
				ccRCC, melanoma, NSCLC	I/II	Nivolumab	Lack of efficacy, no difference PFS	NCT02771626
				Solid tumors	I/II	Palbociclib	Results not available	NCT03965845
				Various solid, hematologic malignancies	I/II	CB-839 + Capecitabine	Disease control rate 50%, no objective responses. Manageable toxicity	NCT02861300
				NSCLC	I	Sapanisertib	Active, not recruiting	NCT04250545
			IPN60090 (IACS-6274)	Solid tumors	I	Monotherapy or Combo (Bev+Pac or Capiva)	Dose escalation ongoing. Safety and preliminary efficacy under evaluation	NCT05039801
			IPN60091	Advanced solid tumors	I	Bevacizumab and paclitaxel	Recruiting	NCT05039801
		LAT2	BCH	Osteosarcoma	Preclinical	With CD47 blocker	Reduced CD47, enhanced phagocytosis, suppressed tumor growth	^478^
		Arginase 1/2	OATD-02	Advanced or metastatic solid tumors	I	/	Recruiting	NCT05759923
		NOS/arginase	L-NMMA	Breast cancer	I/II	L-NMMA + docetaxel ± amlodipine	ORR 45.8%, safe, no L-NMMA-related grade ≥3 toxicity	^479^
		Glutamine	Glufosinate	Breast cancer	Preclinical	Glufosinate monotherapy	Reduced metastasis, no primary tumor growth inhibition, well-tolerated without liver/brain toxicity	^480,481^
	Mitochondrial Metabolism	PDH and OGDH	CPI-613 (devimistat)	Metastatic PDAC	III	With FOLFIRINOX	No difference in OS, PFS and OR	NCT03504423
				Acute myeloid leukemia	III	Cytarabine + mitoxantrone	Terminated (futile)	NCT03504410
				Pancreatic cancer	I	Monotherapy	AEs; three patients with increased OS	NCT07414403
				Pancreatic cancer	I	With FOLFIRINOX	Completed (not reported)	NCT01835041
				Pancreatic cancer	I/II	With gemcitabine	Terminated	NCT00907166
		mIDH1	FT-2102/olutasidenib	Solid tumors, glioma	I/II	With nivolumab/azacitidine/GemCis	Completed (not reported)	NCT03684811
			AG-221/enasidenib	Hematologic malignancies	I/II	Multiple regimens	Prolonged survival	NCT01915498
			BAY1436032	Acute myeloid leukemia	I	Monotherapy	Low ORR	NCT03127735
			Ivosidenib	mIDH1-AML	III	Azacitidine	Significant benefit on PFS	NCT03173248^482^
		mIDH1/2	Vorasidenib	Grade 2 IDH-mutant glioma	III	Monotherapy	Significant benefit on PFS	NCT04164901^483^
				Recurrent or progressive mIDH1 glioma	I	Pembrolizumab	Recruiting	NCT05484622
		ETC Complex I	IACS-010759	Advanced cancers	I	Monotherapy	High blood lactate, neurotoxicity	NCT03291938
				Acute myeloid leukemia	I	Monotherapy	High blood lactate, neurotoxicity	NCT02882321
		PDK	Dichloroacetate	Head and neck squamous cell carcinoma	II	With cisplatin/radiotherapy	Similar survival and AEs	NCT01386832
				Breast cancer, NSCLC	II	Monotherapy	Terminated due to safety concerns	NCT01029925
				Glioblastoma multifiorme	II	Monotherapy	Ongoing	NCT05120284
				Glioblastoma multiforme	I	Monotherapy	Well tolerated	NCT01111097
	Adenosine Metabolism	CD73	Oleclumab	Metastatic PDAC	Ib/II	Oleclumab + durvalumab	ORR 32.9%; PFS HR = 0.72 (95% CI 0.47–1.11); high-CD73 subgroup may benefit; ≥Grade 3 AEs: 77.1%	NCT03611556
				NSCLC	II	With PD-L1 blocker	MPR: 19.0%. Safety manageable, no new signals	NCT03794544, NCT03822351;^484,485^
				Advanced solid malignancies	I	Monotherapy	Safety, tolerability, and PK assessed in 6 patients; no efficacy results posted	NCT03736473
				Unresectable stage III NSCLC	III	Durvalumab, chemo-radiotherapy	Recruiting	NCT05221840
				Solid tumors	I	With durvalumab + IPH5201 (anti-CD39)	Safety, PK, and immunogenicity assessed in 57 patients; no efficacy results posted	NCT04261075
				NSCLC	II	With AZD4635 (A2AR antagonist)	ORR 11.1% (3/27), manageable safety, no new safety signals	NCT03381274
				Solid tumors	II	With durvalumab + AZD4635	PSA50 response 0% (0/17), limited efficacy, manageable safety	NCT04089553
			CPI-006	Solid tumors	I	CPI-006 alone + pembrolizumab/ciforadenant	Dose-limiting toxicities/Treatment efficacy	NCT03454451
		CD39	IPH5201	Resectable NSCLC	II	Durvalumab, chemotherapy (neoadjuvant and adjuvant)	Recruiting	NCT05742607
			TTX-030	Metastatic pancreatic adenocarcinoma	II	Chemotherapy ± budigalimab	Active, not recruiting	NCT06119217
		ADORA2A	Taminadenant	Advanced/metastatic NSCLC (≥1 prior therapy)	I/Ib	With or without spartalizumab (anti-PD-1)	MTD established. Safety manageable, modest efficacy signals	NCT02403193^486^
			AZD4635	Solid tumors	Ia/Ib	With PD-L1 blocker	Well tolerated; objective responses in mCRPC; median PFS 10.1–14.9 weeks; PSA responses observed	NCT02740985^487^
				Advanced solid malignancies	I	Monotherapy	Completed, no results posted	NCT03980821
				metastatic Castrate-Resistant Prostate Cancer (mCRPC)	I	AZD4635 + Durvalumab + Cabazitaxel	Safety manageable, modest efficacy signals. Median rPFS 5.8 months. PSA50 responses in 5/28	NCT04495179
			SCH58261	Breast cancer, melanoma, leukemia	Preclinical	With CAR-T cells or ICBs	Enhances CAR-T-cell cytokine production & antitumor efficacy; no autoimmune pathology observed	^488–490^
			Ciforadenant	Multiple myeloma	Ib	Oral A2AR inhibitor + IV anti-CD38 mAb	Safety/tolerability assessed; ORR, DOR, DCR evaluated. Study completed (n = 7)	NCT04280328
				Renal cell carcinoma	II	With nivolumab + ipilimumab	Recruiting	NCT05501054
				RCC, mCRPC	I/Ib	With atezolizumab	Safety/tolerability, ORR assessed. Study completed (n = 502)	NCT02655822
			Etrumadenant (AB928)	Metastatic colorectal cancer	I/II	Zimberelimab, mFOLFOX-6, bevacizumab	Manageable toxicity; improved PFS and OS	NCT04660812^491^
		Adenosine enzyme axis	PEG-ADA	Breast cancer, melanoma, lung cancer	Preclinical	Combined with ICBs	Reduced tumor growth, enhanced CD8^+^ T-cell activity, improved response to anti-PD-1 therapy	^492^
	Hypoxia Metabolism	Anti-hypoxic	Trans sodium crocetinate	Glioma	II	TSC + SOC (radiation/temozolomide)	Median OS 11.3 mo. High AE rates. Trial terminated early (n = 19)	NCT03393000
		HIF-1/2	MBM-02	Prostate cancer, GBM, metastatic cancer	II	Oral HIF-1/2 inhibitor monotherapy	PSA reduction ≥50% assessed. Study status unknown/not yet recruiting	NCT04876755, NCT04874506
			BAY 87-2243	Advanced malignancies	I	Oral HIF-1α inhibitor monotherapy	MTD, safety, PK assessed. Study terminated early (n = 5)	NCT01297530
		HIF-1α	EZN-2968 (antisense)	Solid tumors, lymphoma	I	IV antisense oligonucleotide monotherapy	HIF-1α mRNA modulation assessed. Study completed (n = 10)	NCT01120288
			PX-478	Advanced metastatic solid tumors	I	Oral HIF-1α inhibitor monotherapy	MTD, safety, PK, and PD of HIF-1α pathway assessed. Study completed	NCT00522652
			RO7070179	Hepatocellular carcinoma	Ib	IV antisense oligonucleotide monotherapy	HIF1A mRNA reduction assessed as proof-of-mechanism. Study completed (n = 9)	NCT02564614
		HIF-2α	DFF332	Advanced/relapsed renal cancer	I	DFF332 ± Everolimus ± (Spartalizumab + Taminadenant)	Safety, DLTs, ORR assessed. Active, not recruiting (enrollment halted)	NCT04895748
			NKT2152	ccRCC	I/II	Oral HIF-2α inhibitor monotherapy	MTD/RDE, safety, ORR assessed. Study terminated (portfolio reprioritization) (n = 120)	NCT05119335
				ccRCC	II	With sasanlimab + palbociclib	DLTs, ORR assessed. Study terminated (portfolio reprioritization) (n = 18)	NCT05935748
			MK-3795 (PT-2385)	RCC, glioblastoma	II	Oral HIF-2α inhibitor monotherapy	ORR, PFS, DOR assessed. Study completed (n = 4)	NCT03108066
				ccRCC	I	Oral HIF-2α inhibitor monotherapy or in combo (ICB/TKI)	MTD/RP2D, safety, PK, ORR assessed. Active, not recruiting	NCT02293980
			Belzutifan (MK-6482)	Solid tumors	III	Oral HIF-2α inhibitor vs Everolimus	PFS 5.6 vs 5.6 mo, ORR 21.9% vs 3.5%. Improved HRQoL. Anemia common	NCT04195750
				Solid tumors	III	With pembrolizumab	Active, not recruiting	NCT05899049
	Other Metabolism	AMPK	Metformin	Solid tumors	II	With nivolumab or pembrolizumab	Recruiting. Assessing ORR, PFS, OS, safety, and immunologic correlatives	NCT04114136
				Ovarian clear cell carcinoma	II	With zimberelimab	Unknown status. Primary: ORR. Secondary: PFS, OS, DCR, safety	NCT05759312
				SCLC	II	With sintilimab	Unknown status. Primary: ORR, safety. Secondary: OS, PFS, DOR	NCT03994744
				NSCLC	II	With nivolumab	ORR: 23.5% (4/17). DCR: 47% (8/17). Median PFS: 1.8 m. Median OS: 7.2 m. Safety acceptable	NCT03048500
		Antioxidant	Acetyl cysteine	Solid tumors	II	Topotecan ± Acetylcysteine	Terminated (enrolled 1 patient). No efficacy data. Safety: multiple grade 1-2 AEs reported	NCT02569957
				Lymphoma	I	With CAR-T	Active, not recruiting	NCT05081479
		ROS	MnTE-2-PyP5+	Breast cancer	Preclinical	MnTE alone	Inhibits M2 polarization, reduces cancer cell growth and T-cell suppression in vitro	^493^
			GB111-NH2	Breast cancer	Preclinical	Monotherapy	Induced TAM apoptosis and oxidative stress, tumor regression	^494^
MIC-III	Microbiome Metabolism	Gut microbiota modulation	High-fiber diet (microbiota modulation)	Metastatic/unresectable stage III–IV melanoma	II	With pembrolizumab or nivolumab	Active, not recruiting	NCT04645680^495^
			Camu-camu berry powder	Advanced NSCLC, melanoma	Recruiting	Standard-of-care ICB (pembrolizumab/nivolumab ± ipilimumab)	Recruiting	NCT05303493
		Serotonin transporter	Fluoxetine	CRC	I	Neoadjuvant	Not yet recruiting	NCT06225011
		Bile acids/SCFAs/tryptophan metabolites	Butyrate/bile acid modulation/probiotics/FMT	Solid tumors, CRC, liver cancer	Preclinical	Dietary/supplementation/microbiome intervention ± ICB	Recruiting	^310–318,367^

*MIC-I* Metabolic immune checkpoint type I, *MIC-II* Metabolic immune checkpoint type II, *MIC-III* Metabolic immune checkpoint type III, *PD-L1* Programmed death-ligand 1, *OGT* O-GlcNAc transferase, *HGS* Hepatocyte growth factor-regulated tyrosine, *CRC* Colorectal cancer, *LDHA* Lactate dehydrogenase A, *NSCLC* Non-small cell lung cancer, *HK2* Hexokinase 2, *NF-κB* Nuclear factor kappa-light-chain-enhancer of activated B cells, *2-DG* 2-Deoxy-D-glucose, *PD-1* Programmed cell death protein 1, *AMPK* AMP-activated protein kinase, *EZH2* Enhancer of zeste homolog 2, *CTLA-4* Cytotoxic T-lymphocyte-associated protein 4, *ICB* Immune checkpoint blockade, *CPT1A* Carnitine palmitoyltransferase 1A, *DES* Diethyl succinate, *DMK* Dimethyl α-ketoglutarate, *ACLY* ATP citrate lyase, *HCC* Hepatocellular carcinoma, *HDAC2* Histone deacetylase 2, *YY1* Transcription factor YY1, *SQLE* Squalene epoxidase, *GLUT1* Glucose transporter type 1, *PFKFB3* 6-Phosphofructo-2-kinase/fructose-2,6-biphosphatase 3, *MCT1* Monocarboxylate transporter 1, *MCT4* Monocarboxylate transporter 4, *LDH* Lactate dehydrogenase, *ATP* Adenosine triphosphate, *MDSCs* Myeloid-derived suppressor cells, *COX-2* Cyclooxygenase-2, *PTGER2* Prostaglandin E receptor 2, *TNBC* Triple-negative breast cancer, *cDC1* Conventional dendritic cell type 1, *PTGER4* Prostaglandin E receptor 4, *CD36* Cluster of differentiation 36, *GBM* Glioblastoma, *FASN* Fatty acid synthase, *ORR* Objective response rate, *PFS* Progression-free survival, *AEs* Adverse events, *THBS1* Thrombospondin 1, *LXR* Liver X receptor, *ABCA1* ATP-binding cassette transporter A1, *EMT* Epithelial-mesenchymal transition, *TAM* Tumor-associated macrophage, *PPARγ* Peroxisome proliferator-activated receptor gamma, *CB2* Cannabinoid receptor 2, *DHEA* Dehydroepiandrosterone, *EPEA* Eicosapentaenoylethanolamide, *IL-6* Interleukin-6, *LPS* Lipopolysaccharide, *HDAC* Histone deacetylase, *IDO1* Indoleamine 2,3-dioxygenase 1, *MDS* Myelodysplastic syndromes, *UC* Urothelial carcinoma, *OR* Objective response, *SD* Stable disease, *USP14* Ubiquitin specific peptidase 14, *Trp* Tryptophan, *mTOR* Mechanistic target of rapamycin, *DIPG* Diffuse intrinsic pontine glioma, *ASCT2* Alanine-serine-cysteine transporter 2, *GLS1* Glutaminase 1, *RCC* Renal cell carcinoma, *Bev* Bevacizumab, *Pac* Paclitaxel, *Capiva* Capivasertib, *LAT2* L-type amino acid transporter 2, *NOS* Nitric oxide synthase, *L-NMMA* N(G)-monomethyl-L-arginine, *PDH* Pyruvate dehydrogenase, *OGDH* Oxoglutarate dehydrogenase, *OS* Overall survival, *mIDH1* Mutant isocitrate dehydrogenase 1, *GemCis* Gemcitabine/Cisplatin, *AML* Acute myeloid leukemia, *ETC* Electron transport chain, *PDK* Pyruvate dehydrogenase kinase, *CD73* Cluster of differentiation 73, *MPR* Major pathologic response, *PK* Pharmacokinetics, *ADORA2A* Adenosine A2a receptor, *MTD* Maximum tolerated dose, *mCRPC* Metastatic castration-resistant prostate cancer, *PSA* Prostate-specific antigen, *rPFS* Radiographic progression-free survival, *CAR-T* Chimeric antigen receptor T-cell, *IV* Intravenous, *mAb* Monoclonal antibody, *DOR* Duration of response, *DCR* Disease control rate, *PEG-ADA* Pegylated adenosine deaminase, *TSC* Trans sodium crocetinate, *SOC* Standard of care, *HIF-1/2* Hypoxia-inducible factor 1/2, *DLTs* Dose-limiting toxicities, *TKI* Tyrosine kinase inhibitor, *HRQoL* Health-related quality of life, *FMT* Fecal microbiota transplantation, *SCFAs* Short-chain fatty acids

IDO1 inhibitors represent another class of promising MIC-targeted agents. In phase I/II clinical trials, IDO inhibitors restored T-cell antitumor capacity by inhibiting tryptophan metabolism and reducing T-cell exhaustion.^411^ However, in phase III clinical trials, epacadostat combined with the PD-1 antibody pembrolizumab failed to improve progression-free survival (PFS) compared with monotherapy.^412^ These findings suggest a key limitation: targeting just one metabolic node often fails to deliver lasting results. This is largely due to redundancy and compensatory mechanisms within the tryptophan pathway. In the TME, inhibiting IDO1 frequently triggers compensatory reprogramming. A common response is the adaptive upregulation of the TDO2 pathway. This stems from the metabolic plasticity of tumor cells and dynamic feedback in the microenvironment. When IDO1 is suppressed, local tryptophan can accumulate. This accumulation activates stress-responsive signaling, which then induces TDO2 transcription.^152^ Notably, this upregulation may not be limited to tumor cells. It can also occur within stromal components such as matrix fibroblasts. These TDO2-positive fibroblasts can then enhance tumor progression. They generate kynurenine, which promotes ferroptosis resistance in metastatic cells. Concurrently, this kynurenine impairs T-cell function.^413^ Therefore, this metabolic adaptation sustains immunosuppressive kynurenine production through enzymatic switching. It also highlights the inherent limits of targeting a single node in tumors with high pathway redundancy. Furthermore, multiple parallel mechanisms, including adenosine signaling, the PD-1/PD-L1 axis, regulatory T cells, and suppressive myeloid populations, collectively sustain tumor-associated immunosuppression. The absence of predictive biomarkers that can identify patient subsets truly dependent on tryptophan metabolism further compromises the clinical effectiveness of such targeted approaches.

In addition, ACLY inhibitors, as an emerging therapeutic strategy, have dual immune regulatory effects. On the one hand, ACLY inhibition can upregulate PD-L1 expression in tumor cells, promoting T-cell exhaustion and immune suppression; on the other hand, it can induce mitochondrial damage and polyunsaturated fatty acid (PUFA) peroxidation, leading to mtDNA leakage into the cytoplasm and activation of the cGAS-STING pathway, thereby enhancing antitumor immune responses. However, since ACLY is also involved in FAS in normal tissues such as the liver and adipose tissue, long-term systemic inhibition may cause lipid metabolic disorders and systemic toxicity. Importantly, excessive activation of cGAS-STING signaling has been shown to drive immune checkpoint inhibitor-associated myocarditis through Gasdermin-E-mediated pyroptosis,^414^ underscoring the narrow safety margin of metabolic-immune activation. These findings indicate that ACLY inhibitors simultaneously affect both MIC-I metabolic immune checkpoints and MIC-II enzyme-dependent immune regulation, highlighting the need for tumor-targeted delivery strategies to balance antitumor efficacy with systemic safety.^124^

Existing targeted drugs and related therapies against MICs face a series of common challenges, including significant AEs, difficulties in patient stratification for personalized therapy, high costs and barriers to broad clinical translation. These challenges stem from the ubiquitous yet highly heterogeneous nature of metabolic pathways across tumor cells versus normal cells, as well as among different immune cell subsets. First, AEs represent a major bottleneck in MIC drug development. For example, inhibitors targeting key metabolic nodes, such as HK2 inhibitors and ACLY inhibitors, often cause off-target toxicity due to the essential roles of these enzymes in normal physiological metabolism, thereby impairing vital organs such as the heart and liver. Second, personalized treatment remains challenging. Tumor metabolism exhibits pronounced intra- and intertumoral heterogeneity and is tightly intertwined with the immune microenvironment. Patient responses to specific MIC inhibitors are influenced by multiple factors, including tumor metabolic profiles, immune cell composition, and host genetic background; however, reliable predictive biomarkers to precisely identify responsive populations are still lacking. Immune cell metabolism is highly plastic under pathological conditions. For instance, systemic metabolic dysregulation of T cells has been observed in severe viral infection and can be partially reversed by pharmacological intervention, highlighting both the therapeutic potential and systemic risks of metabolic modulation.^415^ Furthermore, high costs limit accessibility. The development of novel metabolic drugs, complex manufacturing processes and advanced delivery technologies designed to improve targeting (such as nanocarriers) all contribute to increased treatment costs. In addition, combination therapy strategies (e.g., MIC inhibitors combined with ICBs), although increasingly common, further increase economic burden and therapeutic complexity. Finally, barriers to widespread application also include an incomplete understanding of the network-level regulation of MICs, metabolic differences between preclinical models (such as mice) and humans and the complex regulatory pathways required for approval of new therapies.

To address these challenges, future development paths are now more defined. A critical approach involves developing cell-specific targeting strategies to overcome toxicity limits. Nanotechnology-based delivery systems can use features of the TME or specific cell surface markers. This allows for precise drug accumulation at disease sites. Prodrug strategies and bispecific molecules also show promise for widening the therapeutic window. At the same time, in-depth dissection of compensatory mechanisms within metabolic networks and the promotion of multitarget combination interventions may help overcome the limitations of inhibiting single metabolic nodes. Most importantly, the establishment of multiomics-based biomarker systems to enable refined patient stratification will ensure that therapies are applied to populations most likely to benefit. The integration of cutting-edge technologies such as high-throughput single-cell omics and spatial metabolomics will profoundly reveal the dynamic landscape of metabolic-immune interactions within the TME.^111–116^ Multidimensional single-cell analyses of tumor-infiltrating immune cells can precisely elucidate the links between specific metabolic states and immune functions, identify novel actionable targets, and guide personalized therapeutic strategies. Together, these technological advances will drive the development of next-generation MIC-targeted therapies, improving efficacy while better controlling toxicity and ultimately enabling a substantive transition from basic research to clinical benefit.

Similarly, translating preclinical discoveries into human trials represents a major bottleneck in the development of MIC-based therapies and is primarily hindered by three obstacles. First, substantial interspecies physiological differences exist: mice and humans differ fundamentally in metabolic rates, immune cell composition, and regulatory networks, making it difficult to accurately extrapolate drug efficacy and toxicity from animal models to humans. Second, preclinical models often fail to capture the full complexity of the TME, including the high heterogeneity, long-term evolutionary processes, and spatiotemporal dynamics of metabolic-immune interactions in human tumors, causing many strategies effective in simplified models to fail in the human context. Third, compensatory mechanisms and individual heterogeneity pose additional challenges. Humans exhibit greater metabolic plasticity and immune redundancy, such that inhibition of a single MIC node may readily trigger bypass activation; moreover, interpatient differences in genetic background, organ function and comorbidities significantly influence therapeutic response windows. To overcome these barriers, further development of humanized models and organoid-immune coculture systems is needed, along with the integration of multiomics biomarkers into adaptive designs in early-phase clinical trials, thereby establishing an effective translational bridge from the laboratory to the clinic.

### Innovative combination strategies

Based on an increasingly sophisticated understanding of the MIC network, researchers have developed a range of innovative combination intervention strategies.^416–420^ These approaches aim either to simultaneously target multiple metabolic inhibitory nodes or to enhance the resistance of immune cells to suppressive microenvironments, thereby improving the efficacy of cancer immunotherapy. Current strategies can be broadly categorized into the following types.

#### Combination of metabolic drugs and immunotherapies

This approach aims to reshape the immunosuppressive TME through metabolic interventions, thereby enhancing the therapeutic efficacy of immune-based treatments such as ICBs. A representative example is the combination of the glutaminase inhibitor CB-839 (Telaglenastat) with PD-1 blockade. CB-839 works by blocking glutamine metabolism in tumor cells. This significantly disrupts the production of amino acids and nucleotides, which suppresses tumor growth. Mechanistically, this targets type II MICs. It limits nutrients for tumor cells while also changing the immune microenvironment. Clinical research has tested its potential synergy with ICBs. A phase I/II trial included patients with advanced solid tumors. The combination of CB-839 and nivolumab showed different results across tumor types and prior treatments. For patients with clear cell renal cell carcinoma (ccRCC) who had not received prior checkpoint inhibitors, the objective response rate (ORR) was 24%. However, no responses were observed in NSCLC patients who had previous anti-PD-1/PD-L1 therapy. These results indicate that the combination’s success may depend heavily on the context. Factors such as tumor type and prior immune therapy exposure influence it. This highlights the complex and variable role of metabolic changes within different immune-metabolic settings.^416^ Therefore, CB-839 serves as an example of an MIC-II-targeted method. It modifies tumor immune evasion pathways, suggesting a reasonable path for combination immunotherapy.

#### Nanodelivery systems

To enhance therapeutic efficacy while reducing systemic toxicity, researchers have increasingly focused on developing novel nanodelivery systems capable of targeting the TME. One example uses pH-sensitive nanocarriers. These carriers release drugs specifically in the acidic conditions of tumors. This boosts treatment at the tumor site while greatly reducing damage to healthy organs such as the liver and kidneys. A more advanced example uses nanoparticles made from a polydopamine-coated copper and alpha-ketoglutarate co-polymer(CKPP). This system increases ROS production and controls cell metabolism. It enables a combined cancer immunotherapy that triggers both necroptosis and cuproptosis. Mechanistically, this method changes cell energy production from glycolysis to OXPHOS. This change enhances a form of cell death caused by copper ions. By regulating type II MICs and driving metabolic reprogramming, this approach has shown strong antitumor results in mouse models of colorectal cancer.^417^ The benefits of nanodelivery are better targeting accuracy, less off-target harm, and the possibility of delivering several drugs together for a combined effect. However, challenges still exist. These include complicated manufacturing steps, the difficulty of large-scale production, concerns about long-term safety inside the body and the risk of triggering an immune response.

#### Targeting the metabolic microenvironment to reprogram immune cells

This strategy directly modifies immune cells to enable resistance to metabolic suppression within the TME, thereby maintaining or enhancing their effector functions. For instance, gene silencing approaches that reduce the expression of MCT4 in NK cells enhance their resistance to lactate-mediated immunosuppression.^420^ This represents a targeted intervention against type II MICs, allowing NK cells to remain functional in the lactate-rich TME and more effectively control tumor progression. The advantages of this approach lie in its localized and immune cell-specific nature. However, it also faces challenges related to the safety of gene-editing technologies, the complexity of immune cell manufacturing and the durability and persistence of modified cells in vivo.

#### Engineered metabolically enhanced immune cell therapies

Engineered cell therapies, particularly CAR-T-cell therapy, represent a major breakthrough in cancer immunotherapy. However, metabolic constraints within the TME, such as nutrient depletion and accumulation of suppressive metabolites, are key contributors to CAR-T-cell exhaustion and limited persistence. Consequently, metabolic reprogramming of CAR-T cells through synthetic biology has emerged as a frontier strategy to enhance therapeutic efficacy. A representative approach involves engineering CAR-T cells to overexpress peroxisome proliferator-activated receptor gamma coactivator 1α (PGC1α), a central regulator of mitochondrial biogenesis and oxidative metabolism. Studies have shown that CAR-T cells overexpressing PGC1α exhibit enhanced mitochondrial metabolic activity, increased oxidative phosphorylation capacity, and a memory-like phenotype. In preclinical models, these metabolically enhanced CAR-T cells demonstrate prolonged in vivo persistence and superior antitumor efficacy, particularly under conditions of glucose restriction.^418,419^ This strategy fundamentally strengthens the cellular energy factory of T cells, enabling them to better adapt to metabolic challenges within the TME and constituting a precise modulation of type II MICs. An alternative approach involves engineering CAR-T cells to express specific metabolic enzymes to overcome microenvironmental suppression. For example, CAR-T cells expressing lactate dehydrogenase A may function more effectively in lactate-rich environments, while the expression of enzymes capable of degrading adenosine, such as adenosine deaminase or inhibitors of CD39/CD73, may confer resistance to adenosine-mediated immunosuppression. Despite their promise, metabolically enhanced CAR-T therapies face several challenges. The complexity of metabolic pathways means that excessive or inappropriate engineering may disrupt normal cellular physiology or even induce autoimmune risks. Moreover, metabolic enhancement must be carefully balanced to ensure that antigen-specific cytotoxicity is preserved without causing uncontrolled proliferation. Finally, challenges related to manufacturing standardization, regulatory approval pathways, and high costs remain significant barriers to clinical translation. Future research will need to more precisely define tumor-specific metabolic barriers across diverse TMEs and design more refined, controllable metabolic engineering strategies accordingly.

### Biomarkers and patient stratification

Given the extreme complexity of the MIC network and the pronounced interpatient and intratumoral heterogeneity, the development of precise, dynamic, and clinically translatable biomarker systems has become a central bottleneck and key driving force for translating MIC-targeted therapies from conceptual frameworks into routine clinical practice. An effective biomarker system should not only enable static patient stratification but also dynamically capture adaptive changes in the metabolic-immune network of the TME in response to therapeutic intervention, thereby guiding real-time treatment adjustment. To this end, a multilayered and dynamic biomarker integration framework that incorporates spatial, liquid, and single-cell dimensions is gradually transitioning from concept to clinical validation.

#### Radiomics: mapping the spatial landscape of tumor metabolic immunity

Conventional imaging modalities, such as ^18^F-FDG PET/CT, capture aberrant glucose uptake by tumor cells and provide macroscopic information on overall tumor metabolic burden. However, for MIC research, a critical advance lies in the development of multimodal molecular imaging techniques capable of specifically resolving immune cell metabolic states and their spatial distribution within tumors.

##### Immune cell-specific imaging

PET imaging using radiotracers targeting immune cell surface markers (such as CD8, CD4, and PD-L1) enables direct and non-invasive quantification of the abundance and spatial distribution of distinct immune cell subsets within tumors. As described above, the combined application of ^89^Zr-Df-IAB22M2C (an anti-CD8 nanobody) and ^18^F-FDG allows not only dynamic assessment of changes in CD8^+^ T-cell infiltration before and after treatment but also colocalization analyses that reveal spatial relationships between highly metabolic tumor regions and immune hot or cold zones. This provides intuitive evidence for how metabolic suppression contributes to immune exclusion within the TME. Notably, clinical studies have shown that after 14 days of nivolumab treatment, CD8^+^ T-cell infiltration in the primary tumor significantly increased (SUV_max_=19.3), which was significantly correlated with a treatment response lasting over three years.^421^ In the field of liquid biopsy, plasma metabolomics analysis identified succinate levels (a MIC-II-related metabolite) as a potential biomarker for predicting PD-1 inhibitor efficacy.^261^

##### Metabolic pathway-specific imaging

Beyond glucose metabolism, novel tracers targeting specific metabolic pathways are under active development. For example, ^11^C-acetate and ^11^C-choline PET can reflect lipid biosynthesis,^422,423^ while ^18^F-FACBC enables the assessment of amino acid transport.^424^ The combined use of these tracers can generate a multinutrient metabolic profile of tumors, identifying those that are highly dependent on glutamine or fatty acids and thereby predicting potential sensitivity to corresponding MIC inhibitors, such as glutaminase or ACLY inhibitors.

##### Radiomics and artificial intelligence

High-throughput extraction of imaging features, including texture, shape, and intensity, combined with artificial intelligence (AI) algorithms allows radiomics to convert images into high-dimensional, mineable datasets.^425,426^ This approach facilitates the identification of imaging patterns associated with specific MIC states or therapeutic responses that are imperceptible to the human eye. A practical example involves deep learning models. These models can analyze pretreatment PET/CT images. Based on this analysis, they may predict the probability that a tumor will respond to a combination therapy that includes an IDO inhibitor.

#### Liquid biopsy: capturing systemic and dynamic metabolic–immune signals

Liquid biopsy, particularly blood-based assays, provides a unique opportunity for repeated sampling and real-time monitoring of systemic tumor and immune microenvironmental changes.

##### Circulating metabolomics and lipidomics

Systematic profiling of small-molecule metabolites in plasma or serum reflects the integrated metabolic state of both the host and tumor. As discussed above, circulating succinate levels have been associated with responses to immunotherapy. In addition, other metabolites, including the kynurenine/tryptophan ratio (reflecting IDO activity), lactate levels, and specific lipid species such as ceramides and prostaglandins, may serve as systemic biomarkers of MIC activity. Dynamic monitoring of these metabolites during treatment may enable early identification of therapeutic response or resistance.

##### Circulating tumor DNA and metabolic genes

Circulating tumor DNA (ctDNA) analysis not only tracks tumor clonal evolution but also captures mutations in metabolism-related genes (such as IDH1 R132H, FH, and SDH loss-of-function mutations), which themselves represent powerful predictive biomarkers.^427–429^ For example, gliomas harboring IDH1 mutations respond poorly to ICBs but show high sensitivity to IDH1 inhibitors, supporting genotype-guided metabolic stratification strategies.^430,431^ Moreover, ctDNA methylation patterns and fragmentomics may indirectly reflect tumor metabolic reprogramming states.^432^

##### Extracellular vesicles and cytokine profiles

Extracellular vesicles released by tumor and immune cells carry proteins, nucleic acids, and metabolites and function as mediators of intercellular communication.^433^ Analysis of their cargo can provide a molecular snapshot of the metabolic state of the cells of origin. In parallel, serum cytokine and chemokine profiles (e.g., IL-6, IFN-γ, TGF-β) reflect systemic immune status, and their integration with metabolic biomarkers may enhance predictive performance.

#### Single-cell and spatial multiomics: decoding TME heterogeneity at high resolution

A comprehensive understanding of MIC function at the cellular level requires single-cell and spatial multiomics technologies.

##### Identification of novel MIC-related cellular subsets

Single-cell RNA sequencing (scRNA-seq) enables fine-grained resolution of functional states within T cells, macrophages, and other immune populations. When combined with emerging single-cell metabolomics approaches or inferred metabolic activity from scRNA-seq data using metabolic gene set scoring, specific immune transcriptional phenotypes can be directly linked to metabolic programs.^434^ For instance, the identification of CD8^+^ T-cell subsets coexpressing exhaustion markers (PD-1, TIM-3) and glycolytic genes suggests that functional impairment may be closely tied to glucose metabolic imbalance,^435^ highlighting a metabolically defined MIC mechanism and a targetable immune subset.

##### Deciphering intercellular metabolic interactions

Spatial transcriptomics (e.g., Visium, MERFISH)^436^ and spatial metabolomics (e.g., mass spectrometry imaging)^437^ preserve tissue architecture while enabling analysis of which cells, such as tumor cells or cancer-associated fibroblasts, produce immunosuppressive metabolites (e.g., adenosine, lactate) and which neighboring immune cells exhibit functional suppression. Such spatial correlations provide direct in vivo evidence of MIC activity and help identify localized metabolic immune deserts requiring targeted intervention.

##### Discovery of predictive features and actionable targets

Comparative analysis of paired pre- and post-treatment single-cell datasets enables identification of metabolic pathways or cellular subsets that change specifically in responders. These features may serve as predictive biomarkers. More importantly, such analyses can uncover novel, actionable MIC targets, such as enzymes expressed by specific myeloid subsets that drive immunosuppressive metabolite production.

#### Integration and dynamic monitoring: toward adaptive therapeutic navigation

Future progress will not rely on any single technology alone. Instead, it will depend on integrated, multimodal, and dynamic monitoring frameworks.

##### AI-driven multiomics integration models

Machine learning and systems biology approaches can integrate data from imaging, liquid biopsy, tissue multiomics, and even the microbiome to build composite models predicting patient responses to specific MIC-targeted therapies. For example, an AI platform may integrate (1) imaging-derived tumor metabolic heterogeneity features from PET^438^; (2) plasma metabolite signatures^439^; and (3) proportions of immunosuppressive macrophage/exhausted T-cell populations defined by single-cell sequencing.^440^ The model outputs an MIC-targeted therapy benefit score to guide clinical trial enrollment or treatment decisions. Early studies have begun exploring such integrative models, combining ctDNA,^441^ metabolite profiles,^439^ and immune cell flow cytometry data to predict immunotherapy response.^442^

##### Dynamic biomarkers and adaptive therapy

The MIC network is highly adaptable. Therapeutic pressure causes compensatory adaptation and tumor evolution. Therefore, static baseline biomarkers are not sufficient. Future clinical practice needs to include dynamic monitoring. This monitoring should occur at key treatment time points, such as two to four weeks after starting. For example, consider a patient on combined IDO inhibition and PD-1 blockade. A liquid biopsy can check if kynurenine levels remain low.^443^ At the same time, imaging can assess CD8^+^ T-cell infiltration into the tumor core.^444^ The results may show divergent patterns. One pattern could be metabolic escape, where kynurenine rises despite good immune infiltration. This pattern suggests metabolic escape, indicating that continuing the current treatment may be suboptimal and that metabolic pathway adjustment should be considered. Another pattern could show no change in either kynurenine levels or immune infiltration. This pattern may indicate primary resistance. In that case, switching the treatment regimen would be warranted. This monitor-and-adjust adaptive approach is crucial. It is essential for managing tumor evolution and for achieving true precision medicine.

##### Clinical trial exploration

Multiple prospective trials are actively investigating these novel biomarkers. Basket trials are enrolling pancancer patients with specific metabolic gene alterations (e.g., IDH1, FH/SDH) to test targeted agents while collecting multidimensional biomarker data, including advanced imaging and liquid biopsy, to identify predictors of response.^445^ Other studies aim to determine whether early PET metabolic response or ctDNA clearance-guided adjustment of immunotherapy combinations can improve patient outcomes.^446,447^

In summary, developing effective biomarkers is central to realizing the full potential of MIC-targeted therapies. These biomarkers come from several advanced technologies. Spatially resolved radiomics provides detailed imaging data. Non-invasive liquid biopsy allows for dynamic tracking. Single-cell and spatial multiomics offer deep mechanistic insights. Together, these technologies create a comprehensive monitoring system. Integrating these data with AI is the next step.^448^ This AI-driven integration enables dynamic, biomarker-guided treatment adaptation. It allows for precise patient stratification, matching individuals to the MIC interventions most likely to help them. Furthermore, treatment strategies can be optimized in real time based on patient response. Ultimately, this integrated approach is likely to improve the clinical success rate of metabolic immunotherapy. It will also speed up the translation of these therapies from research to routine patient care.

## Conclusion and future perspectives

### Conclusion

Research on tumor MICs provides a fundamentally new perspective for understanding immune evasion and offers transformative opportunities for cancer therapy. In this work, we systematically introduce the emerging concept of MICs. Our aim is to bridge the longstanding gap between metabolic research and immunology. We establish an integrative classification framework with three types. These are type I, type II, and type III MICs. This framework reveals distinct but connected ways metabolism regulates antitumor immunity. MIC-I describes how metabolic modifications drive the regulation of standard immune checkpoints. MIC-II refers to metabolic enzymes or metabolites acting as direct immune regulatory nodes. MIC-III captures microbiome-derived metabolic signals that indirectly shape immune responses. Within this framework, the rewiring of basic metabolic pathways shapes the immune landscape. These pathways include glucose, lipid, and amino acid metabolism. Mitochondrial dysfunction and key signaling axes, such as HIF, NAD^+^, and ROS, are also involved. They dynamically shape immune cell fate and function through complex interaction networks. This process drives tumor immune evasion. This conceptual framework deepens our understanding of the TME by framing it as a central hub for metabolic-immune crosstalk. It also organizes previously scattered metabolic observations into a clear, targetable paradigm. This fundamentally reshapes our system-level view of tumor-immune interactions. Importantly, this review connects mechanistic insights directly to clinical use. We summarize recent advances and challenges in the development of MIC-targeting therapeutics and highlight several highly promising translational strategies, including the combination of metabolic modulators with ICBs, nanotechnology-based targeted delivery systems, and metabolic reprogramming of immune cells through synthetic biology approaches, such as metabolically enhanced CAR-T cells. We further emphasize that the development of multilayered biomarker systems integrating radiomics, liquid biopsy approaches, and single-cell multiomics is essential for precise patient stratification and therapeutic response prediction. Collectively, this work establishes a comprehensive knowledge framework spanning theoretical concepts, molecular mechanisms, targeting strategies, and clinical translation, providing a solid foundation for the development of next-generation precision immunotherapies based on MIC regulation.

### Future directions

Although research on MICs has made significant progress in forming concepts and exploring mechanisms, the field is still expanding rapidly. Its full biological complexity and clinical potential are not yet fully realized. Future efforts must advance in both basic science and translational research, with the aim of systematically resolving the dynamic regulation of MIC networks across multiple spatial and temporal scales and converting mechanistic insights into predictive and actionable precision therapeutic strategies. As experimental technologies and analytical methods continue to improve, MICs are expected to emerge as a central nexus linking tumor metabolic reprogramming with immune evasion, thereby driving a deeper conceptual transformation in cancer immunotherapy.

#### Frontiers in basic science: from mapping to mechanistic decryption

The integration of single-cell and spatial multiomics technologies is a critical breakthrough. These tools enable simultaneous characterization of cellular metabolic states, transcriptional programs and spatial distribution at the single-cell level. Combining single-cell metabolomics and transcriptomics with spatial imaging techniques, such as mass spectrometry imaging and MALDI-MSI, allows researchers to directly ask how specific metabolites control immune cell behavior in specific tissue areas. For example, spatial analysis might show distinct T-cell groups in lactate-rich tumor regions. These T cells often have reduced effector function. The same analysis can find immune cells in areas with high local adenosine. These cells may show genetic signs of immunosuppression. Such spatially informed maps provide direct insight into a key mechanism. They show how tiny metabolic gradients control immune dysfunction in the TME. Static measurements of metabolite levels are not enough. They cannot capture the full range of immune metabolic activity. Dynamic metabolic flux analysis addresses this by adding a time dimension. Stable isotope tracing is a powerful method here. Using ^13^C-labeled substrates allows scientists to track the real-time flow of key elements such as carbon through metabolic pathways. Applying this to immune cells shows how their metabolic wiring changes dynamically. This happens during activation, exhaustion, or memory formation. For instance, flux analysis might show that early T-cell activation favors sending glucose-derived carbon into building blocks. In contrast, terminal exhaustion might involve a collapse in mitochondrial energy production. This helps identify the critical metabolic points that control immune cell fate changes. Building on these advances, the next step is integrating high-dimensional data into computational models. This allows a systems-level understanding of MIC regulation. Predictive MIC network models can simulate how the whole system responds to targeting specific metabolic nodes. They can also forecast the results of combination strategies. Adding evolutionary and ecological views is also valuable. Seeing the TME as a dynamic ecosystem is helpful. This ecosystem includes tumor cells, immune cells, and stromal cells. This perspective clarifies how metabolic competition, cooperation, and adaptation together drive immune escape and treatment resistance. Such frameworks can explain, for example, how metabolic inhibitors create selective pressure. This pressure changes how tumors use nutrients, which then affects their response to immunotherapy.

#### Translational and clinical frontiers: from proof-of-concept to precision medicine

The central challenge in translation is turning these mechanistic insights into clinical benefit. This will require deeply integrating interdisciplinary technologies and innovative clinical trial designs. AI-driven analyses of large-scale clinical multiomics datasets provide powerful tools for identifying organ- and tumor subtype-specific MIC vulnerabilities. For example, a deep learning model might predict that targeting a certain lipid pathway in pancreatic cancer would work best with chemotherapy or immunotherapy. This could guide the design of personalized combination treatments. At the same time, standard clinical trials organized only by tumor type may not succeed. They might miss the real potential of MIC-targeted strategies. Alternative trial paradigms, such as basket trials based on shared MIC pathway alterations (e.g., IDH1 mutations or elevated succinate levels) or umbrella trials comparing distinct MIC-based combinations within a single cancer type, offer more informative platforms for clinical validation. The success of these approaches will also critically depend on continued innovation in targeted delivery technologies. The development of organ-specific or immune cell-selective nanocarriers is promising. These carriers release drugs only when they bind to specific surface receptors. This approach aims to maximize effect while minimizing whole-body toxicity. For instance, smart nanoparticles could be engineered to release a metabolic inhibitor only after binding to a T-cell receptor. This would allow localized metabolic reprogramming of antitumor immunity without harming the body’s overall metabolic balance. In parallel, growing evidence shows that the microbiome is a key regulator of the metabolism-immunity link. The gut microbiota produces a wide range of immunomodulatory metabolites, including SCFAs, and future clinical studies may explore precision interventions using rationally designed probiotic consortia, personalized dietary regimens, or engineered microbes capable of sustained in situ production of therapeutic metabolites or cytokines, such as IL-15, to systemically enhance antitumor immune responses.

Looking forward, the main vision for MIC research is to fundamentally change cancer immunotherapy. The shift is away from one-size-fits-all treatments toward deeply personalized precision care. This care will be guided by integrated metabolic-immune profiles. Combining spatial metabolomics, single-cell metabolic flux analysis, and AI-powered modeling will make this possible. It will allow the creation of detailed, dynamic maps of the tumor’s metabolic-immune landscape. Building on these maps, progress in intelligent drug design, targeted delivery and synthetic biology will enable precise control. Engineered immune cells could allow real-time, localized reprogramming of metabolic-immune networks inside the tumor. The ultimate goal is to convert immunologically inert, metabolically suppressive cold tumors into metabolically permissive, immune-inflamed hot tumors, thereby enabling durable and potentially curative therapies tailored to each patient. The journey of MIC research has only just started. However, it has already shown a promising path toward more effective, precise, and transformative cancer immunotherapies.
